# Stakeholder perspectives on adolescent sexual and reproductive health service utilization in Uganda’s Busoga region: A qualitative study

**DOI:** 10.1371/journal.pone.0358996

**Published:** 2026-10-01

**Authors:** Noah Nyende, Justine Nanteza, Frank Kiyingi, Miph Musoke

**Affiliations:** 1 Department of Public Health, Nkumba University School of Sciences, Entebbe, Uganda; 2 Department of Disease Control and Environmental Health, Makerere University School of Public Health, Kampala, Uganda; Indiana University School of Medicine, UNITED STATES OF AMERICA

## Abstract

**Background:**

Adolescents in low- and middle-income countries face high rates of unintended pregnancy, sexually transmitted infections (STIs), and unmet sexual and reproductive health (SRH) needs. In Uganda, 24% of girls aged 15–19 years have begun childbearing, yet modern contraceptive prevalence among adolescents remains low (15%). These challenges are particularly severe in the Busoga region, where sociocultural norms, poverty, and limited health system capacity hinder access to adolescent-friendly SRH services. Despite national guidelines, utilization remains low. Few studies have triangulated perspectives from adolescents, health providers, and community/religious leaders. We explored stakeholder views to provide an integrated understanding of factors influencing adolescent SRH service utilization in Busoga, Uganda.

**Methods:**

An exploratory qualitative study was conducted in Iganga and Bugweri districts. Four focus group discussions with 38 adolescents (13–19 years), stratified by sex, age group, and residence, and 12 key informant interviews with health providers and community leaders were conducted. Adolescents were recruited through peer networks, while key informants were purposively selected. Data were collected (November–December 2024), transcribed verbatim, and analyzed inductively using thematic analysis with NVivo 14. Ethical approval and informed consent/assent were obtained.

**Results:**

Adolescents, health providers, and community stakeholders described SRH service utilization as shaped by adolescent motivation alongside persistent barriers. Adolescents showed strong preventive intent, but uptake was limited by misconceptions about contraception, fears of infertility, long distances, lack of privacy, negative provider attitudes, and commodity stock-outs. Providers and community leaders echoed concerns around misinformation, limited parental guidance, moral norms, and structural constraints. All stakeholders converged on the need for confidential, accessible, and peer- and community-based adolescent SRH services.

**Conclusion:**

Adolescents in Busoga are motivated to use SRH services, but intersecting sociostructural and system barriers limit utilization. Strengthening adolescent-friendly services, improving provider responsiveness, addressing misinformation, and expanding community-based delivery are essential to enhance equitable access.

## Introduction

Adolescence is a critical developmental stage marked by rapid physical, psychological, and social transitions that have lasting implications for sexual and reproductive health (SRH) outcomes [1,2]. Globally, adolescents account for a disproportionate burden of unintended pregnancies, unsafe abortions, and sexually transmitted infections (STIs), particularly in low- and middle-income countries [2]. In sub-Saharan Africa (SSA), recent evidence continues to highlight persistent structural, sociocultural, and health system barriers that drive low utilization of SRH services and sustain high rates of teenage pregnancy and HIV [3,4]. Kenyan and Ethiopian studies, for example, identify transport costs, judgmental provider attitudes, restrictive cultural and gender norms, fear of stigma, and lack of privacy as recurring obstacles [5,6].

In Uganda, adolescent SRH indicators remain concerning. Early sexual debut, low contraceptive uptake, and high rates of adolescent pregnancy persist [7–9]. Uganda’s adolescent pregnancy rate (teenage childbearing) is approximately 24% among girls aged 15–19 years, HIV prevalence among 15–19-year-olds is 2.1%, and only 15% [10]. The Busoga Region is particularly affected because of poverty, early marriage norms, and limited services [11]. Recent studies in the region confirm high non-utilization of modern contraceptives and SRH services, influenced by lack of confidence in discussing contraception, perceived inaccessibility of services, and limited interaction with community health workers [12].

Despite national adolescent health guidelines [13], utilization remains low due to stigma, lack of privacy, and misinformation [8,9]. Yet, little is known about how multiple stakeholders, including adolescents themselves, community and religious leaders, and healthcare providers, perceive these barriers and potential solutions within the same sociocultural context. Most existing research has focused on adolescents alone or examined isolated health system factors [4,14]. This study addresses that gap by qualitatively triangulating perspectives from adolescents, community and religious leaders, and health providers in the Busoga Region. This multi-stakeholder approach provides an integrated understanding of how community norms, service delivery practices, and adolescent preferences interact to shape SRH service utilization, thereby offering more nuanced insights for designing locally responsive, adolescent-friendly interventions. This integrated lens complements single-perspective studies and responds to calls for multi-level approaches to adolescent SRH in SSA [4,15].

To address this gap, the present study explored adolescents’ attitudes, perceptions, and preferences regarding SRH services in the Busoga Region of eastern Uganda, alongside the perspectives of community leaders and health providers. By examining convergences and divergences across stakeholder groups, the study sought to identify multilevel barriers and facilitators that can inform the design of contextually appropriate, adolescent-friendly SRH interventions. The study was guided by the Health Belief Model [16] and Andersen’s Behavioral Model [17] to understand how individual perceptions, enabling factors, and sociocultural contexts shape SRH service utilization.

## Materials and Methods

### Study design

An exploratory qualitative design was employed, guided by a constructivist-interpretivist paradigm. This approach was appropriate for examining how adolescents and key community stakeholders construct meaning about SRH service utilization from their lived experiences.

### Study setting

The study was conducted in Iganga and Bugweri districts in the Busoga Region of Eastern Uganda. The Busoga Region is characterized by a mix of rural and urban communities, varying levels of health service availability, and strong cultural and religious influences on adolescent behavior. Community entry was facilitated through district health officials and local council leaders. The study was conducted between 20 November and 18 December 2024 at six rural (Namirali, Kiwerere, Bulubandi, Naitandu B, Bunalwenyi C, Makandwa) and six urban (Kasokoso Central II/III, Nakavule Main, Busesa, Butende, Kagamba) sites in Iganga and Bugweri districts. The two districts were selected because they reflect contrasting health system contexts: Bugweri has limited resources and fragile health infrastructure, whereas Iganga is better resourced with stronger systems and staffing, ensuring representation of diverse conditions influencing adolescent SRH service access and utilization in the region.

### Participants and sampling

Two categories of participants were included in this study: adolescents and key informants. Adolescents were purposively sampled to capture diverse perspectives on SRH service utilization across gender, age, and residential contexts. Specifically, younger female adolescents (13–17 years) and older male adolescents (17–19 years) were selected to reflect gendered patterns in SRH vulnerability and engagement. Younger females are often at earlier stages of sexual debut and face heightened risks related to early pregnancy and barriers to accessing services. At the same time, older males are more likely to be sexually active and engaged in SRH-related decision-making. The overlap at age 17 was intentional to capture transitional developmental experiences between mid- and late adolescence.

Adolescents were further stratified by residence (rural/urban) to account for contextual differences in access to SRH information and services. In total, 38 adolescents participated in four focus group discussions (FGDs), with recruitment facilitated through community mobilizers and youth peer leaders. Inclusion criteria for adolescents were: aged 13–19 years, residence in the study area for at least 6 months, and willingness to participate.

Key informants were selected using purposive sampling based on their roles, experience, and knowledge of adolescent SRH. These included health providers (such as nurses and clinical officers delivering SRH services) and community leaders, including religious and cultural leaders. Religious leaders were drawn from established faith-based institutions (churches and mosques) and held formal roles (clergy, pastors, or imams). In contrast, cultural leaders were recognized traditional authorities (clan leaders and elders). Health providers contributed perspectives based on their direct experience delivering services to adolescents, while cultural and religious leaders offered insight into the broader sociocultural context shaping adolescent health-seeking behavior. A total of 12 key informant interviews (KIIs) were conducted.

Sampling proceeded iteratively, guided by principles of qualitative inquiry [18], with participant recruitment continuing until no new patterns of meaning emerged from the data. Data saturation was reached after four FGDs and 12 KIIs.

### Data collection

Semi-structured interview guides were developed based on prior literature and national adolescent SRH frameworks [13]. The guides explored adolescents’ attitudes, perceptions, preferences, and barriers to SRH service utilization. Tools were prepared in English, translated into Lusoga, and pilot-tested before data collection. Pilot testing supported the credibility of the tools by ensuring the clarity, cultural appropriateness, and relevance of the questions to the study context [19].

Four FGDs, each comprising 9–10 participants, were conducted with adolescents. Discussions were held in Lusoga, in neutral community venues and lasted 60–90 minutes. Each session was facilitated by a trained moderator of the same sex as participants, where possible, and supported by a note-taker. No external observers were present. Moderation followed a semi-structured guide, with probing and neutral phrasing used to manage sensitive topics and encourage participation. Prolonged engagement during discussions and the use of probing techniques enhanced credibility by allowing participants to express detailed and in-depth perspectives.

A total of 12 KIIs were conducted with health providers and community leaders. Interviews were conducted in either English or Lusoga, depending on participant preference. The inclusion of multiple participant categories enabled triangulation of perspectives, further strengthening the credibility of the findings [19].

All participants were approached through peer mobilization or community intermediaries, provided with study information, and invited to participate voluntarily. A small number of adolescents declined participation due to time constraints or discomfort discussing SRH in group settings, while no refusals were recorded among key informants. All FGDs and KIIs were audio-recorded with consent, and detailed field notes were taken to complement the recordings. The combination of audio recordings and field notes supported confirmability by ensuring that findings were grounded in participants’ accounts.

### Data analysis

Data collection and analysis proceeded iteratively, with emerging insights informing subsequent interviews. Saturation was assessed through regular team debriefs and concurrent review of transcripts, and data collection ceased at four FGDs and 12 KIIs when no new themes emerged, categories were well developed, and additional interviews were unlikely to yield meaningful insights. Regular debriefings also contributed to dependability by providing opportunities to review and refine emerging interpretations systematically [20].

Audio recordings were transcribed verbatim and, where necessary, translated into English. Back-translation was undertaken for selected transcripts to ensure accuracy and preserve meaning. All data were anonymized and stored on password-protected devices accessible only to the research team, in line with ethical requirements. Back-translation and careful data management procedures enhanced credibility and confirmability by preserving the authenticity and integrity of the data.

Data were analyzed inductively using Braun and Clarke’s reflexive thematic analysis, following six phases: familiarization with the data, coding, generating initial themes, reviewing themes, defining and naming themes, and producing the report [21]. Coding focused on participants’ accounts and meanings rather than pre-defined categories. Two researchers engaged independently with the data during the coding process and subsequently discussed interpretations to refine codes and themes through reflexive dialogue. NVivo version 14 software was used to support data organization and analytic transparency. Independent coding and collaborative theme refinement strengthened dependability by enhancing consistency in the analytic process, while the use of NVivo supported transparency and confirmability of the findings [21].

The research team comprised public health and social science researchers with prior experience in adolescent SRH research in Uganda. While the researchers did not have pre-existing relationships with participants, their professional backgrounds and familiarity with SRH programming informed analytic interpretation. Reflexive journaling was used throughout data collection and analysis to document assumptions, positionality, and evolving interpretations, and to minimize undue influence on coding and theme development. Reflexive practices further supported confirmability by ensuring that interpretations remained grounded in the data rather than researcher predispositions [19]. In addition, a comprehensive audit trail was maintained, including transcripts, field notes, coding frameworks, and analytic decisions, to enhance transparency and dependability of the research process.

Analysis was guided by the Health Belief Model [16] and Andersen’s Behavioral Model of Health Services Use [17], which were applied analytically to inform data interpretation. Emerging codes and themes were examined in relation to model constructs, including perceived susceptibility, perceived barriers, enabling resources, and sociocultural predisposing factors. This analytic application supported the interpretation of how adolescents’ motivations to protect their SRH interacted with structural and contextual constraints influencing service utilization.

### Ethical considerations

The study was approved by the Mildmay Uganda Research Ethics Committee (MUREC-2024–451) and registered by the Uganda National Council for Science and Technology (SS3374ES). Written informed consent was secured from all adult participants and from parents/guardians of minors, with adolescents’ assent. Participation was voluntary, and confidentiality and anonymity were maintained. Adolescents in FGDs received modest reimbursement for transport and time. Key informants received no compensation, as interviews were conducted at their workplaces or other convenient locations at no cost. We followed the consolidated criteria for reporting qualitative studies (COREQ) in reporting the findings [22].

## Results

### Characteristics of the study participants

A total of four FGDs were conducted across Bugweri and Iganga districts, stratified by sex and residence. The composition of the focus group discussions is shown in Table 1.

**Table 1:** Composition of focus group discussions (FGDs) by site.

FGD No.	Gender	Residence/Site	Mean Age	Schooling Status	No. of Participants
1	Female	Bugweri Urban	16.0	Mostly in	9
2	Female	Iganga Rural	15.2	Mostly in	9
3	Male	Iganga Urban	18.0	Mostly in	10
4	Male	Bugweri Rural	17.9	Mostly out	10

Male participants were drawn from rural Bugweri and urban Iganga, while female participants were recruited from urban Bugweri and rural Iganga. The mean age ranged from 15.2 to 18.0 years. Participants in rural Bugweri were predominantly out of school, whereas those in other sites were mostly in school. Each FGD comprised 9–10 participants.

A total of 12 KIIs were conducted with service providers, religious leaders, and cultural leaders across the two districts.

Table 2 shows the composition of the 12 KIIs conducted with service providers, religious leaders, and cultural leaders.

**Table 2:** Composition of key informant interviews (KIIs).

Participant Group	Gender	District	Rural (n)	Urban (n)	Participants per KII	Total Participants
Service providers	Female	Iganga	1	1	1	2
		Bugweri	1	1	1	2
Religious leaders	Male	Iganga	1	1	1	2
		Bugweri	1	1	1	2
Cultural leaders	Male	Iganga	1	1	1	2
		Bugweri	1	1	1	2
**Total**	—	—	**6**	**6**	**1**	**12**

Service providers were all female, while religious and cultural leaders were male. Each stakeholder group was equally represented across rural and urban settings (n = 6 each).

Table 3 summarizes the three main themes and sub-themes that emerged from the qualitative analysis.

**Table 3:** Overview of main themes and sub-themes on adolescent SRH service utilization.

Theme	Description	Key sub-themes
1. Positive orientation toward SRH prevention amid sexual self-discovery	Adolescents demonstrated proactive health-seeking behavior driven by perceived risks of pregnancy and STIs, often navigating SRH decisions independently due to limited parental guidance.	Perceived susceptibility to pregnancy and STIs; Gendered motivations for prevention; Self-directed learning and limited parental communication
2. Barriers to SRH service utilization	Multiple interrelated barriers at cognitive, structural, interpersonal, and sociocultural levels limited adolescents’ access to and utilization of SRH services.	Misconceptions and fears surrounding contraception; Structural, interpersonal, and sociocultural barriers (distance, privacy, provider attitudes, stigma); Sociocultural and system-level constraints (norms, early marriage, stockouts)
3. Preferences for adolescent-friendly and community-based services	Adolescents preferred decentralized, confidential, and youth-friendly service models delivered through community, school, and peer-led approaches to improve accessibility and trust.	Community- and school-based service delivery; Peer-led approaches and trust in information from fellow youth; Outreach and integration with existing community structures

The qualitative analysis generated three main themes reflecting adolescents’ lived experiences with SRH services, together with perspectives from health providers, religious, and cultural leaders (Table 3). These themes are analytically interconnected and demonstrate a clear progression from individual-level motivation to broader contextual constraints. Adolescents showed a proactive orientation and agency in seeking SRH services to protect themselves from unintended pregnancy and STIs (Theme 1). However, their efforts were significantly limited by multiple barriers operating at cognitive, structural, interpersonal, sociocultural, and system levels (Theme 2). In response, adolescents expressed clear preferences for decentralized, confidential, and peer-supported adolescent-friendly services (Theme 3).

### Detailed thematic findings

#### 1. Positive orientation toward SRH prevention amid sexual self-discovery.

Adolescents demonstrated a proactive orientation toward seeking SRH services, driven by a clear awareness of the risks associated with early sexual activity, including unintended pregnancies and STIs such as HIV. Many participants linked SRH services to protecting their future aspirations, particularly continuing education and avoiding early marriage.

Female adolescents frequently emphasized the protective role of SRH services. One participant explained:

*“It can help prevent early marriages. Some girls get pregnant, and then the family forces them to marry the boy. But if you go for SRH services, you get advice early, and you can avoid that kind of situation before it even starts.” (Female adolescent, FGD 2, rural Iganga)*

Another girl highlighted the importance of knowledge and personal agency

*“When we go to health workers, they educate us that if you don’t have money, you can ask your parents. If they don’t have it, you should be patient because not everything comes immediately. Adolescents need to be sharp. Even if a friend influences you, you don’t just follow. A knowledgeable girl can then positively influence others.” (Female adolescent, FGD 1, urban Bugweri)*

Male adolescents similarly recognized these benefits, often stressing personal responsibility and focus on school. One boy stated:

*“To prevent unintended pregnancies and infections like HIV. If you start having sex without knowing what you’re doing, you can easily get a girl pregnant or catch diseases. Seeking these services teaches you how to protect yourself and stay safe so you don’t carry heavy burdens while still young.” (Male adolescent, FGD 3, urban Iganga)*

Another male participant highlighted the practical value of services available locally:

*“It helps you prevent contracting STIs like HIV, syphilis, and others. You get health education at places such as the health center, and they also teach you about using condoms properly. That way, you protect yourself instead of just risking everything.” (Male adolescent, FGD 4, rural Bugweri)*

This positive orientation was often connected to broader life goals, with participants describing how access to SRH services reduced worries about pregnancy or infection and supported better school performance. However, this sense of agency developed amid limited parental guidance. Both health providers and religious leaders highlighted significant gaps in parent–child communication regarding sexual health.

One health provider explained the situation clearly

*“First of all, what I know is that parents lost interest in their children, so the children are growing themselves by self-discovery to everything.” (Health provider, KII, rural Iganga)*

A religious leader echoed this concern about parental negligence

*“It is due to the negligence of the parents, as they do not guide them on when to start sexual issues until they realize their children are seeking services in health facilities.” (Religious leader, KII, urban Bugweri)*

A cultural leader similarly noted that many parents have stopped advising their children:

*“Parents have stopped advising their children about bad conduct. We leaders must remind them. Health workers should invite us to community meetings so we can share our cultural values with the people.” (Cultural leader, KII, rural Iganga)*

In response, adolescents engaged in self-directed learning and independent health-seeking behaviors. This reflects a process of sexual self-discovery shaped by curiosity, peer influence, and perceived risk. However, this emerging agency was often constrained by incomplete or inaccurate information, particularly regarding contraception, which shaped their subsequent attitudes and decision-making.

Adolescents, therefore, turned to health services as an important source of information and support to help them navigate their developing sexuality and prevent negative outcomes.

#### 2. Barriers to SRH service utilization.

Adolescents faced multiple, interrelated barriers that limited their access to and utilization of SRH services. These barriers operated at cognitive, structural, interpersonal, and sociocultural levels.

***Misconceptions and fears surrounding contraception:*** Despite strong motivation to prevent unintended pregnancy, many adolescents held widespread misconceptions about modern contraceptive methods. Both female and male participants expressed deep fears that contraceptive use could cause long-term reproductive harm, particularly infertility:

*“Sometimes, I hear that family planning methods may cause you to become barren, and that scares me.” (Female adolescent, FGD 2, rural Iganga)*

A male participant echoed this concern:

*“Use of family planning by a woman who has never had children makes her sterile.” (Male adolescent, FGD 3, urban Iganga)*

Adolescents also shared additional misinformation about method safety and use:

*“We are told they affect women. They can get stuck in women, and that the lubricant in condoms is dangerous.” (Male adolescent, FGD 3, urban Iganga)*

Health providers confirmed the persistence of such misconceptions, noting that adolescents frequently worried about serious health consequences:

*“Some fear that if they use family planning methods, they will never get pregnant when they grow up. Others believe family planning methods cause cancer, that they can lose the uterus, or get fibroids. Because of these beliefs, some refuse to use family planning.” (Health provider, KII, urban Bugweri)*

*“And another thing which we always hear from them is that family planning causes cancer.” (Health provider, KII, urban Iganga)*

Religious perspectives further shaped normative attitudes toward contraception. A religious leader explained:

*“Some Muslim scholars also permit the use of condoms, but about the use of contraceptives, Islam does not recommend.” (Religious leader, KII, Bugweri)*

These fears of infertility, misinformation about health risks, and religiously influenced norms often outweighed the perceived benefits of contraception and discouraged uptake.

***Structural, interpersonal, and sociocultural barriers:*** Adolescents also encountered practical and relational obstacles when seeking services. Structural challenges, particularly long distances to facilities, high costs, and long waiting times, were frequently mentioned:

*“Government health facilities are far, and you can wait many hours before seeing a health worker.” (Male adolescent, FGD 3, urban Iganga)*

A female adolescent added:

*“For example, here, if you go at noon, you might not come back until 4 or 5 pm. So, I end up thinking, why don’t I just stay at home or go somewhere else… instead of wasting time at the health facility.” (Female adolescent, FGD 1, urban Bugweri)*

Privacy and confidentiality were major concerns. Participants noted that small or overcrowded health centers made confidential counseling difficult. A health provider confirmed this challenge:

*“The place is not enough for the adolescents to acquire knowledge or counseling because an adolescent needs privacy. But according to this health center, as small as it is, we don’t have enough privacy for the adolescents.” (Health provider, KII, rural Iganga)*

Negative provider attitudes further discouraged service use. Adolescents described experiences of being judged, spoken to harshly, or belittled by health workers. One girl recounted:

*“… the health workers come late, and they are arrogant and proud… you can even go back home without being attended to.” (Female adolescent, FGD 2, rural Iganga)*

Community leaders placed these experiences within broader social contexts of stigma and power imbalances. A cultural leader observed:

*“They are despised, which in turn makes them feel inferior. This affects their ability to ask for what they want. This happens a lot at health facilities.” (Cultural leader, KII, Bugweri)*

A religious leader added that shyness and fear related to their young age also prevented many adolescents from seeking care:

*“They are shy. They fear going to health facilities because they are young.” (Religious leader, KII, Bugweri)*

***Sociocultural and system-level constraints:*** Sociocultural norms and systemic challenges further shaped adolescents’ access to SRH services. Religious and cultural leaders emphasized norms around morality, sexuality, and marriage that often influenced when and how adolescents could engage with services:

*“Religion can guide good conduct, but sometimes we marry off adolescents early, and they miss opportunities.” (Religious leader, KII, Bugweri)*

*“Girls should provide for the god of the home before they get married...they should respect their ancestral spirits who are their spiritual husbands.” (Cultural leader, KII, Bugweri)*

At the same time, health system constraints restricted access to services. Providers highlighted persistent challenges related to the availability of commodities:

*“Sometimes it is stockouts, especially for family planning commodities. If one comes and does not find the method they want, they don’t come back.” (Health provider, KII, Bugweri)*

Adolescents similarly recognized these limitations and expressed the need for improved service readiness:

*“They should stock health facilities with more drugs…” (Female adolescent, FGD 2, Iganga)*

Institutional religious practices were also identified as structural barriers. One religious leader noted:

*“Rigidity of church procedures, such as testing for HIV first before marriage, is another challenge.” (Religious leader, KII, Iganga)*

Overall, cognitive, structural, interpersonal, sociocultural, and system-level barriers interacted to constrain adolescents’ utilization of SRH services.

#### 3. Preferences for adolescent-friendly and community-based services.

In response to the multiple barriers described above, adolescents expressed strong preferences for SRH services that were accessible, confidential, and tailored to their specific needs. Many emphasized the importance of decentralized delivery through community- and school-based platforms:

*“They should be everywhere—community, school and facilities.” (Male adolescent, FGD 3, urban Iganga)*

*“They should come up with clubs and specific days of sensitization.” (Male adolescent, FGD 4, rural Bugweri)*

Peer-led approaches were particularly valued. A female adolescent stated:

*“During school holidays, services should come to the community and be led by youth because we believe our peers.” (Female adolescent, FGD 2, rural Iganga)*

Health providers described ongoing efforts to reach adolescents through outreach activities, school visits, and collaboration with village health teams (VHTs):

*“We always provide health education when we go for outreaches, when we go for school health education, and also when we talk with the VHTs, tell them to inform them about the importance of our services at the health unit.” (Health provider, KII, Iganga)*

Community and religious leaders reported integrating adolescent guidance into existing social and institutional structures, including religious gatherings and personalized counseling:

*“We conduct community meetings. We preach and guide on good conduct during weekly prayers on Fridays.” (Religious leader, KII, Bugweri)*

*“We provide one-on-one guidance for adolescents on what should be done.” (Cultural leader, KII, Bugweri)*

These findings show both convergence and variation in how adolescent-friendly SRH services are conceptualized across stakeholders. Adolescents emphasized accessibility, confidentiality, and peer-based engagement, while providers focused on outreach-oriented service delivery, and community and religious leaders underscored moral guidance and behavioral regulation. Across these perspectives, common barriers, including contraceptive misinformation, limited privacy, long distances to facilities, negative provider attitudes, and sociocultural expectations, were consistently reported as shaping adolescents’ experiences with SRH services.

## Discussion

This study demonstrates that adolescents in the Busoga Region are motivated to protect their SRH, yet this motivation is frequently constrained by intersecting sociocultural and structural barriers. Analytically applying the HBM [16] and Andersen’s Behavioral Model [17] illustrates how strong perceived susceptibility to pregnancy and infection coexists with substantial perceived barriers, limited enabling resources, and restrictive community norms, resulting in delayed or foregone service utilization.

Consistent with previous studies from Uganda and other East African settings, misconceptions about contraception, stigma, confidentiality concerns, and unfriendly provider attitudes emerged as major deterrents to service use [3–9,14,15]. These findings resonate strongly with broader evidence across SSA. A qualitative systematic review synthesizing studies from multiple SSA countries identified similar multi-level barriers, including misconceptions about SRH services, fear of stigma from family and community, low self-esteem, financial constraints, unsupportive family communication, judgmental provider attitudes, lack of privacy, and commodity shortages [4]. In Ethiopia, adolescents similarly reported fear of being seen by family or community members, restrictive social and gender norms, and inadequate integration of SRH information in health facilities as key obstacles [6]. Studies in Nigeria and Ghana have highlighted individual-level barriers such as inadequate knowledge and poor attitudes toward services, compounded by strong social and religious norms that constrain open engagement with SRH issues [23,24].

Adolescents’ fears regarding infertility associated with contraceptive use mirror findings from across SSA and other low- and middle-income settings, where myths about long-term health complications (including infertility, cancer, or sterility) continue to shape SRH decision-making and deter uptake, particularly among nulliparous young people [4–6,9]. Such concerns are often reinforced by community, religious, and peer networks.

The triangulation of perspectives in this study (from adolescents, community/religious leaders, and health providers) highlights that these barriers are reinforced at multiple levels (individual, familial, community, and health system) rather than arising solely from adolescents’ knowledge gaps [4,25]. This multi-stakeholder lens complements earlier single-perspective or facility-focused studies and aligns with socio-ecological approaches used in other SSA contexts [4,15].

The findings can be interpreted through the lens of the HBM, which posits that health-related actions are shaped by perceived susceptibility, severity, benefits, barriers, cues to action, and self-efficacy [16]. In this study, strong barriers (particularly stigma and fear of judgment from providers or the community) often outweighed perceived benefits of early service use, such as better symptom control or improved long-term outcomes. As the HBM predicts, when barriers exceed benefits, individuals tend to delay or avoid care. Comparable applications of the HBM in adolescent SRH research in Ethiopia and Ghana have shown that perceived social risks, including shame and fear of disclosure, frequently outweigh health benefits, leading to delayed utilization [6].

Andersen’s Behavioral Model complements this by distinguishing predisposing, enabling, and need factors [17]. Predisposing elements, such as sociocultural norms around gender or community attitudes toward the condition, appeared to discourage help-seeking from the outset. Concurrently, limited enabling resources (for example, money problems, transport issues, or scarce local services) hindered action even when the need was recognized. This combined perspective reveals how individual perceptions (HBM) interact with structural obstacles (Andersen’s model) to explain the low service utilization observed, consistent with patterns in Tanzania and other low and middle-income countries (LMICs) where distance, cost, and service availability interact with sociocultural norms [26].

Importantly, evidence from SSA indicates that integrated, multi-component interventions (combining youth-friendly service provision, community engagement, and school-based programs) are more effective than single-component strategies for improving adolescent SRH outcomes [27,28]. Such approaches address both psychological barriers through anti-stigma efforts and improved communication, and practical access issues through enhanced resources, privacy, and service availability.

Overall, the present findings underscore shared structural and sociocultural challenges to adolescent SRH service utilization across diverse settings while highlighting the value of context-specific, theory-informed interventions in the Busoga Region and similar East African contexts.

### Implications for policy and practice

The results underscore the need for multi-component interventions that extend beyond facility-based service provision. At the health system level, there is a need to strengthen adolescent-friendly service delivery through targeted training of health providers in non-judgmental communication, confidentiality, and youth-responsive care, alongside the integration of adolescent SRH competencies into routine in-service training and supervision frameworks. Health facilities should also be supported to establish dedicated adolescent service corners, flexible service hours, and privacy-enhancing infrastructure to improve acceptability and utilization.

Expanding community- and school-based outreach programs, including the use of trained peer educators and collaboration with VHTs, can improve accessibility and trust in SRH services. In addition, structured community engagement strategies are needed to involve parents, religious leaders, and cultural gatekeepers in addressing misconceptions about contraception and promoting supportive norms around adolescent SRH. This may include dialogue forums, faith-based sensitization initiatives, and parent–adolescent communication programs.

To address persistent system barriers, policymakers should prioritize strengthening supply chain management systems to ensure consistent availability of contraceptives and essential SRH commodities, particularly in rural facilities. Finally, integrating adolescent SRH indicators into routine monitoring systems and strengthening accountability mechanisms can support the sustained delivery of responsive and equitable services.

### Strengths and limitations

A key strength of this study is the triangulation of perspectives from adolescents, community leaders, and health providers, which enhances credibility and contextual depth. However, the study was limited to two districts within the Busoga Region, potentially limiting transferability. While the findings offer insights that may be relevant to other similar low-resource settings with comparable sociocultural norms, economic challenges, and health system constraints, they are primarily grounded in the specific context of the Busoga region. Social desirability bias and challenges related to translation may also have influenced findings.

## Conclusion

This qualitative study demonstrates that adolescents in the Busoga Region of eastern Uganda have the motivation to protect their SRH, but face persistent sociocultural, structural, and health system barriers that constrain service utilization. Addressing misinformation, stigma, confidentiality concerns, and negative provider attitudes, alongside expanding adolescent-friendly and community-based service delivery, remains essential for improving adolescent SRH outcomes. In particular, decentralization of SRH services through community- and school-based platforms, outreach programs, and peer-led approaches represents a critical strategy for overcoming the distance, access, and confidentiality barriers identified in this study. Such approaches, however, warrant careful adaptation to local contexts rather than direct replication.
