# Supplementary material for: Stakeholder perspectives on adolescent sexual and reproductive health service utilization in Uganda’s Busoga region: A qualitative study
Source: PLoS One. 2026 Oct 1;21(10):e0358996. doi: 10.1371/journal.pone.0358996 (PMC13630231; doi:10.1371/journal.pone.0358996)
Supplement: Supplementary file 1 [file pone.0358996.s001.docx]

**Manuscript ID:** PONE-D-26-00996
**Title:** *Stakeholder perspectives on adolescent sexual and reproductive health service utilisation in Uganda’s Busoga region: A qualitative study*
**Journal:** PLOS ONE

**General Assessment**

This manuscript explores stakeholder perspectives on adolescent sexual and reproductive health (ASRH) service utilisation in the Busoga region of Uganda using qualitative research methods. The topic is highly relevant given the persistent barriers adolescents face in accessing sexual and reproductive health services in sub-Saharan Africa. The study addresses an important public health issue and contributes to the understanding of sociocultural, health system, and community-level factors influencing ASRH service uptake. The manuscript is generally well structured; however, several methodological clarifications and improvements in reporting are necessary to enhance transparency, rigor, and reproducibility.

1. Lines 14 - 17: The abstract background is relevant but currently too general for PLOS ONE standards. It would benefit from **greater specificity, context, and a clearer lead-in to the study rationale**. In its current form, the statements about “high rates” and “structural barriers” are plausible but not sufficiently informative, and they do not clearly justify why the Busoga region and a stakeholder perspective are needed. I recommend that the authors revise the background to include key quantitative indicators to support the claims, specify what “SRH services” refers to (“suitable services” is vague), improve the Uganda context by naming key barriers more precisely (“socio-cultural norms, misconceptions, and structural barriers” is broad), justify why the Busoga region is an important setting, clarify the “stakeholder perspectives” framing (end the background with a gap/aim bridge).
2. Line 17, replace “suitable services” with “adolescent-friendly SRH services.
3. Lines 19 – 22. In its current form, the objective combines several aims (exploring perspectives, identifying barriers and facilitators, and proposing strategies), which may dilute the clarity of the primary research purpose. For clarity and consistency with standards, the objective should more directly reflect the knowledge gap identified in the background and focus on the study's primary aim.
4. Lines 24 – 30: The methods described in the abstract provide a basic overview of the qualitative design; however, the section remains incomplete and does not fully meet the reporting clarity expected for abstracts. Suggestions for improvement include the study setting, specifying whether adolescents were recruited through schools, communities, health facilities, or youth groups, and include a brief statement indicating ethical approval and consent procedures.
5. Lines 32 – 38: The results section currently enumerates five themes without clearly articulating how these themes collectively explain adolescent SRH service utilisation in the study setting. Abstract results should briefly highlight the main analytical insight rather than only listing categories.
6. For the data collection, in general, the authors should consider expanding the data collection/procedure section to include recruitment and sampling processes, saturation rationale, and data security. These additions will substantially strengthen methodological transparency and align the manuscript with expected qualitative reporting standards (e.g., COREQ-aligned reporting).
7. Lines 109 – 115: The section does not explain how adolescents and key informants were identified and approached. Please add: recruitment sources for adolescents (schools, community groups, health facilities, youth clubs, CHVs/VHTs, NGOs, etc.); inclusion/exclusion criteria (age range is stated, but also consider residency, school attendance, marital status, pregnancy history if relevant); how urban/rural residence was defined; for KIIs: criteria for selecting “religious leaders” and “cultural leaders” (which institutions? which roles?); and whether any participants declined participation and why (if known)
8. FGD procedures need more operational detail, such as approximate group size per FGD; composition rules (you stratified by sex/residence—good—but specify if groups were mixed ages or age-banded); whether homogeneity was used to support open discussion (e.g., separate groups for younger adolescents 13–15 vs older 16–19, if applicable); presence of note-takers and whether observers were in the room; language used per group and how moderation handled sensitive topics.
9. Data saturation and stopping criteria: The authors should clarify how the team determined that sufficient information power/saturation was reached for FGDs and KIIs; and whether additional interviews were considered and why data collection stopped at four FGDs and twelve KIIs.
10. Lines 146 -259 (Results): The results presented are generally coherent and plausible for a qualitative study, and the thematic structure appears logically derived from the data. The use of illustrative quotes from different stakeholder groups (adolescents, health providers, religious leaders, cultural leaders) strengthens credibility and demonstrates triangulation across perspectives. The five themes—adolescent motivation, contraceptive misconceptions, access barriers, service preferences, and sociocultural/system constraints—are conceptually consistent with existing literature on adolescent sexual and reproductive health in East Africa. However, although the findings appear internally consistent, several important reporting elements are missing or insufficiently developed. Addressing these issues would significantly strengthen the rigor, clarity, and transparency of the results section.

- Lack of participant characteristics table: The results mention diversity among adolescents (in-school/out-of-school, married/unmarried, rural/urban), but no detailed description of participants is provided. A table summarizing participant characteristics should be included. This is essential for transparency in qualitative research.
- Theme structure needs clearer analytic framing: Currently, the themes are presented mainly descriptively, but the analysis should more clearly explain how themes relate to each other. For example:Adolescents show agency and motivation (Theme 1); But face misinformation (Theme 2); And structural barriers (Theme 3); Which leads them to prefer alternative service models (Theme 4); Within broader sociocultural and system constraints (Theme 5). Explicitly describing this analytical pathway would strengthen the narrative.
- Quotes are not always clearly attributed: Quotes are helpful but sometimes lack sufficient detail. The recommended format: “Quote text.” (Female adolescent, FGD 1, rural Bugweri) instead of (Girl, FGD, Bugweri). This helps readers understand participant diversity.
- Limited differentiation between stakeholder perspectives: The analysis sometimes merges perspectives from adolescents, providers, and leaders without clearly distinguishing them. For example: Adolescents emphasize privacy and stigma; Providers highlight service capacity issues; Religious leaders stress moral norms. Clarifying these differences would strengthen the multi-stakeholder analysis.
- Missing reflexivity and analytical interpretation: The results are largely descriptive, but qualitative research should also include interpretive insight. For example, instead of only describing parental absence, the analysis could interpret how parental silence contributes to informal knowledge networks, and how this reinforces peer-driven health seeking. Interpretation can remain brief but should show analytical depth.
- Saturation is not discussed: The results section should briefly indicate whether data saturation was achieved, especially given 4 FGDs and 12 KIIs. A statement such as “no new themes emerged in the final interviews, suggesting thematic saturation.”
- Weak integration of systems and cultural factors: Theme 5 is important, but currently reads as fragmented examples. It could better highlight the interaction between religion, early marriage norms, and poverty, or the interaction between health system weaknesses and trust.

11. Lines 305 – 310: The current conclusion extends the implications to “similar low-resource settings.” While the findings may indeed be relevant to other contexts, qualitative studies are generally context-specific, and such broader generalizations should be made cautiously. The authors should emphasize that the findings are primarily applicable to the Busoga region of eastern Uganda, while noting potential relevance to similar contexts.
