# Supplementary material for: Stakeholder perspectives on adolescent sexual and reproductive health service utilization in Uganda’s Busoga region: A qualitative study
Source: PLoS One. 2026 Oct 1;21(10):e0358996. doi: 10.1371/journal.pone.0358996 (PMC13630231; doi:10.1371/journal.pone.0358996)
Supplement: Supplementary file 2 [file pone.0358996.s002.docx]

PLOS One

Stakeholder perspectives on adolescent sexual and reproductive health service utilisation in Uganda’s Busoga region: A qualitative study

--Manuscript Draft--

| Manuscript Number: | PONE-D-26-00996 |
| --- | --- |
| Article Type: | Research Article |
| Full Title: | Stakeholder perspectives on adolescent sexual and reproductive health service utilisation in Uganda’s Busoga region: A qualitative study |
| Short Title: | Stakeholder perspectives on adolescent SRH services in Busoga, Uganda |
| Corresponding Author: | Noah Nyende  Nkumba University  UGANDA |
| Keywords: | Adolescent sexual and reproductive health; sexual and reproductive health services; service utilisation; stakeholder perspectives; qualitative research; Busoga region;  Uganda |
| Abstract: | Background  Adolescents in low- and middle-income countries continue to experience high rates of unintended pregnancy, sexually transmitted infections, and unmet sexual and reproductive health (SRH) needs. In Uganda, these challenges are exacerbated by sociocultural norms, misconceptions, and structural barriers that restrict adolescents’ access to suitable services. Objective  This study explored the perspectives of adolescents, community leaders, and health providers on adolescent SRH service utilisation in the Busoga Region of Eastern Uganda, to identify barriers, facilitators, and contextually appropriate strategies for improving adolescent-friendly service delivery.  Methods  An exploratory qualitative study was conducted using focus group discussions (FGDs) and key informant interviews (KIIs). Thirty-nine adolescents aged 13–19 years participated in four FGDs stratified by sex and residence (urban/rural). Twelve key informants, including health providers, religious leaders, and cultural leaders, were purposively selected.Data were collected between November and December 2024 using semi-structured guides, audio-recorded, transcribed verbatim, and analysed inductively using Braun and Clarke’s thematic analysis framework with support from NVivo 14. Results  Five major themes emerged: (1) adolescents’ positive orientation toward SRH prevention amid sexual self-discovery; (2) persistent misconceptions and fears surrounding contraception; (3) access barriers related to distance, privacy, and provider attitudes; (4) strong preferences for free, confidential, peer-informed, and community-based SRH services; and (5) sociocultural and system-level constraints, including early marriage norms, religious prohibitions, poverty, and commodity stockouts. Adolescents demonstrated motivation to protect their SRH but encountered intersecting barriers that constrained service utilisation.  Conclusion  Adolescents in the Busoga Region are motivated to access SRH services but face multilayered sociocultural and structural constraints. Strengthening adolescent-friendly service environments, improving provider attitudes, addressing misinformation through community engagement, and expanding school- and community-based outreach may enhance equitable SRH service utilisation. |
| Order of Authors: | Noah Nyende |
|  | Justine Nanteza |
|  | Frank Kiyingi |
|  | Miph Musoke |
| Additional Information: |  |

| Question | Response |
| --- | --- |
| Financial Disclosure  Enter a financial disclosure statement that describes the sources of funding for the work included in this submission. Review the [submission guidelines](http://journals.plos.org/plosone/s/submission-guidelines#loc-financial-disclosure-statement) for detailed requirements. View published research articles from [PLOS ONE](https://journals.plos.org/plosone/) for specific examples.  This statement is required for submission and will appear in the published article if the submission is accepted. Please make sure it is accurate. | The author(s) received no specific funding for this work. |
| Funded studies Enter a statement with the following details: - Initials of the authors who received each award - Grant numbers awarded to each author - The full name of each funder - URL of each funder website - Did the sponsors or funders play any role in the study design, data collection and analysis, decision to publish, or preparation of the manuscript? |  |
| Did you receive funding for this work? |  |
| Competing Interests  Use the instructions below to enter a competing interest statement for this submission. On behalf of all authors, disclose any [competing interests](http://journals.plos.org/plosone/s/competing-interests) that could be perceived to bias this work—acknowledging all financial support and any other relevant financial or nonfinancial competing interests.  This statement is required for submission and will appear in the published article if the submission is accepted. Please make sure it is accurate and that any funding sources listed in your Funding Information later in the submission form are also | The authors have declared that no competing interests exist. |

| declared in your Financial Disclosure statement.  View published research articles from [PLOS ONE](http://journals.plos.org/plosone/) for specific examples. |  |
| --- | --- |
| NO authors have competing interests Enter: The authors have declared that no competing interests exist. Authors with competing interests Enter competing interest details beginning with this statement: I have read the journal's policy and the authors of this manuscript have the following competing interests: [insert competing interests here] |  |
| * typeset |  |
| Ethics Statement  Enter an ethics statement for this submission. This statement is required if the study involved:   - Human participants - Human specimens or tissue - Vertebrate animals or cephalopods - Vertebrate embryos or tissues - Field research   Write "N/A" if the submission does not require an ethics statement.  General guidance is provided below. Consult the [submission guidelines](http://journals.plos.org/plosone/s/submission-guidelines#loc-guidelines-for-specific-study-types) for detailed instructions. Make sure that all information entered here is included in the Methods section of the manuscript. | Ethical approval was obtained from the Mildmay Uganda Research Ethics Committee  (MUREC-2024-451) and the Uganda National Council for Science and Technology (SS3374ES). Written informed consent was obtained from all adult participants and from parents or guardians of minors, with assent from adolescents. Participation was voluntary, and confidentiality and anonymity were maintained throughout the study. |

Format for specific study types

Human Subject Research (involving human

participants and/or tissue)

Give the name of the institutional review

board or ethics committee that approved the

study

•

Include the approval number and/or a

statement indicating approval of this

research

•

Indicate the form of consent obtained

(

written/oral) or the reason that consent was

not obtained (e.g. the data were analyzed

anonymously)

•

Animal Research (involving vertebrate

animals, embryos or tissues)

Provide the name of the Institutional Animal

Care and Use Committee (IACUC) or other

relevant ethics board that reviewed the

study protocol, and indicate whether they

approved this research or granted a formal

waiver of ethical approval

•

Include an approval number if one was

obtained

•

If the study involved

non-human primates

,

add

additional details

about animal welfare

and steps taken to ameliorate suffering

•

If anesthesia, euthanasia, or any kind of

animal sacrifice is part of the study, include

briefly which substances and/or methods

were applied

•

Field Research

Include the following details if this study

involves the collection of plant, animal, or

other materials from a natural setting:

Field permit number

•

Name of the institution or relevant body that

granted permission

•

Data Availability

Authors are required to make all data

underlying the findings described fully

available, without restriction, and from the

time of publication. PLOS allows rare

exceptions to address legal and ethical

concerns. See the

[PLOS Data Polic](http://journals.plos.org/plosone/s/data-availability)

[y](http://journals.plos.org/plosone/s/data-availability)

and

[FA](http://journals.plos.org/plosone/s/data-availability#loc-faqs-for-data-policy)

[Q](http://journals.plos.org/plosone/s/data-availability#loc-faqs-for-data-policy)

for detailed information.

No - some restrictions will apply

| A Data Availability Statement describing where the data can be found is required at submission. Your answers to this question constitute the Data Availability Statement and will be published in the article, if accepted. |  |
| --- | --- |
| Important: Stating ‘data available on request from the author’ is not sufficient. If your data are only available upon request, select ‘No’ for the first question and explain your exceptional situation in the text box. |  |
| Do the authors confirm that all data underlying the findings described in their manuscript are fully available without restriction? |  |
| Describe where the data may be found in full sentences. If you are copying our sample text, replace any instances of XXX with the appropriate details.   - If the data are held or will be held in a public repository, include URLs, accession numbers or DOIs. If this information will only be available after acceptance, indicate this by ticking the box below. For example: All XXX files   are available from the XXX database (accession number(s) XXX, XXX.).   - If the data are all contained within the manuscript and/or Supporting Information files, enter the following: All relevant data are within the manuscript and its Supporting Information files. - If neither of these applies but you are able to provide details of access elsewhere, with or without limitations, please do so. For example:   Data cannot be shared publicly because of [XXX]. Data are available from the XXX Institutional Data Access / Ethics Committee (contact via XXX) for researchers who meet the criteria for access to confidential data.  The data underlying the results presented in the study are available from (include the name of the third party | Data cannot be shared publicly due to the sensitive nature of the data and the potential risk of participant identification. De-identified qualitative transcripts are available from the corresponding author upon reasonable request and subject to approval by the relevant ethics committees. |

| and contact information or URL).  • This text is appropriate if the data are owned by a third party and authors do not have permission to share the data.  * typeset |  |
| --- | --- |
| Additional data availability information: | Tick here if the URLs/accession numbers/DOIs will be available only after acceptance of the manuscript for publication so that we can ensure their inclusion before publication. |

Manuscript	[Click here to access/download;Manuscript;Manuscript_January](https://www2.cloud.editorialmanager.com/pone/download.aspx?id=43279124&guid=d60205cb-27e0-43f6-b1b9-3e233d4163ce&scheme=1)

[9-2026_Stakeholder Perspectives on ASRH.docx](https://www2.cloud.editorialmanager.com/pone/download.aspx?id=43279124&guid=d60205cb-27e0-43f6-b1b9-3e233d4163ce&scheme=1)

1 **Stakeholder perspectives on adolescent sexual and reproductive**
2 **health service utilisation in Uganda’s Busoga region: A qualitative**

# 3 study

4 Noah Nyende¹* [https://orcid.org/0009-0001-6342-4113,](https://orcid.org/0009-0001-6342-4113) Justine Nanteza², Frank Kiyingi¹, Miph
5 Musoke¹

## 6 Affiliations

7 ¹ Nkumba University, Entebbe, Uganda
8 ² Makerere University School of Public Health, Kampala, Uganda

## 9 Corresponding author

10 Noah Nyende
11 Email: rnnyende2002@yahoo.com

# 12

# Abstract

## 13 Background

14 Adolescents in low- and middle-income countries continue to experience high rates of
15 unintended pregnancy, sexually transmitted infections, and unmet sexual and reproductive health
16 (SRH) needs. In Uganda, these challenges are exacerbated by sociocultural norms,
17 misconceptions, and structural barriers that restrict adolescents’ access to suitable services.

## Objective

This study explored the perspectives of adolescents, community leaders, and health providers on adolescent SRH service utilisation in the Busoga Region of Eastern Uganda, to identify barriers, facilitators, and contextually appropriate strategies for improving adolescent-friendly service delivery.

## Methods

An exploratory qualitative study was conducted using focus group discussions (FGDs) and key informant interviews (KIIs). Thirty-nine adolescents aged 13–19 years participated in four FGDs stratified by sex and residence (urban/rural). Twelve key informants, including health providers, religious leaders, and cultural leaders, were purposively selected. Data were collected between November and December 2024 using semi-structured guides, audio-recorded, transcribed verbatim, and analysed inductively using Braun and Clarke’s thematic analysis framework with support from NVivo 14.

## Results

Five major themes emerged: (1) adolescents’ positive orientation toward SRH prevention amid sexual self-discovery; (2) persistent misconceptions and fears surrounding contraception; (3) access barriers related to distance, privacy, and provider attitudes; (4) strong preferences for free, confidential, peer-informed, and community-based SRH services; and (5) sociocultural and system-level constraints, including early marriage norms, religious prohibitions, poverty, and commodity stock-outs. Adolescents demonstrated motivation to protect their SRH but encountered intersecting barriers that constrained service utilisation.

## Conclusion

Adolescents in the Busoga Region are motivated to access SRH services but face multilayered sociocultural and structural constraints. Strengthening adolescent-friendly service environments, improving provider attitudes, addressing misinformation through community engagement, and expanding school- and community-based outreach may enhance equitable SRH service

utilisation.

# Introduction

Adolescence is a critical developmental stage marked by rapid physical, psychological, and social transitions that have lasting implications for sexual and reproductive health outcomes [1,2]. Globally, adolescents account for a disproportionate burden of unintended pregnancies, unsafe abortions, and sexually transmitted infections, particularly in low- and middle-income countries [2,4]. In sub-Saharan Africa (SSA), structural barriers such as stigma, misconceptions, and limited access contribute to low utilisation of SRH care, and persistently high rates of teenage pregnancy and HIV [3,4]. Kenyan and Ethiopian studies, for example, highlight transport costs, judgmental providers, and restrictive cultural norms as key barriers [4,5].

In Uganda, adolescent SRH indicators remain concerning. Early sexual debut, low contraceptive uptake, and high rates of adolescent pregnancy persist, especially in socioeconomically disadvantaged regions such as Busoga in Eastern Uganda [6,7,8]. Uganda’s adolescent pregnancy rate is 24%, HIV prevalence among 15–19-year-olds is 2.1%, and modern contraceptive use is only 15% [3]. The Busoga Region is particularly affected because of poverty, early marriage norms, and limited services [9]. Despite national adolescent health guidelines [10], utilisation remains low due to stigma, lack of privacy, and misinformation [8, 9]; yet, little is known about how multiple stakeholders, including adolescents, community leaders, and healthcare providers, perceive these barriers and potential solutions. This gap limits the design of locally responsive, adolescent-friendly interventions.

While existing research has generated important insights, most studies focus on adolescents alone or examine health system factors in isolation. There is limited qualitative evidence that triangulates the perspectives of adolescents, community leaders, and health providers within the same sociocultural context [11]. Such triangulation is essential for understanding how community norms, service delivery practices, and adolescent preferences interact to shape SRH service utilisation.

This study aimed to explore adolescents’ attitudes, perceptions, and preferences regarding SRH services in the Busoga Region of Uganda, alongside the perspectives of community leaders and health providers. By examining convergences and divergences across stakeholder groups, the study sought to identify multilevel barriers and facilitators that can inform the design of contextually appropriate, adolescent-friendly SRH interventions. The study was guided by the

Health Belief Model [12] and Andersen’s Behavioural Model [13] to understand how individual perceptions, enabling factors, and sociocultural contexts shape SRH service utilisation.

# Materials and Methods

## Study design

An exploratory qualitative design was employed, guided by a constructivist interpretivist paradigm. This approach was appropriate for examining how adolescents and key community stakeholders construct meaning around SRH service utilisation based on their lived experiences.

## Study setting

The study was conducted in Iganga and Bugweri districts in the Busoga Region of Eastern Uganda. The region is characterised by a mix of rural and urban communities, varying levels of health service availability, and strong cultural and religious influences on adolescent behaviour. Iganga District has relatively better health infrastructure, while Bugweri District is more resource-constrained. Community entry was facilitated through district health officials and local council leaders. The study was conducted between 20^th^ November and 18^th^ December, 2024, at six rural (Namirali, Kiwerere, Bulubandi, Naitandu B, Bunalwenyi C, Makandwa) and six urban (Kasokoso Central II/III, Nakavule Main, Busesa, Butende, Kagamba) sites in Iganga and Bugweri districts. The two districts were selected because they reflect contrasting health system contexts: Bugweri has limited resources and fragile health infrastructure, whereas Iganga is better resourced with stronger systems and staffing, ensuring representation of diverse conditions influencing adolescent SRH service access and utilisation in the region.

## Participants and sampling

Two categories of participants were included. Adolescents aged 13–19 years participated in four FGDs stratified by sex and residence (urban/rural). In total, thirty-nine adolescents were recruited using convenience sampling with support from community mobilisers and youth peer leaders. Twelve key informants were purposively selected based on their roles and knowledge of adolescent SRH, including health providers, religious leaders, and cultural leaders. Sampling followed established qualitative research guidance [14] and proceeded iteratively. Participant recruitment continued until further data no longer added new patterns of meaning, which occurred after 12 key informant interviews and four focus group discussions. Inclusion criteria were adolescents aged 13–19 years residing in the study areas and leaders/providers identified by their communities or workplaces as knowledgeable about adolescent SRH services. (See *Table 1* for participant characteristics.)

Table 1: Characteristics of Study Participants

| **Participant group** | **Number of participants**  **(number of**  **FGDs/KIIs)** | **Age range** | **Gender composition** | **Residence** | **Other characteristics** |
| --- | --- | --- | --- | --- | --- |
| Adolescents  (FGDs) | 39 participants  (4 FGDs) | 13–19 years | Girls: 2 FGDs (13–17 years); Boys:  2 FGDs (17–  19 years) | 2 rural FGDs; 2 urban  FGDs | Education ranged from primary to secondary level; livelihoods included schooling, farming, vocational training, and informal work; predominantly low to moderate |
|  |  |  |  |  | socioeconomic  status |
| Key informants  (KIIs) | 12 (12 KIIs) | Not applicable | Mixed | Rural and urban | 6 health providers (health centres II–IV and district hospital); 6 community leaders  (3 cultural, 3 religious) |

## Data collection

Semi-structured interview guides were developed based on prior literature and national adolescent SRH frameworks. Guides explored attitudes, perceptions, preferences, and barriers related to SRH service utilisation. The tools were prepared in English, translated into Lusoga, and pilot-tested before data collection. FGDs were conducted in neutral community venues to enhance privacy and lasted between 60 and 90 minutes. KIIs were conducted in English or Lusoga, depending on participant preference. All sessions were audio-recorded with consent, and detailed field notes were taken.

## Data analysis

Audio recordings were transcribed verbatim and, where necessary, translated into English. Backtranslation was undertaken for selected transcripts to ensure accuracy and preserve meaning.

Data were analysed inductively using Braun and Clarke’s reflexive thematic analysis approach, following the six analytic phases of familiarisation with the data, coding, generating initial themes, reviewing themes, defining and naming themes, and producing the report [15]. Coding focused on participants’ accounts and meanings rather than pre-defined categories. Two researchers engaged independently with the data during the coding process and subsequently discussed interpretations to refine codes and themes through reflexive dialogue. NVivo version 14 software was used to support data organisation and analytic transparency.

The research team comprised public health and social science researchers with prior experience in adolescent sexual and reproductive health research in Uganda. While the researchers did not have pre-existing relationships with participants, their professional backgrounds and familiarity with SRH programming informed analytic interpretation. Reflexive journaling was used throughout data collection and analysis to document assumptions, positionality, and evolving interpretations, and to minimise undue influence on coding and theme development.

Analysis was guided by the Health Belief Model [12] and Andersen’s Behavioural Model of Health Services Use [13], which were applied analytically to inform data interpretation. Emergent codes and themes were examined in relation to model constructs, including perceived susceptibility, perceived barriers, enabling resources, and sociocultural predisposing factors. This analytic application supported the interpretation of how adolescents’ motivations to protect their sexual and reproductive health interacted with structural and contextual constraints influencing service utilisation.

**Ethical considerations**

Ethical approval was obtained from the Mildmay Uganda Research Ethics Committee (MUREC-

2024-451) and the Uganda National Council for Science and Technology (SS3374ES). Written informed consent was obtained from all adult participants and from parents or guardians of minors, with assent from adolescents. Participation was voluntary, and confidentiality and anonymity were maintained throughout the study.

# Results

The results draw on perspectives from adolescent girls aged 13–17 years and boys aged 17–19 years residing in rural and urban communities of Iganga and Bugweri districts in the Busoga Region. Participants included in-school and out-of-school adolescents, as well as married and unmarried adolescents, reflecting diverse social positions and sexual and reproductive health needs. Health providers contributed perspectives based on direct service delivery to adolescents, while cultural and religious leaders provided insight into the broader sociocultural context shaping adolescent behaviour and health-seeking.

The qualitative analysis generated five interrelated themes reflecting adolescents’ lived experiences and the perspectives of community leaders and health providers, presented with selected illustrative quotes:

1. Positive orientation toward SRH prevention amid sexual self-discovery

Adolescents demonstrated a proactive approach to sexual and reproductive health (SRH), driven by clear recognition of the risks of early sexual activity, particularly unintended pregnancy and sexually transmitted infections, including HIV. In focus group discussions, participants emphasised the protective role of SRH services: *“It helps us to prevent sexually transmitted infections and to plan for the future.” (Girl, FGD, Bugweri).*

Health providers corroborated this observation, noting that adolescents actively sought preventive measures due to specific fears: *“Girls fear pregnancies and boys fear illnesses like HIV, so they look for condoms and counselling.” (Health provider, KII, Bugweri).*

This positive orientation toward prevention developed largely independently, in a context where parental guidance on sexual matters was perceived as limited or absent. As one health provider described: *“First of all, what I know is that parents lost interest in their children, so the children are growing themselves by self-discovery to each and everything.” (Health provider, KII, Iganga).*

A religious leader similarly attributed adolescents' engagement with health services to parental negligence: *“It is due to the negligence of the parents, as they do not guide them on when to start sexual issues until they realise their children are seeking services in health facilities.” (Religious leader, KII, Bugweri).*

Thus, amid perceived gaps in parental involvement, adolescents exercised agency through self-discovery and help-seeking to safeguard their health and future aspirations.

2. Persistent misconceptions and fears surrounding contraception

Despite the expressed interest in prevention, widespread misconceptions and negative beliefs about modern contraceptive methods persisted, often deterring adolescents from utilising sexual and reproductive health services. Participants frequently cited fears that contraception could cause infertility, cancer, or other long-term health complications: *“Sometimes family planning methods may cause you to become barren.” (Girl, FGD, Iganga).*

*“Use of family planning by a woman who has never had children makes her sterile.” (Boy, FGD, Iganga).*

*“And another thing which we always hear from them is that family planning causes cancer.” (Health provider, KII, Iganga).*

Specific concerns were also raised about condoms: *“We are told they affect women. They can get stuck in women, and that the lubricant in condoms is dangerous.” (Boy, FGD, Iganga).*

Religious perspectives further contributed to these negative views, with some faith leaders expressing disapproval of contraceptive use: *“Some Muslim scholars also permit the use of condoms. About the use of contraceptives, Islam does not recommend.” (Religious leader, KII, Bugweri).*

Health providers and community members noted that such myths were commonly reinforced within families and religious settings, framing contraception as morally inappropriate or risky, which in turn discouraged adolescent uptake of services.

3. Barriers related to distance, privacy, and provider attitudes

Despite adolescents' proactive interest in sexual and reproductive health services, multiple structural and interpersonal barriers hindered access. Distance and transport costs posed significant challenges, especially in rural areas: *“Government health facilities are far, and you can wait many hours before seeing a health worker.” (Boy, FGD, Iganga).*

Privacy concerns were frequently highlighted, with participants noting that small or crowded facilities offered limited confidentiality, increasing the risk of being seen or recognised by community members: *“The place is not enough for the adolescents to acquire knowledge or counselling because an adolescent needs privacy. But according to this health center, as small as it is, we don't have enough privacy for the adolescents.” (Health provider, KII, Iganga).*

Negative attitudes from health workers further deterred service use, with adolescents describing experiences of judgement, harsh treatment, or being made to feel inferior: *“The health workers are tough. Sometimes we fear to open up.” (Girl, FGD, Iganga).*

Community leaders also recognised that social stigma, internalised inferiority, and age-based power imbalances within communities and health facilities fostered shyness and fear of judgement, which prevented young people from seeking care: *“They are despised, which in turn makes them feel inferior. This affects their ability to ask for what they want. This happens a lot at health facilities.” (Cultural leader, KII, Bugweri).*

*“They are shy. They fear going to health facilities because they are young.” (Religious leader, KII, Bugweri).*

These combined geographical, infrastructural, and interpersonal barriers contributed to delayed care-seeking, reduced trust in health facilities, and reluctance to return for subsequent services.

4. Preferences for adolescent-friendly and community-based services

Adolescents consistently expressed a strong preference for sexual and reproductive health services that were free, confidential, accessible, and tailored to their needs, with a particular emphasis on delivery outside traditional health facilities, such as in communities, schools, or through outreach programmes.

Participants highlighted the value of community- and school-based approaches led by peers or adolescent-friendly providers: *“During school holidays, services should come to the community and be led by youth because we believe our peers.” (Girl, FGD, Iganga).*

*“They should be everywhere, community, school and facilities.” (Boy, FGD, Iganga).*

*“They should come up with clubs and specific days of sensitisation.” (Boy, FGD, Bugweri).*

Health providers described existing efforts to reach adolescents through outreaches, school visits, and collaboration with village health teams (VHTs): *“We always provide health education when we go for outreaches, when we go for school health education and also when we talk with the*

*VHTs, tell them toinform them about the importance of our services at the health unit.” (Health provider, KII, Iganga).*

Community and religious leaders also contributed by integrating guidance into existing structures, such as religious gatherings and one-on-one counselling: *“We conduct community meetings. We preach and guide on good conduct during weekly prayers on Fridays.” (Religious leader, KII, Bugweri).*

*“We provide one-on-one guidance for adolescents on what should be done.” (Cultural leader, KII, Bugweri).*

Overall, adolescents and stakeholders favoured decentralised, peer-involved, and community embedded models that reduced barriers related to distance, stigma, and discomfort, thereby increasing the likelihood of service uptake.

5. Sociocultural and system-level constraints

Sociocultural norms and system-level challenges significantly constrained adolescents' access to sexual and reproductive health (SRH) services. Religious and cultural expectations often promoted early marriage or imposed restrictions that limited open engagement with SRH issues:

*“Religion can guide good conduct, but sometimes we marry off adolescents early, and they miss opportunities.” (Religious leader, KII, Bugweri).*

*“Girls should provide for the god of the home before they get married...they should respect their ancestral spirits who are their spiritual husbands.” (Cultural leader, KII, Bugweri).*

Religious practices further complicated access through rigid procedures: “*Rigidity of church procedures, such as testing for HIV first before marriage, is another challenge.” (Religious leader, KII, Iganga).*

At the health system level, frequent shortages of commodities and inadequate staffing undermined service reliability and trust: *“They should stock health facilities with more drugs, and health workers are also few.” (Girl, FGD, Iganga).*

*“Sometimes it is stockouts, especially for family planning commodities. If one comes and does not find the method they want, they don’t come back.” (Health provider, KII, Bugweri).*

These intertwined sociocultural pressures and systemic deficiencies, ranging from normative expectations around marriage and spirituality to persistent resource gaps, collectively restricted adolescents' ability to exercise their agency in seeking and utilising SRH services fully.

# Discussion

This study demonstrates that adolescents in the Busoga Region are motivated to protect their sexual and reproductive health, yet this motivation is frequently constrained by intersecting sociocultural and structural barriers. Analytically applying the Health Belief Model [12] and Andersen’s Behavioural Model 13] illustrates how strong perceived susceptibility to pregnancy and infection coexists with substantial perceived barriers, limited enabling resources, and restrictive community norms, resulting in delayed or foregone service utilisation.

Consistent with previous studies from Uganda and other East African settings, misconceptions about contraception, stigma, confidentiality concerns, and unfriendly provider attitudes emerged as major deterrents to service use [5,6,7,8,9]. Adolescents’ fears regarding infertility associated with contraceptive use mirror findings from sub-Saharan Africa, where myths continue to shape SRH decision-making [4,5,6,9]. The triangulation of perspectives in this study highlights that these barriers are reinforced at multiple levels, including individual, familial, community, and health system, rather than arising solely from adolescents’ knowledge gaps [16].

The findings can be interpreted through the lens of the Health Belief Model (HBM), which explains health-related actions as shaped by factors such as perceived susceptibility, severity, benefits, barriers, cues to action, and self-efficacy [12]. In this study, participants often described strong barriers, especially stigma around the condition and fear of judgment from providers or their community. These concerns seemed to outweigh the perceived benefits of seeking help early, such as better symptom control or improved long-term outcomes. As the HBM predicts, when barriers feel greater than benefits, people tend to delay or avoid care altogether.

Andersen's Behavioural Model of Health Services Use adds a wider view by separating the influences on service use into predisposing, enabling, and need factors [13]. Predisposing elements, like sociocultural norms around gender or community attitudes toward the condition, appeared to discourage help-seeking from the start. At the same time, limited enabling resources (for example, money problems, transport issues, or scarce local services) made it harder to act, even when participants clearly recognised their need for care. Together, the two models show how personal perceptions (from the HBM) combine with structural obstacles (from Andersen's model) to explain the low rates of service use we observed.

This combined perspective highlights the complex reasons behind care-seeking decisions. It also strengthens the case for interventions that address both sides: reducing psychological barriers through anti-stigma efforts and improving practical access through better resources and service availability.

# Implications for policy and practice

The results underscore the need for multi-component interventions that extend beyond facilitybased service provision. Strengthening adolescent-friendly service environments, expanding community- and school-based outreach, engaging parents and faith leaders in addressing misinformation, improving provider communication skills, and ensuring reliable commodity supply are critical steps supported by both participants’ perspectives and existing evidence.

# Strengths and limitations

A key strength of this study is the triangulation of perspectives from adolescents, community leaders, and health providers, which enhances credibility and contextual depth. However, the study was limited to two districts within the Busoga Region, potentially limiting transferability.

Social desirability bias and challenges related to translation may also have influenced findings.

# Conclusion

This qualitative study demonstrates that adolescents in the Busoga Region possess motivation to protect their sexual and reproductive health but face persistent sociocultural, structural, and health system barriers that constrain service utilisation. Addressing misinformation, stigma, confidentiality concerns, and provider attitudes, while expanding adolescent-friendly and community-based service delivery, remains essential for improving adolescent SRH outcomes in similar low-resource settings.

# Data Availability Statement

De-identified qualitative transcripts are available from the corresponding author upon reasonable request and subject to approval by the relevant ethics committees, due to the sensitive nature of the data and the potential risk of participant identification.

# Acknowledgements

I am deeply grateful to the adolescents who participated in this study. I also appreciate the research team for their invaluable contribution. Lastly, to the District Health Offices of Iganga and Bugweri, for their support and facilitation of this research.

# References

1. World Health Organization. Adolescent and young adult health [Internet]. Geneva: World

Health Organization; 2024 [updated 2024 Nov 26; cited 2026 Jan 7]. Available from:

[https://www.who.int/news-room/fact-sheets/detail/adolescents-health-risks-and-solutions.](https://www.who.int/news-room/fact-sheets/detail/adolescents-health-risks-and-solutions)

2. United Nations Population Fund. Adolescent sexual and reproductive health programmes in humanitarian settings: an in-depth look at family planning services [Internet]. New York: UNFPA; 2015 [cited 2026 Jan 7]. Available from: [https://www.unfpa.org/resources/adolescent-sexual-and-reproductive-health-programshumanitarian-settings-0.](https://www.unfpa.org/resources/adolescent-sexual-and-reproductive-health-programs-humanitarian-settings-0)
3. Uganda Bureau of Statistics (UBOS) and ICF. Uganda Demographic and Health Survey

2022 [Internet]. Kampala: UBOS and ICF; 2023 [cited 2026 Jan 7]. Available from: [https://www.ubos.org/wp-content/uploads/publications/UDHS-2022-Report.pdf.](https://www.ubos.org/wp-content/uploads/publications/UDHS-2022-Report.pdf)

4. Sidemo NB, Kerbo AA, Gidebo KD, Wado YD. Socio-ecological analysis of barriers to access and utilization of adolescent sexual and reproductive health services in subSaharan Africa: a qualitative systematic review. Adolesc Health Med Ther. 2023;14:123-

40.

5. Mutea L, Ontiri S, Kadiri F, Michielsen K, Gichangi P. Access to information and use of adolescent sexual reproductive health services: Qualitative exploration of barriers and facilitators in Kisumu and Kakamega, Kenya. PLoS One. 2020;15(11):e0241985.
6. Atuyambe L, Kibira SPS, Bukenya J, Muhumuza C, Apolot RR, Mulenga D. Understanding sexual and reproductive health needs of adolescents: evidence from a formative evaluation in Wakiso district, Uganda. Reprod Health. 2015;12:35.
7. Akatukwasa C, Gumputu B, Amongin D, Atwine D, Mumbere N, Behumbiize P, et al. Integration of HIV-sexual reproductive health services for young people and the barriers at public health facilities in Mbarara Municipality, Southwestern Uganda: a qualitative assessment. Int J Reprod Med. 2019;2019:6725432.
8. Nalwadda G, Mirembe F, Byamugisha J, Faxelid E. Persistent high fertility in Uganda: young people recount obstacles and enabling factors to use of contraceptives. BMC Public Health. 2010;10:530.
9. Nabugoomu JB, Kabonesa C, Kemigisha E, et al. Barriers to adolescent sexual and reproductive health services in Uganda. *Reprod Health*. 2020;17:92. *(Exact DOI available on PubMed)*
10. Ministry of Health Uganda. Adolescent health policy guidelines and service standards [Internet]. Kampala: Ministry of Health Uganda; 2011 [cited 2026 Jan 7]. Available from: [https://library.health.go.ug/sexual-and-reproductive-health/adolescent-health/adolescenthealth-policy-guidelines-and-service.](https://library.health.go.ug/sexual-and-reproductive-health/adolescent-health/adolescent-health-policy-guidelines-and-service)
11. Jonas K, Crutzen R, van den Borne B, Sewpaul R, Reddy P. Healthcare workers' beliefs, motivations and behaviours affecting adequate provision of sexual and reproductive healthcare services to adolescents in Cape Town, South Africa: a qualitative study. BMC

Health Serv Res. 2018;18(1):109.

12. Rosenstock IM. Historical origins of the health belief model. Health Educ Monogr.

1974;2:328–35.

13. Andersen RM. Revisiting the behavioral model and access to medical care: does it matter? J Health Soc Behav. 1995;36:1–10.
14. Guest G, Bunce A, Johnson L. How many interviews are enough? Field Methods.

2006;18:59–82.

15. Braun V, Clarke V. Using thematic analysis in psychology. Qual Res Psychol.

2006;3:77–101.

16. Bronfenbrenner U. The ecology of human development. Cambridge (MA): Harvard

University Press; 1979.
