# Supplementary material for: Stakeholder perspectives on adolescent sexual and reproductive health service utilization in Uganda’s Busoga region: A qualitative study
Source: PLoS One. 2026 Oct 1;21(10):e0358996. doi: 10.1371/journal.pone.0358996 (PMC13630231; doi:10.1371/journal.pone.0358996)
Supplement: Supplementary file 3 [file pone.0358996.s004.docx]

May 28^th^ 2026

Dear Editor,

I appreciate the opportunity to review this manuscript. The authors have made significant and commendable efforts to address the reviewers’ comments and to substantially improve the manuscript's quality, clarity, and methodological reporting. The revised version demonstrates improved coherence, stronger analytical presentation, and better alignment with the reporting standards expected for qualitative research in PLOS ONE.

In my opinion, the major concerns raised during the review process have been adequately addressed, and the manuscript now makes a meaningful contribution to the understanding of adolescent sexual and reproductive health service utilization in low-resource settings.

I therefore recommend acceptance of the manuscript for publication in PLOS ONE.

Sincerely,

Reviewer
