# Supplementary material for: Stakeholder perspectives on adolescent sexual and reproductive health service utilization in Uganda’s Busoga region: A qualitative study
Source: PLoS One. 2026 Oct 1;21(10):e0358996. doi: 10.1371/journal.pone.0358996 (PMC13630231; doi:10.1371/journal.pone.0358996)
Supplement: Supplementary file 4 [file pone.0358996.s005.docx]

PLOS One

Stakeholder perspectives on adolescent sexual and reproductive health service utilisation in Uganda’s Busoga region: A qualitative study

--Manuscript Draft--

| Manuscript Number: | PONE-D-26-00996R1 |
| --- | --- |
| Article Type: | Research Article |
| Full Title: | Stakeholder perspectives on adolescent sexual and reproductive health service utilisation in Uganda’s Busoga region: A qualitative study |
| Short Title: | Stakeholder perspectives on adolescent SRH services in Busoga, Uganda |
| Corresponding Author: | Noah Nyende  Nkumba University  UGANDA |
| Keywords: | Adolescent sexual and reproductive health; sexual and reproductive health services; service utilisation; stakeholder perspectives; qualitative research; Busoga region;  Uganda |
| Abstract: | Background  Background  Adolescents in low- and middle-income countries face high rates of unintended pregnancy, sexually transmitted infections (STIs), and unmet sexual and reproductive health (SRH) needs. In Uganda, 24% of girls aged 15–19 years have begun childbearing, yet modern contraceptive prevalence among adolescents remains low (15%). These challenges are particularly severe in the Busoga region, where sociocultural norms, poverty, and limited health system capacity hinder access to adolescent-friendly SRH services. Despite national guidelines, utilization remains low. Few studies have triangulated perspectives from adolescents, health providers, and community/religious leaders. We explored stakeholder views to provide an integrated understanding of factors influencing adolescent SRH service utilization in Busoga, Uganda.  Methods  An exploratory qualitative study was conducted in Iganga and Bugweri districts. Four focus group discussions with 38 adolescents (13–19 years), stratified by sex, age group, and residence, and 12 key informant interviews with health providers and community leaders were conducted. Adolescents were recruited through peer networks, while key informants were purposively selected. Data were collected (November–December 2024), transcribed verbatim, and analyzed inductively using thematic analysis with NVivo 14. Ethical approval and informed consent/assent were obtained. Results  Adolescents, health providers, and community stakeholders described SRH service utilization as shaped by adolescent motivation alongside persistent barriers. Adolescents showed strong preventive intent, but uptake was limited by  misconceptions about contraception, fears of infertility, long distances, lack of privacy, negative provider attitudes, and commodity stock-outs. Providers and community leaders echoed concerns around misinformation, limited parental guidance, moral norms, and structural constraints. All stakeholders converged on the need for confidential, accessible, and peer- and community-based adolescent SRH services.  Conclusion  Adolescents in Busoga are motivated to use SRH services, but intersecting sociostructural and system barriers limit utilization. Strengthening adolescent-friendly services, improving provider responsiveness, addressing misinformation, and expanding community-based delivery are essential to enhance equitable access. |
| Order of Authors: | Noah Nyende |
|  | Justine Nanteza |
|  | Frank Kiyingi |
|  | Miph Musoke |

| Response to Reviewers: | Dear Editor and Reviewers,  We sincerely thank you for your thorough, rigorous, and constructive review of our manuscript. Your detailed feedback has been invaluable in significantly strengthening the clarity, methodological transparency, analytical depth, and overall quality of our work. We have carefully revised the manuscript in response to all comments. Below, we provide a detailed, point-by-point response, with corresponding line references to the revised manuscript. All major revisions and additions are highlighted in bold text for ease of review.  ________________________________________  1. PLOS ONE Formatting and Style  Comment: Ensure the manuscript complies with PLOS ONE formatting guidelines. Response: The manuscript has been fully revised to comply with PLOS ONE formatting requirements, including structure, headings, referencing style, and file naming conventions. The abstract now follows the structured format (Background, Methods, Results, Conclusion) as required.  ________________________________________  2–4. Data Availability and Open Data Policy  Comment: Clarify data sharing, restrictions, and repository deposition. (Reviewers #2,  #3, and #4 raised concerns about data availability.)  Response: We have substantially revised the data availability approach to align fully with PLOS ONE's open data policy:  •A fully de-identified qualitative dataset (focus group discussions and key informant interviews) has been prepared following established anonymization standards (e.g., BMJ 2010).  •All direct identifiers (names of participants, specific village names, health facilities, dates, and institutional affiliations) have been removed or generalized to protect participant confidentiality.  •The dataset has been deposited in a public repository .  •The Data Availability Statement has been updated to include the repository link and accession number.  •We confirm that written informed consent (and assent for minors) explicitly included permission for the use of anonymized data in research outputs and public repositories.  •No potentially identifiable information remains in the deposited dataset.  This ensures full compliance with PLOS ONE's requirement that data underlying findings be publicly accessible while preserving participant confidentiality.  Revised text: Data Availability Statement (Lines 487–490); Ethical Considerations (Lines 188–196).  ________________________________________  Point-by-Point Responses to Reviewer Comments  We have consolidated comments from all four reviewers and respond to each below. ________________________________________  Abstract  Comment 1 (Reviewers #1 and #2): The abstract background is too general. It needs greater specificity, quantitative indicators, and a clearer rationale for the Busoga region and stakeholder approach.  Response: The abstract background has been completely revised to include:  •Specific quantitative indicators: 24% adolescent childbearing rate, 15% modern contraceptive prevalence among adolescents (Uganda Demographic and Health Survey 2022).  •Named barriers: sociocultural norms, poverty, limited health system capacity. •Clear justification: the Busoga region is disproportionately affected by these challenges, yet no study has triangulated perspectives from adolescents, providers, and community/religious leaders.  Revised text: Abstract, Lines 13–20.  Comment 2 (Reviewer #1): Replace "suitable services" with a more precise term. Response: "Suitable services" has been replaced with "adolescent-friendly SRH services" throughout the abstract and manuscript.  Revised text: Abstract, Line 17; also consistent throughout.  Comment 3 (Reviewers #1 and #2): The objective combines several aims, diluting clarity. It should more directly reflect the primary research purpose.  Response: The objective has been refined to focus on the single primary aim: to explore stakeholder perspectives to provide an integrated understanding of factors influencing adolescent SRH service utilization. The secondary elements (barriers, facilitators, strategies) now appear in the results and conclusion. |
| --- | --- |

|  | Revised text: Abstract, Lines 19–20.  Comment 4 (Reviewer #1): The abstract methods section is incomplete.  Response: The methods section has been expanded to include:  •Study setting (Iganga and Bugweri districts, Busoga region).  •Recruitment approach: adolescents through peer networks; key informants purposively selected.  •A clear statement of ethical approval and consent procedures.  Revised text: Abstract, Lines 22–28.  Comment 5 (Reviewers #1, #2, and #3): The results section lists themes descriptively but does not articulate the main analytical insight. The abstract and results should reflect the collapse of five themes into three.  Response: Thank you for this critical observation. We have substantially restructured the results to address this concern.  First, the original five themes have been collapsed into three analytically coherent themes that follow a clear logical progression from motivation → barriers → solutions:  Original Five ThemesCollapsed into Three Themes   1. Positive orientation toward SRH prevention amid sexual self-discoveryTheme 1:   Positive orientation toward SRH prevention amid sexual self-discovery (motivation)   2. Persistent misconceptions and fears surrounding contraception↓ 3. Access barriers related to distance, privacy, and provider attitudesTheme 2: Barriersto SRH service utilization (barriers)   5. Sociocultural and system-level constraints↓  4. Strong preferences for free, confidential, peer-informed, and community-based SRH servicesTheme 3: Preferences for adolescent-friendly and community-based services  (solutions)  Second, the results section has been reframed to highlight the core analytical insight: adolescents are motivated to use SRH services, but this motivation is constrained by intersecting barriers, leading them to prefer alternative service models. The three themes are now presented within this analytical narrative rather than as a simple list. Third, Theme 3 (Preferences) is now clearly articulated and highlighted in bold throughout the results section. This theme captures adolescents' strong preferences for:  •Free, confidential, and accessible services  •Community- and school-based delivery platforms  •Peer-led approaches (because "we believe our peers")  •Outreach programmes and integration with existing community structures (e.g., village health teams, religious gatherings)  Revised text: Abstract, Lines 30–36; Results, Lines 216–224 (theme overview) and Lines 359–381 (Theme 3: Preferences for adolescent-friendly and community-based services).  ________________________________________  Methods (Data Collection, Recruitment, and Transparency)  Comment 6 (Reviewers #1, #2, and #3): Expand the data collection/procedure section to include recruitment and sampling processes, saturation rationale, and data security.  Response: The Methods section has been substantially expanded to include:  •Detailed recruitment processes for adolescents (via community mobilisers and youth peer leaders) and key informants (purposively selected based on institutional roles).  •Explicit saturation rationale: data collection continued until no new patterns emerged, achieved after 4 FGDs and 12 KIIs, confirmed through team debriefing and concurrent transcript review.  •Data security procedures: anonymization, password-protected devices, restricted access to the research team.  Revised text: Lines 99–162.  Comment 7 (Reviewers #1, #2, and #3): Explain how adolescents and key informants were identified and approached. Add inclusion/exclusion criteria, definitions of urban/rural residence, and whether any participants declined. Reviewer #2 specifically noted that the sampling strategy needed clarification, and Reviewer #3 noted that convenience sampling was incorrectly stated.  Response: This section has been significantly expanded. We have corrected the sampling description: it is purposive sampling, not convenience sampling.  •Adolescents were purposively sampled to capture diverse perspectives across gender, age, and residential contexts. Recruitment was facilitated through community mobilisers and youth peer leaders, with outreach in communities.  •Inclusion criteria for adolescents: age 13–19 years, residence in the study area for at |
| --- | --- |

|  | least six months, and willingness to participate. No exclusion criteria were applied beyond these.  •Key informants were purposively selected based on formal institutional roles (health providers: nurses and clinical officers; religious leaders: clergy, pastors, imams; cultural leaders: clan leaders and elders) and recognized community authority.  •A small number of adolescents declined participation due to time constraints or discomfort discussing SRH in group settings. No refusals were recorded among key informants.  Revised text: Lines 110–146.  Comment 8 (Reviewers #1 and #2): Provide more operational detail for FGD procedures: group size, composition rules, presence of note-takers, language, and handling of sensitive topics.  Response: The following details have been added:  •Each FGD comprised 9–10 participants.  •Groups were stratified by sex and residence. Age stratification was implicit: female groups were younger (13–17 years); male groups were older (17–19 years) to reflect developmental stages and facilitate open discussion.  •Each session was facilitated by a trained moderator of the same sex as participants. A note-taker was present; no external observers were in the room.  •All FGDs were conducted in Lusoga, the local language.  •Sensitive topics were managed using neutral phrasing, probing techniques, and reassurance of confidentiality. Participants were reminded that they could skip any question or withdraw at any time.  Revised text: Lines 132–138.  Comment 9 (Reviewers #1, #2, and #3): Clarify how the team determined that sufficient saturation was reached for FGDs and KIIs.  Response: A clear saturation statement has been added:  •Sampling proceeded iteratively, with regular team debriefings and concurrent review of transcripts.  •Saturation was considered reached when no new themes emerged, categories were well-developed, and additional interviews were unlikely to yield meaningful new insights.  •This occurred after 4 FGDs and 12 KIIs, at which point data collection ceased. Revised text: Lines 122–124 (sampling) and Lines 151–155 (analysis).  Comment 10 (Reviewer #4): Ensure trustworthiness parameters (credibility, dependability, confirmability, transferability) are explicitly stated within the context of the research. Cite relevant literature.  Response: Thank you for this important guidance. The Methods section has been revised to explicitly address all four trustworthiness criteria:  •Credibility: achieved through prolonged engagement during discussions, probing techniques, triangulation of multiple stakeholder perspectives, and back-translation of selected transcripts (Lines 132–138, 151–155).  •Dependability: ensured through regular team debriefings, independent coding by two researchers, and maintenance of a comprehensive audit trail (Lines 151–162).  •Confirmability: supported by reflexive journaling, documentation of assumptions and positionality, and grounding interpretations in participant accounts rather than researcher predispositions (Lines 162–168).  •Transferability: acknowledged that findings are primarily applicable to the Busoga region (Lines 470–471).  Revised text: Lines 132–138, 151–168, 470–471.  ________________________________________  Results Section  Comment 11a (Reviewers #1 and #2): No detailed description of participants is provided. A table summarizing participant characteristics is essential.  Response: Two new tables have been added:  •Table 1: Composition of Focus Group Discussions (FGDs) by site, gender, mean age, schooling status, and number of participants.  •Table 2: Composition of Key Informant Interviews (KIIs) by participant group, gender, district, rural/urban location, and total participants.  Revised text: Lines 201–212 (Tables 1 and 2).  Comment 11b (Reviewers #1 and #2 – Summary table of themes and sub-themes): Reviewer #1 specifically noted: "The authors may consider including a summary table of themes and subthemes to improve readability." Reviewer #2 similarly suggested organizing the findings with a table. |
| --- | --- |

|  | Response: Thank you for this practical suggestion. We have included Table 3 in the revised manuscript, which provides a clear, at-a-glance summary of the three main themes and their corresponding sub-themes.  This table directly addresses Reviewer #1's request for improved readability and Reviewer #2's suggestion for better organization of findings. It appears immediately after the participant characteristics tables (Tables 1 and 2) and before the detailed thematic narrative, allowing readers to orient themselves before reading the full results.  Revised text: Lines 214–224 (Table 3).  Comment 11c (Reviewers #1, #2, and #3): Themes are presented descriptively; the analysis should explain how themes relate to each other. The collapse of five themes into three should be clearly shown.  Response: The results section has been restructured to present three interconnected themes that follow a clear analytical progression:  1.Theme 1: Positive orientation toward SRH prevention amid sexual self-discovery (motivation and agency).  2.Theme 2: Barriers to SRH service utilization (sub-themes: misconceptions, structural/interpersonal barriers, sociocultural/system constraints).  3.Theme 3: Preferences for adolescent-friendly and community-based services (solutions).  An explicit analytical pathway has been added: adolescents show motivation (Theme 1) → but face barriers (Theme 2) → leading them to prefer alternative service models (Theme 3).  Within Theme 3, we now clearly articulate adolescents' specific preferences, including: free and confidential services; community- and school-based delivery platforms; peerled approaches ("because we believe our peers"); outreach programmes; and integration with existing community structures such as village health teams and religious gatherings.  Revised text: Lines 216–224 (overview), and throughout the Results section (Lines 233–381).  Comment 11d (Reviewers #1 and #2): Quotes lack sufficient detail. Use a standardized format.  Response: All quotes have been standardized to the following format: (Quote text, Participant group, FGD/KII number, district, rural/urban). For example: (Female adolescent, FGD 2, rural Iganga). This has been applied consistently throughout the Results section.  Revised text: Throughout Lines 233–381.  Comment 11e (Reviewer #3): The analysis sometimes merges perspectives without clearly distinguishing them. Provide clearer differentiation between stakeholder groups.  Response: The analysis has been revised to clearly distinguish perspectives:  •Adolescents: emphasize privacy, stigma, fear of judgment, and preferences for peerled services.  •Health providers: highlight service capacity issues, stockouts, staffing shortages, and efforts to reach adolescents through outreach.  •Religious and cultural leaders: stress moral norms, early marriage practices, religious prohibitions on contraception, and the role of community guidance.  This differentiation is now explicit throughout the Results section.  Revised text: Lines 233–381 (e.g., separate quotes and analysis for each stakeholder group within each theme).  Comment 11f (Reviewers #1 and #3): Results are largely descriptive; interpretive insight is needed. Add reflexivity and analytical interpretation.  Response: Interpretive commentary has been added throughout the Results section, including:  •Parental silence: how limited parent–adolescent communication drives self-directed learning and reliance on peer networks.  •Misinformation: how fears of infertility and cancer, reinforced by community and religious narratives, outweigh perceived benefits of contraception.  •Barrier interaction: how distance, privacy, provider attitudes, and stockouts combine to create compounded access failures.  •Preference logic: why adolescents prefer community-based and peer-led services as a direct response to stigma and confidentiality concerns (explicitly developed in Theme 3).  Revised text: Lines 233–381 (interpretive statements added within each theme). Comment 11g (Reviewers #1 and #2): The results section should briefly indicate whether saturation was achieved. |
| --- | --- |

|  | Response: A saturation statement has been added to the Results section:  Revised text: Lines 122-124, 151–155.  Comment 11h (Reviewer #3): The integration of systems and cultural factors (formerly Theme 5) reads as fragmented. Better highlight interactions.  Response: Theme 2 (which now incorporates the former Theme 5) has been substantially revised to show the interaction between religion, early marriage norms, poverty, and health system weaknesses. The sub-theme now explicitly demonstrates how:  •Religious norms and early marriage practices constrain adolescents' agency.  •Health system stockouts and staffing shortages compound these cultural barriers. •The combination of normative expectations and service gaps leads to delayed or foregone care.  Revised text: Lines 277–358 (previously Theme 5, now integrated into Theme 2). ________________________________________  Discussion  Comment 12 (Reviewers #2, #3, and #4): The discussion would benefit from broader comparison with studies from other sub-Saharan African countries and similar settings outside Africa. Reviewer #4 specifically noted that only one study from South Africa was cited.  Response: The discussion has been substantially expanded to include comparisons with studies from Ethiopia, Nigeria, Ghana, Kenya, Tanzania, and other sub-Saharan African countries. New references have been added (References 4, 5, 6, 23, 24, 26). The discussion now explicitly situates our findings within the broader SSA evidence base while highlighting the unique contribution of multi-stakeholder triangulation in the Busoga context.  Revised text: Lines 400–445.  Comment 13 (Reviewer #2): Some sections explaining the theoretical models are relatively long and could be more directly connected to the study findings.  Response: The theoretical application sections have been shortened and more tightly integrated with specific findings from each theme. Direct links between HBM constructs (perceived barriers, perceived benefits) and Andersen's model (predisposing, enabling, need factors) are now explicitly mapped to participant quotes.  Revised text: Lines 420–445.  ________________________________________  Implications for Policy and Practice  Comment 14 (Reviewer #2): Provide more concrete recommendations for health systems, such as training of providers, strengthening youth-friendly services, or community engagement strategies.  Response: The Implications section has been expanded to include specific, actionable recommendations:  •Health system level: targeted provider training in non-judgmental communication, dedicated adolescent service corners, flexible service hours, and privacy-enhancing infrastructure.  •Community level: structured engagement with parents, religious leaders, and cultural gatekeepers through dialogue forums and faith-based sensitization initiatives.  •School level: peer education programmes and integration of SRH information into existing curricula.  •Supply chain: strengthening commodity supply chain management to reduce stockouts, particularly for contraceptives in rural facilities.  Revised text: Lines 451–467.  ________________________________________  Strengths and Limitations  Comment 15 (Reviewer #2): The limitations section could also mention possible selection bias due to sampling and limitations in generalizability beyond the study region.  Response: The limitations section has been expanded to explicitly acknowledge:  •Geographic scope – limited to two districts in Busoga, limiting transferability  •Context specificity – findings primarily grounded in Busoga region  •Social desirability bias – participants may have given socially acceptable responses •Translation challenges – potential loss of nuance in translation from Lusoga to English Revised text: Lines 470–475.  ________________________________________  Conclusion  Comment 16 (Reviewers #1 and #2): The conclusion overgeneralizes findings to |
| --- | --- |

|  | "similar low-resource settings" without sufficient caution. Emphasize specific actionable recommendations.  Response: The conclusion has been revised to:  •Emphasize that the findings are primarily applicable to the Busoga region of eastern Uganda.  •Note cautiously that the insights may be relevant to other contexts with comparable sociocultural norms, economic challenges, and health system constraints, but that direct replication requires contextual adaptation.  •Include specific actionable recommendations: decentralisation through communityand school-based platforms, outreach programmes, peer-led approaches, and confidentiality-enhancing service design.  Revised text: Lines 477–485.  ________________________________________  Language and Grammar (Reviewer #4)  Reviewer ConcernAction Taken  Abstract too general (Reviewer #1)Added quantitative indicators (24%, 15%) and specific barriers  Objective overloaded (Reviewer #1)Refined to single primary aim  Methods incomplete (Reviewer #1)Added setting, recruitment, ethics  Results list themes descriptively (Reviewers #1, #2, #3)Collapsed 5 themes into 3; added analytical pathway  Summary table of themes and sub-themes (Reviewers #1 and #2)Added Table 3  (Lines 214–224)  Theme 3 (Preferences) not clearly articulatedNow highlighted in bold with specific preferences listed  Sampling strategy unclear; "convenience sampling" incorrect (Reviewers #2,  #3)Corrected to purposive sampling; added detailed recruitment  Saturation rationale missing (Reviewers #1, #2, #3)Added explicit saturation statement  Participant characteristics table missing (Reviewers #1, #2)Added Tables 1 and 2  Quotes lack attribution (Reviewers #1, #2)Standardized quote format  Stakeholder perspectives merged (Reviewer #3)Differentiated perspectives clearly Interpretive depth missing (Reviewers #1, #3)Added interpretive commentary throughout  Trustworthiness criteria incomplete (Reviewer #4)Added credibility, dependability, confirmability, transferability  Discussion too narrow (Reviewers #2, #3, #4)Expanded to include Ethiopia, Nigeria,  Ghana, Kenya, Tanzania  Language errors (Reviewer #4)Corrected Line 152, Line 283, and throughout  References inconsistent (Reviewers #2, #3, #4)Standardized formatting; added recent  SSA literature  Data availability restrictions (All reviewers)Deposited de-identified data in public repository; updated Data Availability Statement  Comment 17 (Reviewer #4): Minor grammatical errors need to be reviewed. Specific line edits:  •Line 121: rephrase to "... shaping adolescent health-seeking behaviour."  •Line 229: replace "like" with "such as" (and amend where applicable throughout the manuscript).  Response: These corrections have been made. Line 121 now reads "... shaping adolescent health-seeking behaviour." Line 229 and other instances of "like" have been changed to "such as" where appropriate. The entire manuscript has been carefully proofread for additional grammatical and typographical errors. Revised text: Lines 121, 229, and throughout.  ________________________________________  References  Comment 18 (Reviewers #2, #3, and #4): Ensure consistency of the referencing method. Include more recent literature and studies from other SSA countries.  Response: The reference list has been updated to ensure consistency in formatting (all now follow PLOS ONE style). New references from Ethiopia, Nigeria, Ghana, Kenya, and Tanzania have been added (References 4, 5, 6, 23, 24, 26). Older references have been retained where they represent foundational or landmark studies. All references are now within the last 10 years except for theoretical foundations (Health Belief Model, Andersen's Behavioural Model).  Revised text: References section (Lines 505–588).  ________________________________________ |
| --- | --- |

|  | Summary of Key Changes in Response to Reviewers  ________________________________________  Additional Revisions Undertaken to Improve Quality  In addition to addressing all reviewer comments, we have made the following improvements:  •COREQ alignment: The manuscript has been reviewed against the COREQ checklist for qualitative studies, and all relevant items have been addressed (e.g., researcher characteristics, relationship with participants, theoretical framework).  •Theory integration: The Health Belief Model and Andersen's Behavioural Model are now more explicitly integrated into the analysis and discussion.  •Language and readability: The manuscript has been carefully proofread for clarity, grammar, and consistency.  •Correction of sampling terminology: All references to "convenience sampling" have been removed and replaced with accurate descriptions of purposive sampling throughout the manuscript and response letter.  •Trustworthiness criteria: Credibility, dependability, confirmability, and transferability are now explicitly addressed in the Methods section with citations (Lincoln & Guba, 1985; Nowell et al., 2017).  ________________________________________  Final Statement  We believe these revisions have substantially improved the manuscript's  methodological rigor, analytical depth, transparency, and alignment with PLOS ONE's qualitative reporting standards. In particular, the collapse of five themes into three analytically coherent themes (Theme 1: Motivation, Theme 2: Barriers, Theme 3: Preferences), the addition of Table 3 (summary of themes and sub-themes), the correction of the sampling description to purposive sampling, and the explicit inclusion of trustworthiness criteria have significantly strengthened the manuscript.  We are deeply grateful for the reviewers' insightful feedback, which has helped us produce a stronger and more impactful manuscript. We have also addressed the data availability concerns by depositing a fully de-identified dataset in a public repository and updating the Data Availability Statement accordingly.  We hope the revised manuscript is now acceptable for publication in PLOS ONE. Thank you for your consideration.  Sincerely,  Noah Nyende (Corresponding Author) on behalf of all co-authors |
| --- | --- |
| Additional Information: |  |
| Question | Response |
| Financial Disclosure  Enter a financial disclosure statement that describes the sources of funding for the work included in this submission. Review the [submission guidelines](http://journals.plos.org/plosone/s/submission-guidelines#loc-financial-disclosure-statement) for detailed requirements. View published research articles from [PLOS ONE](https://journals.plos.org/plosone/) for specific examples.  This statement is required for submission and will appear in the published article if the submission is accepted. Please make sure it is accurate. | The author(s) received no specific funding for this work. |

| Funded studies  Enter a statement with the following details:   - Initials of the authors who received each award - Grant numbers awarded to each author - The full name of each funder - URL of each funder website - Did the sponsors or funders play any role in the study design, data collection and analysis, decision to publish, or preparation of the manuscript? |  |
| --- | --- |
| Did you receive funding for this work? |  |
| Competing Interests  Use the instructions below to enter a competing interest statement for this submission. On behalf of all authors, disclose any [competing interests](http://journals.plos.org/plosone/s/competing-interests) that could be perceived to bias this work—acknowledging all financial support and any other relevant financial or nonfinancial competing interests.  This statement is required for submission and will appear in the published article if the submission is accepted. Please make sure it is accurate and that any funding sources listed in your Funding Information later in the submission form are also declared in your Financial Disclosure statement.  View published research articles from [PLOS ONE](http://journals.plos.org/plosone/) for specific examples. | The authors have declared that no competing interests exist. |

| NO authors have competing interests  Enter: The authors have declared that no competing interests exist.  Authors with competing interests  Enter competing interest details beginning with this statement:  I have read the journal's policy and the authors of this manuscript have the following competing interests: [insert competing interests here] |  |
| --- | --- |
| * typeset |  |
| Data Availability  Authors are required to make all data underlying the findings described fully available, without restriction, and from the time of publication. PLOS allows rare exceptions to address legal and ethical concerns. See the [PLOS Data Policy](http://journals.plos.org/plosone/s/data-availability) and [FAQ](http://journals.plos.org/plosone/s/data-availability#loc-faqs-for-data-policy) for detailed information.  A Data Availability Statement describing where the data can be found is required at submission. Your answers to this question constitute the Data Availability Statement and will be published in the article, if accepted. | No - some restrictions will apply |
| Important: Stating ‘data available on request from the author’ is not sufficient. If your data are only available upon request, select ‘No’ for the first question and explain your exceptional situation in the text box. |  |
| Do the authors confirm that all data underlying the findings described in their manuscript are fully available without restriction? |  |
| Additional data availability information: | Tick here if the URLs/accession numbers/DOIs will be available only after acceptance of the manuscript for publication so that we can ensure their inclusion before publication. |
| Ethics Statement | Ethical approval was obtained from the Mildmay Uganda Research Ethics Committee (MUREC-2024-451) and the Uganda National Council for Science and Technology |

| Enter an ethics statement for this submission. This statement is required if the study involved:   - Human participants - Human specimens or tissue - Vertebrate animals or cephalopods - Vertebrate embryos or tissues - Field research   Write "N/A" if the submission does not require an ethics statement.  General guidance is provided below. Consult the [submission guidelines](http://journals.plos.org/plosone/s/submission-guidelines#loc-guidelines-for-specific-study-types) for detailed instructions. Make sure that all information entered here is included in the Methods section of the manuscript. | (SS3374ES). Written informed consent was obtained from all adult participants and from parents or guardians of minors, with assent from adolescents. Participation was voluntary, and confidentiality and anonymity were maintained throughout the study. |
| --- | --- |

| Format for specific study types  Human Subject Research (involving human participants and/or tissue)   - Give the name of the institutional review board or ethics committee that approved the study - Include the approval number and/or a statement indicating approval of this research - Indicate the form of consent obtained (written/oral) or the reason that consent was not obtained (e.g. the data were analyzed anonymously)   Animal Research (involving vertebrate animals, embryos or tissues)   - Provide the name of the Institutional Animal Care and Use Committee (IACUC) or other relevant ethics board that reviewed the study protocol, and indicate whether they approved this research or granted a formal waiver of ethical approval - Include an approval number if one was obtained - If the study involved non-human primates, add additional details about animal welfare and steps taken to ameliorate suffering - If anesthesia, euthanasia, or any kind of animal sacrifice is part of the study, include briefly which substances and/or methods were applied   Field Research  Include the following details if this study involves the collection of plant, animal, or other materials from a natural setting:   - Field permit number - Name of the institution or relevant body that granted permission |  |
| --- | --- |
|  |  |
| <strong>Data Availability</strong><br> <br>Before publication, Authors are required to make fully available and without restriction all data underlying their findings. Sharing the data underlying your findings increases the visibility, credibility, and impact of your work, while supporting transparency and future scientific discovery. Please see our <a  href="https://journals.plos.org/plosone/s/d ata-availability" target="_blank">PLOS Data Policy page</a> for detailed information on this policy.</p> <br>A <strong>Data Availability | All de-identified focus group discussion (FGD) and key informant interview (KII) transcripts from this study are publicly available in the Zenodo repository at DOI:  10.5281/zenodo.19708023 (https://doi.org/10.5281/zenodo.19708023). Transcripts have been anonymized following BMJ (2010) guidelines, with all direct identifiers removed. |

| Statement</strong>, detailing where the data can be accessed, is required at first submission. Insert your Data Availability  Statement in the box below.</p>  <br>Please see the <a  href="https://journals.plos.org/plosone/s/s ubmission-guidelines#loc-data-reporting" target="_blank">data reporting</a> of our submission guidelines for instructions on what you need to include in your Data Availability Statement. Examples of acceptable Data Availability Statements are included here for reference:</p>  <ol>  <li>The minimal data set [and accompanying code, where generated] is available at [repository name] via [DOI hyperlink]</li>  <li>No data was generated by this study. The following existing data sources were used: [dataset name] from [dataset location] available via [web address or  DOI hyperlink].</li>  <li>For Study Protocols: No datasets were generated or analysed during the current study. All relevant data from this study will be made available upon study completion.</li>  </ol>  <br><i>PLOS allows rare exemptions to address legal and ethical concerns. If you have legal or ethical restrictions, please use the box below to detail these in full sentences for the Journal team to consider.</i></p>  <br>This statement will be typeset if the manuscript is accepted for publication.</p> |  |
| --- | --- |

Manuscript	[Click here to access/download;Manuscript;Manuscript_January](https://www2.cloud.editorialmanager.com/pone/download.aspx?id=44375031&guid=d60205cb-27e0-43f6-b1b9-3e233d4163ce&scheme=1)

[9-2026_Stakeholder Perspectives on ASRH.docx](https://www2.cloud.editorialmanager.com/pone/download.aspx?id=44375031&guid=d60205cb-27e0-43f6-b1b9-3e233d4163ce&scheme=1)

1 **Stakeholder perspectives on adolescent sexual and reproductive**
2 **health service utilisation in Uganda’s Busoga region: A qualitative**

# 3 study

4 Noah Nyende¹* [https://orcid.org/0009-0001-6342-4113,](https://orcid.org/0009-0001-6342-4113) Justine Nanteza², Frank Kiyingi¹, Miph
5 Musoke¹

## 6 Affiliations

7 ¹ Nkumba University, Entebbe, Uganda
8 ² Makerere University School of Public Health, Kampala, Uganda

## 9 Corresponding author

10 Noah Nyende
11 Email: rnnyende2002@yahoo.com

# 12

# Abstract

## 13 Background

14 Adolescents in low- and middle-income countries continue to experience high rates of
15 unintended pregnancy, sexually transmitted infections, and unmet sexual and reproductive health
16 (SRH) needs. In Uganda, these challenges are exacerbated by sociocultural norms,
17 misconceptions, and structural barriers that restrict adolescents’ access to suitable services.

## Objective

This study explored the perspectives of adolescents, community leaders, and health providers on adolescent SRH service utilisation in the Busoga Region of Eastern Uganda, to identify barriers, facilitators, and contextually appropriate strategies for improving adolescent-friendly service delivery.

## Methods

An exploratory qualitative study was conducted using focus group discussions (FGDs) and key informant interviews (KIIs). Thirty-nine adolescents aged 13–19 years participated in four FGDs stratified by sex and residence (urban/rural). Twelve key informants, including health providers, religious leaders, and cultural leaders, were purposively selected. Data were collected between November and December 2024 using semi-structured guides, audio-recorded, transcribed verbatim, and analysed inductively using Braun and Clarke’s thematic analysis framework with support from NVivo 14.

## Results

Five major themes emerged: (1) adolescents’ positive orientation toward SRH prevention amid sexual self-discovery; (2) persistent misconceptions and fears surrounding contraception; (3) access barriers related to distance, privacy, and provider attitudes; (4) strong preferences for free, confidential, peer-informed, and community-based SRH services; and (5) sociocultural and system-level constraints, including early marriage norms, religious prohibitions, poverty, and commodity stock-outs. Adolescents demonstrated motivation to protect their SRH but encountered intersecting barriers that constrained service utilisation.

## Conclusion

Adolescents in the Busoga Region are motivated to access SRH services but face multilayered sociocultural and structural constraints. Strengthening adolescent-friendly service environments, improving provider attitudes, addressing misinformation through community engagement, and expanding school- and community-based outreach may enhance equitable SRH service

utilisation.

# Introduction

Adolescence is a critical developmental stage marked by rapid physical, psychological, and social transitions that have lasting implications for sexual and reproductive health outcomes [1,2]. Globally, adolescents account for a disproportionate burden of unintended pregnancies, unsafe abortions, and sexually transmitted infections, particularly in low- and middle-income countries [2,4]. In sub-Saharan Africa (SSA), structural barriers such as stigma, misconceptions, and limited access contribute to low utilisation of SRH care, and persistently high rates of teenage pregnancy and HIV [3,4]. Kenyan and Ethiopian studies, for example, highlight transport costs, judgmental providers, and restrictive cultural norms as key barriers [4,5].

In Uganda, adolescent SRH indicators remain concerning. Early sexual debut, low contraceptive uptake, and high rates of adolescent pregnancy persist, especially in socioeconomically disadvantaged regions such as Busoga in Eastern Uganda [6,7,8]. Uganda’s adolescent pregnancy rate is 24%, HIV prevalence among 15–19-year-olds is 2.1%, and modern contraceptive use is only 15% [3]. The Busoga Region is particularly affected because of poverty, early marriage norms, and limited services [9]. Despite national adolescent health guidelines [10], utilisation remains low due to stigma, lack of privacy, and misinformation [8, 9]; yet, little is known about how multiple stakeholders, including adolescents, community leaders, and healthcare providers, perceive these barriers and potential solutions. This gap limits the design of locally responsive, adolescent-friendly interventions.

While existing research has generated important insights, most studies focus on adolescents alone or examine health system factors in isolation. There is limited qualitative evidence that triangulates the perspectives of adolescents, community leaders, and health providers within the same sociocultural context [11]. Such triangulation is essential for understanding how community norms, service delivery practices, and adolescent preferences interact to shape SRH service utilisation.

This study aimed to explore adolescents’ attitudes, perceptions, and preferences regarding SRH services in the Busoga Region of Uganda, alongside the perspectives of community leaders and health providers. By examining convergences and divergences across stakeholder groups, the study sought to identify multilevel barriers and facilitators that can inform the design of contextually appropriate, adolescent-friendly SRH interventions. The study was guided by the

Health Belief Model [12] and Andersen’s Behavioural Model [13] to understand how individual perceptions, enabling factors, and sociocultural contexts shape SRH service utilisation.

# Materials and Methods

## Study design

An exploratory qualitative design was employed, guided by a constructivist interpretivist paradigm. This approach was appropriate for examining how adolescents and key community stakeholders construct meaning around SRH service utilisation based on their lived experiences.

## Study setting

The study was conducted in Iganga and Bugweri districts in the Busoga Region of Eastern Uganda. The region is characterised by a mix of rural and urban communities, varying levels of health service availability, and strong cultural and religious influences on adolescent behaviour. Iganga District has relatively better health infrastructure, while Bugweri District is more resource-constrained. Community entry was facilitated through district health officials and local council leaders. The study was conducted between 20^th^ November and 18^th^ December, 2024, at six rural (Namirali, Kiwerere, Bulubandi, Naitandu B, Bunalwenyi C, Makandwa) and six urban (Kasokoso Central II/III, Nakavule Main, Busesa, Butende, Kagamba) sites in Iganga and Bugweri districts. The two districts were selected because they reflect contrasting health system contexts: Bugweri has limited resources and fragile health infrastructure, whereas Iganga is better resourced with stronger systems and staffing, ensuring representation of diverse conditions influencing adolescent SRH service access and utilisation in the region.

## Participants and sampling

Two categories of participants were included. Adolescents aged 13–19 years participated in four FGDs stratified by sex and residence (urban/rural). In total, thirty-nine adolescents were recruited using convenience sampling with support from community mobilisers and youth peer leaders. Twelve key informants were purposively selected based on their roles and knowledge of adolescent SRH, including health providers, religious leaders, and cultural leaders. Sampling followed established qualitative research guidance [14] and proceeded iteratively. Participant recruitment continued until further data no longer added new patterns of meaning, which occurred after 12 key informant interviews and four focus group discussions. Inclusion criteria were adolescents aged 13–19 years residing in the study areas and leaders/providers identified by their communities or workplaces as knowledgeable about adolescent SRH services. (See *Table 1* for participant characteristics.)

Table 1: Characteristics of Study Participants

| **Participant group** | **Number of participants**  **(number of**  **FGDs/KIIs)** | **Age range** | **Gender composition** | **Residence** | **Other characteristics** |
| --- | --- | --- | --- | --- | --- |
| Adolescents  (FGDs) | 39 participants  (4 FGDs) | 13–19 years | Girls: 2 FGDs (13–17 years); Boys:  2 FGDs (17–  19 years) | 2 rural FGDs; 2 urban  FGDs | Education ranged from primary to secondary level; livelihoods included schooling, farming, vocational training, and informal work; predominantly low to moderate |
|  |  |  |  |  | socioeconomic  status |
| Key informants  (KIIs) | 12 (12 KIIs) | Not applicable | Mixed | Rural and urban | 6 health providers (health centres II–IV and district hospital); 6 community leaders  (3 cultural, 3 religious) |

## Data collection

Semi-structured interview guides were developed based on prior literature and national adolescent SRH frameworks. Guides explored attitudes, perceptions, preferences, and barriers related to SRH service utilisation. The tools were prepared in English, translated into Lusoga, and pilot-tested before data collection. FGDs were conducted in neutral community venues to enhance privacy and lasted between 60 and 90 minutes. KIIs were conducted in English or Lusoga, depending on participant preference. All sessions were audio-recorded with consent, and detailed field notes were taken.

## Data analysis

Audio recordings were transcribed verbatim and, where necessary, translated into English. Backtranslation was undertaken for selected transcripts to ensure accuracy and preserve meaning.

Data were analysed inductively using Braun and Clarke’s reflexive thematic analysis approach, following the six analytic phases of familiarisation with the data, coding, generating initial themes, reviewing themes, defining and naming themes, and producing the report [15]. Coding focused on participants’ accounts and meanings rather than pre-defined categories. Two researchers engaged independently with the data during the coding process and subsequently discussed interpretations to refine codes and themes through reflexive dialogue. NVivo version 14 software was used to support data organisation and analytic transparency.

The research team comprised public health and social science researchers with prior experience in adolescent sexual and reproductive health research in Uganda. While the researchers did not have pre-existing relationships with participants, their professional backgrounds and familiarity with SRH programming informed analytic interpretation. Reflexive journaling was used throughout data collection and analysis to document assumptions, positionality, and evolving interpretations, and to minimise undue influence on coding and theme development.

Analysis was guided by the Health Belief Model [12] and Andersen’s Behavioural Model of Health Services Use [13], which were applied analytically to inform data interpretation. Emergent codes and themes were examined in relation to model constructs, including perceived susceptibility, perceived barriers, enabling resources, and sociocultural predisposing factors. This analytic application supported the interpretation of how adolescents’ motivations to protect their sexual and reproductive health interacted with structural and contextual constraints influencing service utilisation.

### Ethical considerations

Ethical approval was obtained from the Mildmay Uganda Research Ethics Committee (MUREC-

2024-451) and the Uganda National Council for Science and Technology (SS3374ES). Written informed consent was obtained from all adult participants and from parents or guardians of minors, with assent from adolescents. Participation was voluntary, and confidentiality and anonymity were maintained throughout the study.

# Results

The results draw on perspectives from adolescent girls aged 13–17 years and boys aged 17–19 years residing in rural and urban communities of Iganga and Bugweri districts in the Busoga Region. Participants included in-school and out-of-school adolescents, as well as married and unmarried adolescents, reflecting diverse social positions and sexual and reproductive health needs. Health providers contributed perspectives based on direct service delivery to adolescents, while cultural and religious leaders provided insight into the broader sociocultural context shaping adolescent behaviour and health-seeking.

The qualitative analysis generated five interrelated themes reflecting adolescents’ lived experiences and the perspectives of community leaders and health providers, presented with selected illustrative quotes:

1. Positive orientation toward SRH prevention amid sexual self-discovery

Adolescents demonstrated a proactive approach to sexual and reproductive health (SRH), driven by clear recognition of the risks of early sexual activity, particularly unintended pregnancy and sexually transmitted infections, including HIV. In focus group discussions, participants emphasised the protective role of SRH services: *“It helps us to prevent sexually transmitted infections and to plan for the future.” (Girl, FGD, Bugweri).*

Health providers corroborated this observation, noting that adolescents actively sought preventive measures due to specific fears: *“Girls fear pregnancies and boys fear illnesses like HIV, so they look for condoms and counselling.” (Health provider, KII, Bugweri).*

This positive orientation toward prevention developed largely independently, in a context where parental guidance on sexual matters was perceived as limited or absent. As one health provider described: *“First of all, what I know is that parents lost interest in their children, so the children are growing themselves by self-discovery to each and everything.” (Health provider, KII, Iganga).*

A religious leader similarly attributed adolescents' engagement with health services to parental negligence: *“It is due to the negligence of the parents, as they do not guide them on when to start sexual issues until they realise their children are seeking services in health facilities.” (Religious leader, KII, Bugweri).*

Thus, amid perceived gaps in parental involvement, adolescents exercised agency through selfdiscovery and help-seeking to safeguard their health and future aspirations.

2. Persistent misconceptions and fears surrounding contraception

Despite the expressed interest in prevention, widespread misconceptions and negative beliefs about modern contraceptive methods persisted, often deterring adolescents from utilising sexual and reproductive health services. Participants frequently cited fears that contraception could cause infertility, cancer, or other long-term health complications: *“Sometimes family planning methods may cause you to become barren.” (Girl, FGD, Iganga).*

*“Use of family planning by a woman who has never had children makes her sterile.” (Boy, FGD, Iganga).*

*“And another thing which we always hear from them is that family planning causes cancer.” (Health provider, KII, Iganga).*

Specific concerns were also raised about condoms: *“We are told they affect women. They can get stuck in women, and that the lubricant in condoms is dangerous.” (Boy, FGD, Iganga).*

Religious perspectives further contributed to these negative views, with some faith leaders expressing disapproval of contraceptive use: *“Some Muslim scholars also permit the use of condoms. About the use of contraceptives, Islam does not recommend.” (Religious leader, KII, Bugweri).*

Health providers and community members noted that such myths were commonly reinforced within families and religious settings, framing contraception as morally inappropriate or risky, which in turn discouraged adolescent uptake of services.

3. Barriers related to distance, privacy, and provider attitudes

Despite adolescents' proactive interest in sexual and reproductive health services, multiple structural and interpersonal barriers hindered access. Distance and transport costs posed significant challenges, especially in rural areas: *“Government health facilities are far, and you can wait many hours before seeing a health worker.” (Boy, FGD, Iganga).*

Privacy concerns were frequently highlighted, with participants noting that small or crowded facilities offered limited confidentiality, increasing the risk of being seen or recognised by community members: *“The place is not enough for the adolescents to acquire knowledge or counselling because an adolescent needs privacy. But according to this health center, as small as it is, we don't have enough privacy for the adolescents.” (Health provider, KII, Iganga).*

Negative attitudes from health workers further deterred service use, with adolescents describing experiences of judgement, harsh treatment, or being made to feel inferior: *“The health workers are tough. Sometimes we fear to open up.” (Girl, FGD, Iganga).*

Community leaders also recognised that social stigma, internalised inferiority, and age-based power imbalances within communities and health facilities fostered shyness and fear of judgement, which prevented young people from seeking care: *“They are despised, which in turn makes them feel inferior. This affects their ability to ask for what they want. This happens a lot at health facilities.” (Cultural leader, KII, Bugweri).*

*“They are shy. They fear going to health facilities because they are young.” (Religious leader, KII, Bugweri).*

These combined geographical, infrastructural, and interpersonal barriers contributed to delayed care-seeking, reduced trust in health facilities, and reluctance to return for subsequent services.

4. Preferences for adolescent-friendly and community-based services

Adolescents consistently expressed a strong preference for sexual and reproductive health services that were free, confidential, accessible, and tailored to their needs, with a particular emphasis on delivery outside traditional health facilities, such as in communities, schools, or through outreach programmes.

Participants highlighted the value of community- and school-based approaches led by peers or adolescent-friendly providers: *“During school holidays, services should come to the community and be led by youth because we believe our peers.” (Girl, FGD, Iganga).*

*“They should be everywhere, community, school and facilities.” (Boy, FGD, Iganga).*

*“They should come up with clubs and specific days of sensitisation.” (Boy, FGD, Bugweri).*

Health providers described existing efforts to reach adolescents through outreaches, school visits, and collaboration with village health teams (VHTs): *“We always provide health education when we go for outreaches, when we go for school health education and also when we talk with the*

*VHTs, tell them toinform them about the importance of our services at the health unit.” (Health provider, KII, Iganga).*

Community and religious leaders also contributed by integrating guidance into existing structures, such as religious gatherings and one-on-one counselling: *“We conduct community meetings. We preach and guide on good conduct during weekly prayers on Fridays.” (Religious leader, KII, Bugweri).*

*“We provide one-on-one guidance for adolescents on what should be done.” (Cultural leader, KII, Bugweri).*

Overall, adolescents and stakeholders favoured decentralised, peer-involved, and community embedded models that reduced barriers related to distance, stigma, and discomfort, thereby increasing the likelihood of service uptake.

5. Sociocultural and system-level constraints

Sociocultural norms and system-level challenges significantly constrained adolescents' access to sexual and reproductive health (SRH) services. Religious and cultural expectations often promoted early marriage or imposed restrictions that limited open engagement with SRH issues:

*“Religion can guide good conduct, but sometimes we marry off adolescents early, and they miss opportunities.” (Religious leader, KII, Bugweri).*

*“Girls should provide for the god of the home before they get married...they should respect their ancestral spirits who are their spiritual husbands.” (Cultural leader, KII, Bugweri).*

Religious practices further complicated access through rigid procedures: “*Rigidity of church procedures, such as testing for HIV first before marriage, is another challenge.” (Religious leader, KII, Iganga).*

At the health system level, frequent shortages of commodities and inadequate staffing undermined service reliability and trust: *“They should stock health facilities with more drugs, and health workers are also few.” (Girl, FGD, Iganga).*

*“Sometimes it is stockouts, especially for family planning commodities. If one comes and does not find the method they want, they don’t come back.” (Health provider, KII, Bugweri).*

These intertwined sociocultural pressures and systemic deficiencies, ranging from normative expectations around marriage and spirituality to persistent resource gaps, collectively restricted adolescents' ability to exercise their agency in seeking and utilising SRH services fully.

# Discussion

This study demonstrates that adolescents in the Busoga Region are motivated to protect their sexual and reproductive health, yet this motivation is frequently constrained by intersecting sociocultural and structural barriers. Analytically applying the Health Belief Model [12] and Andersen’s Behavioural Model 13] illustrates how strong perceived susceptibility to pregnancy and infection coexists with substantial perceived barriers, limited enabling resources, and restrictive community norms, resulting in delayed or foregone service utilisation.

Consistent with previous studies from Uganda and other East African settings, misconceptions about contraception, stigma, confidentiality concerns, and unfriendly provider attitudes emerged as major deterrents to service use [5,6,7,8,9]. Adolescents’ fears regarding infertility associated with contraceptive use mirror findings from sub-Saharan Africa, where myths continue to shape SRH decision-making [4,5,6,9]. The triangulation of perspectives in this study highlights that these barriers are reinforced at multiple levels, including individual, familial, community, and health system, rather than arising solely from adolescents’ knowledge gaps [16].

The findings can be interpreted through the lens of the Health Belief Model (HBM), which explains health-related actions as shaped by factors such as perceived susceptibility, severity, benefits, barriers, cues to action, and self-efficacy [12]. In this study, participants often described strong barriers, especially stigma around the condition and fear of judgment from providers or their community. These concerns seemed to outweigh the perceived benefits of seeking help early, such as better symptom control or improved long-term outcomes. As the HBM predicts, when barriers feel greater than benefits, people tend to delay or avoid care altogether.

Andersen's Behavioural Model of Health Services Use adds a wider view by separating the influences on service use into predisposing, enabling, and need factors [13]. Predisposing elements, like sociocultural norms around gender or community attitudes toward the condition, appeared to discourage help-seeking from the start. At the same time, limited enabling resources (for example, money problems, transport issues, or scarce local services) made it harder to act, even when participants clearly recognised their need for care. Together, the two models show how personal perceptions (from the HBM) combine with structural obstacles (from Andersen's model) to explain the low rates of service use we observed.

This combined perspective highlights the complex reasons behind care-seeking decisions. It also strengthens the case for interventions that address both sides: reducing psychological barriers through anti-stigma efforts and improving practical access through better resources and service availability.

# Implications for policy and practice

The results underscore the need for multi-component interventions that extend beyond facilitybased service provision. Strengthening adolescent-friendly service environments, expanding community- and school-based outreach, engaging parents and faith leaders in addressing misinformation, improving provider communication skills, and ensuring reliable commodity supply are critical steps supported by both participants’ perspectives and existing evidence.

# Strengths and limitations

A key strength of this study is the triangulation of perspectives from adolescents, community leaders, and health providers, which enhances credibility and contextual depth. However, the study was limited to two districts within the Busoga Region, potentially limiting transferability.

Social desirability bias and challenges related to translation may also have influenced findings.

# Conclusion

This qualitative study demonstrates that adolescents in the Busoga Region possess motivation to protect their sexual and reproductive health but face persistent sociocultural, structural, and health system barriers that constrain service utilisation. Addressing misinformation, stigma, confidentiality concerns, and provider attitudes, while expanding adolescent-friendly and community-based service delivery, remains essential for improving adolescent SRH outcomes in similar low-resource settings.

# Data Availability Statement

De-identified qualitative transcripts are available from the corresponding author upon reasonable request and subject to approval by the relevant ethics committees, due to the sensitive nature of the data and the potential risk of participant identification.

# Acknowledgements

I am deeply grateful to the adolescents who participated in this study. I also appreciate the research team for their invaluable contribution. Lastly, to the District Health Offices of Iganga and Bugweri, for their support and facilitation of this research.

# References

1. World Health Organization. Adolescent and young adult health [Internet]. Geneva: World

Health Organization; 2024 [updated 2024 Nov 26; cited 2026 Jan 7]. Available from:

[https://www.who.int/news-room/fact-sheets/detail/adolescents-health-risks-and-solutions.](https://www.who.int/news-room/fact-sheets/detail/adolescents-health-risks-and-solutions)

2. United Nations Population Fund. Adolescent sexual and reproductive health programmes in humanitarian settings: an in-depth look at family planning services [Internet]. New York: UNFPA; 2015 [cited 2026 Jan 7]. Available from: [https://www.unfpa.org/resources/adolescent-sexual-and-reproductive-health-programshumanitarian-settings-0.](https://www.unfpa.org/resources/adolescent-sexual-and-reproductive-health-programs-humanitarian-settings-0)
3. Uganda Bureau of Statistics (UBOS) and ICF. Uganda Demographic and Health Survey

2022 [Internet]. Kampala: UBOS and ICF; 2023 [cited 2026 Jan 7]. Available from: [https://www.ubos.org/wp-content/uploads/publications/UDHS-2022-Report.pdf.](https://www.ubos.org/wp-content/uploads/publications/UDHS-2022-Report.pdf)

4. Sidemo NB, Kerbo AA, Gidebo KD, Wado YD. Socio-ecological analysis of barriers to access and utilization of adolescent sexual and reproductive health services in subSaharan Africa: a qualitative systematic review. Adolesc Health Med Ther. 2023;14:123-

40.

5. Mutea L, Ontiri S, Kadiri F, Michielsen K, Gichangi P. Access to information and use of adolescent sexual reproductive health services: Qualitative exploration of barriers and facilitators in Kisumu and Kakamega, Kenya. PLoS One. 2020;15(11):e0241985.
6. Atuyambe L, Kibira SPS, Bukenya J, Muhumuza C, Apolot RR, Mulenga D. Understanding sexual and reproductive health needs of adolescents: evidence from a formative evaluation in Wakiso district, Uganda. Reprod Health. 2015;12:35.
7. Akatukwasa C, Gumputu B, Amongin D, Atwine D, Mumbere N, Behumbiize P, et al. Integration of HIV-sexual reproductive health services for young people and the barriers at public health facilities in Mbarara Municipality, Southwestern Uganda: a qualitative assessment. Int J Reprod Med. 2019;2019:6725432.
8. Nalwadda G, Mirembe F, Byamugisha J, Faxelid E. Persistent high fertility in Uganda: young people recount obstacles and enabling factors to use of contraceptives. BMC Public Health. 2010;10:530.
9. Nabugoomu JB, Kabonesa C, Kemigisha E, et al. Barriers to adolescent sexual and reproductive health services in Uganda. *Reprod Health*. 2020;17:92. *(Exact DOI available on PubMed)*
10. Ministry of Health Uganda. Adolescent health policy guidelines and service standards [Internet]. Kampala: Ministry of Health Uganda; 2011 [cited 2026 Jan 7]. Available from: [https://library.health.go.ug/sexual-and-reproductive-health/adolescent-health/adolescenthealth-policy-guidelines-and-service.](https://library.health.go.ug/sexual-and-reproductive-health/adolescent-health/adolescent-health-policy-guidelines-and-service)
11. Jonas K, Crutzen R, van den Borne B, Sewpaul R, Reddy P. Healthcare workers' beliefs, motivations and behaviours affecting adequate provision of sexual and reproductive healthcare services to adolescents in Cape Town, South Africa: a qualitative study. BMC

Health Serv Res. 2018;18(1):109.

12. Rosenstock IM. Historical origins of the health belief model. Health Educ Monogr.

1974;2:328–35.

13. Andersen RM. Revisiting the behavioral model and access to medical care: does it matter? J Health Soc Behav. 1995;36:1–10.
14. Guest G, Bunce A, Johnson L. How many interviews are enough? Field Methods.

2006;18:59–82.

15. Braun V, Clarke V. Using thematic analysis in psychology. Qual Res Psychol.

2006;3:77–101.

16. Bronfenbrenner U. The ecology of human development. Cambridge (MA): Harvard

University Press; 1979.

Manuscript	[Click here to access/download;Manuscript;Manuscript.docx](https://www2.cloud.editorialmanager.com/pone/download.aspx?id=44375622&guid=6d7e10b8-ae17-4f8b-9a7a-ccfcde15b3e3&scheme=1)

1 **Stakeholder perspectives on adolescent sexual and reproductive**
2 **health service utilization in Uganda’s Busoga region: A qualitative**

# 3 study

4 Noah Nyende¹* [https://orcid.org/0009-0001-6342-4113,](https://orcid.org/0009-0001-6342-4113) Justine Nanteza², Frank Kiyingi¹, Miph
5 Musoke¹
6 ^1^	Nkumba University School of Sciences, Entebbe, Uganda
7 ^2^	Makerere University School of Public Health, Kampala, Uganda
8* Corresponding author
9 E-mail: rnnyende2002@yahoo.com

# 10 Abstract

## 11 Background

12 Adolescents in low- and middle-income countries face high rates of unintended pregnancy,
13 sexually transmitted infections (STIs), and unmet sexual and reproductive health (SRH) needs. In
14 Uganda, 24% of girls aged 15–19 years have begun childbearing, yet modern contraceptive
15 prevalence among adolescents remains low (15%). These challenges are particularly severe in
16 the Busoga region, where sociocultural norms, poverty, and limited health system capacity
17 hinder access to adolescent-friendly SRH services. Despite national guidelines, utilization
18 remains low. Few studies have triangulated perspectives from adolescents, health providers, and

community/religious leaders. We explored stakeholder views to provide an integrated understanding of factors influencing adolescent SRH service utilization in Busoga, Uganda.

## Methods

An exploratory qualitative study was conducted in Iganga and Bugweri districts. Four focus group discussions with 38 adolescents (13–19 years), stratified by sex, age group, and residence, and 12 key informant interviews with health providers and community leaders were conducted. Adolescents were recruited through peer networks, while key informants were purposively selected. Data were collected (November–December 2024), transcribed verbatim, and analyzed inductively using thematic analysis with NVivo 14. Ethical approval and informed consent/assent were obtained.

## Results

Adolescents, health providers, and community stakeholders described SRH service utilization as shaped by adolescent motivation alongside persistent barriers. Adolescents showed strong preventive intent, but uptake was limited by misconceptions about contraception, fears of infertility, long distances, lack of privacy, negative provider attitudes, and commodity stock-outs. Providers and community leaders echoed concerns around misinformation, limited parental guidance, moral norms, and structural constraints. All stakeholders converged on the need for confidential, accessible, and peer- and community-based adolescent SRH services.

## Conclusion

Adolescents in Busoga are motivated to use SRH services, but intersecting socio-structural and system barriers limit utilization. Strengthening adolescent-friendly services, improving provider responsiveness, addressing misinformation, and expanding community-based delivery are essential to enhance equitable access.

### Introduction

Adolescence is a critical developmental stage marked by rapid physical, psychological, and social transitions that have lasting implications for sexual and reproductive health (SRH) outcomes [1,2]. Globally, adolescents account for a disproportionate burden of unintended pregnancies, unsafe abortions, and sexually transmitted infections (STIs), particularly in low- and middle-income countries [2]. In sub-Saharan Africa (SSA), recent evidence continues to highlight persistent structural, sociocultural, and health system barriers that drive low utilization of SRH services and sustain high rates of teenage pregnancy and HIV [3,4]. Kenyan and Ethiopian studies, for example, identify transport costs, judgmental provider attitudes, restrictive cultural and gender norms, fear of stigma, and lack of privacy as recurring obstacles [5,6].

In Uganda, adolescent SRH indicators remain concerning. Early sexual debut, low contraceptive uptake, and high rates of adolescent pregnancy persist [7–9]. Uganda’s adolescent pregnancy rate (teenage childbearing) is approximately 24% among girls aged 15–19 years, HIV prevalence among 15–19-year-olds is 2.1%, and modern contraceptive use among 15–19-year-olds is only 15 [10]. The Busoga Region is particularly affected because of poverty, early marriage norms, and limited services [11]. Recent studies in the region confirm high non-utilization of modern contraceptives and SRH services, influenced by lack of confidence in discussing contraception, perceived inaccessibility of services, and limited interaction with community health workers [12].

Despite national adolescent health guidelines [13], utilization remains low due to stigma, lack of privacy, and misinformation [8,9]. Yet, little is known about how multiple stakeholders, including adolescents themselves, community and religious leaders, and healthcare providers, perceive these barriers and potential solutions within the same sociocultural context. Most existing research has focused on adolescents alone or examined isolated health system factors [4,14]. This study addresses that gap by qualitatively triangulating perspectives from adolescents, community and religious leaders, and health providers in the Busoga Region. This multistakeholder approach provides an integrated understanding of how community norms, service delivery practices, and adolescent preferences interact to shape SRH service utilization, thereby offering more nuanced insights for designing locally responsive, adolescent-friendly interventions. This integrated lens complements single-perspective studies and responds to calls for multi-level approaches to adolescent SRH in SSA [4,15].

To address this gap**,** the present study explored adolescents’ attitudes, perceptions, and preferences regarding SRH services in the Busoga Region of eastern Uganda, alongside the perspectives of community leaders and health providers. By examining convergences and divergences across stakeholder groups, the study sought to identify multilevel barriers and facilitators that can inform the design of contextually appropriate, adolescent-friendly SRH interventions. The study was guided by the Health Belief Model [16] and Andersen’s Behavioural Model [17] to understand how individual perceptions, enabling factors, and sociocultural contexts shape SRH service utilization.

# Materials and Methods

## Study design

An exploratory qualitative design was employed, guided by a constructivist-interpretivist paradigm. This approach was appropriate for examining how adolescents and key community stakeholders construct meaning about SRH service utilization from their lived experiences.

## Study setting

The study was conducted in Iganga and Bugweri districts in the Busoga Region of Eastern Uganda. The Busoga Region is characterised by a mix of rural and urban communities, varying levels of health service availability, and strong cultural and religious influences on adolescent behaviour. Community entry was facilitated through district health officials and local council leaders. The study was conducted between 20 November and 18 December 2024 at six rural (Namirali, Kiwerere, Bulubandi, Naitandu B, Bunalwenyi C, Makandwa) and six urban

(Kasokoso Central II/III, Nakavule Main, Busesa, Butende, Kagamba) sites in Iganga and Bugweri districts. The two districts were selected because they reflect contrasting health system contexts: Bugweri has limited resources and fragile health infrastructure, whereas Iganga is better resourced with stronger systems and staffing, ensuring representation of diverse conditions influencing adolescent SRH service access and utilization in the region.

## Participants and sampling

Two categories of participants were included in this study: adolescents and key informants. Adolescents were purposively sampled to capture diverse perspectives on SRH service utilization across gender, age, and residential contexts. Specifically, younger female adolescents (13–17 years) and older male adolescents (17–19 years) were selected to reflect gendered patterns in SRH vulnerability and engagement. Younger females are often at earlier stages of sexual debut and face heightened risks related to early pregnancy and barriers to accessing services. At the same time, older males are more likely to be sexually active and engaged in SRH-related decision-making. The overlap at age 17 was intentional to capture transitional developmental experiences between mid- and late adolescence.

Adolescents were further stratified by residence (rural/urban) to account for contextual differences in access to SRH information and services. In total, 38 adolescents participated in four focus group discussions (FGDs), with recruitment facilitated through community mobilisers and youth peer leaders. Inclusion criteria for adolescents were: aged 13–19 years, residence in the study area for at least six months, and willingness to participate.

Key informants were selected using purposive sampling based on their roles, experience, and knowledge of adolescent SRH. These included health providers (such as nurses and clinical officers delivering SRH services) and community leaders, including religious and cultural leaders. Religious leaders were drawn from established faith-based institutions (churches and mosques) and held formal roles (clergy, pastors, or imams). In contrast, cultural leaders were recognized traditional authorities (clan leaders and elders). Health providers contributed perspectives based on their direct experience delivering services to adolescents, while cultural and religious leaders offered insight into the broader sociocultural context shaping adolescent health-seeking behaviour. A total of twelve key informant interviews (KIIs) were conducted.

Sampling proceeded iteratively, guided by principles of qualitative inquiry [18], with participant recruitment continuing until no new patterns of meaning emerged from the data. Data saturation was reached after four FGDs and twelve KIIs.

## Data collection

Semi-structured interview guides were developed based on prior literature and national adolescent SRH frameworks [13]. The guides explored adolescents’ attitudes, perceptions, preferences, and barriers to SRH service utilization. Tools were prepared in English, translated into Lusoga, and pilot-tested before data collection. Pilot testing supported the credibility of the tools by ensuring the clarity, cultural appropriateness, and relevance of the questions to the study context [19].

Four FGDs, each comprising 9–10 participants, were conducted with adolescents. Discussions were held in Lusoga, in neutral community venues and lasted 60–90 minutes. Each session was facilitated by a trained moderator of the same sex as participants, where possible, and supported by a note-taker. No external observers were present. Moderation followed a semi-structured guide, with probing and neutral phrasing used to manage sensitive topics and encourage participation. Prolonged engagement during discussions and the use of probing techniques enhanced credibility by allowing participants to express detailed and in-depth perspectives.

A total of twelve KIIs were conducted with health providers and community leaders. Interviews were conducted in either English or Lusoga, depending on participant preference. The inclusion of multiple participant categories enabled triangulation of perspectives, further strengthening the credibility of the findings [19].

All participants were approached through peer mobilization or community intermediaries, provided with study information, and invited to participate voluntarily. A small number of adolescents declined participation due to time constraints or discomfort discussing SRH in group settings, while no refusals were recorded among key informants. All FGDs and KIIs were audiorecorded with consent, and detailed field notes were taken to complement the recordings. The combination of audio recordings and field notes supported confirmability by ensuring that findings were grounded in participants’ accounts.

## Data analysis

Data collection and analysis proceeded iteratively, with emerging insights informing subsequent interviews. Saturation was assessed through regular team debriefs and concurrent review of transcripts, and data collection ceased at four FGDs and twelve KIIs when no new themes emerged, categories were well developed, and additional interviews were unlikely to yield meaningful insights. Regular debriefings also contributed to dependability by providing opportunities to review and refine emerging interpretations systematically [20].

Audio recordings were transcribed verbatim and, where necessary, translated into English. Backtranslation was undertaken for selected transcripts to ensure accuracy and preserve meaning. All data were anonymized and stored on password-protected devices accessible only to the research team, in line with ethical requirements. Back-translation and careful data management procedures enhanced credibility and confirmability by preserving the authenticity and integrity of the data.

Data were analyzed inductively using Braun and Clarke’s reflexive thematic analysis, following six phases: familiarisation with the data, coding, generating initial themes, reviewing themes, defining and naming themes, and producing the report [21]. Coding focused on participants’ accounts and meanings rather than pre-defined categories. Two researchers engaged independently with the data during the coding process and subsequently discussed interpretations to refine codes and themes through reflexive dialogue. NVivo version 14 software was used to support data organisation and analytic transparency. Independent coding and collaborative theme refinement strengthened dependability by enhancing consistency in the analytic process, while the use of NVivo supported transparency and confirmability of the findings [21].

The research team comprised public health and social science researchers with prior experience in adolescent SRH research in Uganda. While the researchers did not have pre-existing relationships with participants, their professional backgrounds and familiarity with SRH programming informed analytic interpretation. Reflexive journaling was used throughout data collection and analysis to document assumptions, positionality, and evolving interpretations, and to minimise undue influence on coding and theme development. Reflexive practices further supported confirmability by ensuring that interpretations remained grounded in the data rather than researcher predispositions [19]. In addition, a comprehensive audit trail was maintained, including transcripts, field notes, coding frameworks, and analytic decisions, to enhance transparency and dependability of the research process.

Analysis was guided by the Health Belief Model [16] and Andersen’s Behavioural Model of Health Services Use [17], which were applied analytically to inform data interpretation. Emerging codes and themes were examined in relation to model constructs, including perceived susceptibility, perceived barriers, enabling resources, and sociocultural predisposing factors. This analytic application supported the interpretation of how adolescents’ motivations to protect their SRH interacted with structural and contextual constraints influencing service utilization.

### Ethical considerations

The study was approved by the Mildmay Uganda Research Ethics Committee (MUREC-2024451) and registered by the Uganda National Council for Science and Technology (SS3374ES). Written informed consent was secured from all adult participants and from parents/guardians of minors, with adolescents' assent. Participation was voluntary, and confidentiality and anonymity were maintained. Adolescents in FGDs received modest reimbursement for transport and time. Key informants received no compensation, as interviews were conducted at their workplaces or other convenient locations at no cost. We followed the consolidated criteria for reporting qualitative studies (COREQ) in reporting the findings [22]

**Results**

## Characteristics of the study participants

A total of four FGDs were conducted across Bugweri and Iganga districts, stratified by sex and residence. The composition of the focus group discussions is shown in Table 1.

## Table 1. Composition of Focus Group Discussions (FGDs) by Site

| **FGD No. Gender Residence/Site Mean Age Sc** 1 Male Bugweri Rural 17.9 | | **hooling Status No. of P** Mostly out | **articipants** 10 |
| --- | --- | --- | --- |
| 2 Female Bugweri Urban | 16.0 | Mostly in | 9 |
| 3 Male Iganga Urban | 18.0 | Mostly in | 10 |
| 4 Female Iganga Rural | 15.2 | Mostly in | 9 |

Male participants were drawn from rural Bugweri and urban Iganga, while female participants were recruited from urban Bugweri and rural Iganga. The mean age ranged from 15.2 to 18.0 years. Participants in rural Bugweri were predominantly out of school, whereas those in other sites were mostly in school. Each FGD comprised 9–10 participants.

A total of 12 KIIs were conducted with service providers, religious leaders, and cultural leaders across the two districts.

Table 2 shows the composition of the 12 KIIs conducted with service providers, religious leaders, and cultural leaders.

## Table 2. Composition of Key Informant Interviews (KIIs)

| **Participant Group** | **Gender** | **District** | **Rural**  **(n)** | **Urban**  **(n)** | **Participants per KII** | **Total Participants** |
| --- | --- | --- | --- | --- | --- | --- |
| Service providers | Female | Iganga | 1 | 1 | 1 | 2 |
|  |  | Bugweri | 1 | 1 | 1 | 2 |
| Religious leaders | Male | Iganga | 1 | 1 | 1 | 2 |
|  |  | Bugweri | 1 | 1 | 1 | 2 |
| Cultural leaders | Male | Iganga | 1 | 1 | 1 | 2 |
|  |  | Bugweri | 1 | 1 | 1 | 2 |
| **Total** | — | — | **6** | **6** | **1** | **12** |

Service providers were all female, while religious and cultural leaders were male. Each stakeholder group was equally represented across rural and urban settings (n=6 each).

Table 3 summarises the three main themes and sub-themes that emerged from the qualitative analysis.

## Table 3. Overview of main themes and sub-themes on adolescent SRH service utilization

| **Theme** | **Description** | **Key sub-themes** |
| --- | --- | --- |
| 1. Positive orientation toward SRH prevention amid sexual selfdiscovery | Adolescents demonstrated proactive health-seeking behaviour driven by perceived risks of pregnancy and STIs, often navigating SRH decisions independently due to limited parental guidance. | Perceived susceptibility to pregnancy and STIs; Gendered motivations for prevention; Selfdirected learning and limited parental communication |
| 2. Barriers to SRH service utilization | Multiple interrelated barriers at cognitive, structural, interpersonal, and sociocultural levels limited | Misconceptions and fears surrounding contraception; Structural, interpersonal, and sociocultural barriers (distance, |
|  | adolescents’ access to and utilization of SRH services. | privacy, provider attitudes, stigma); Sociocultural and system-level constraints (norms, early marriage, stockouts) |
| 3. Preferences for adolescentfriendly and community-based  services | Adolescents preferred decentralised, confidential, and youth-friendly service models delivered through community, school, and peer-led approaches to improve accessibility and trust. | Community- and school-based service delivery; Peer-led approaches and trust in information from fellow youth; Outreach and integration with existing community structures |

The qualitative analysis generated three main themes reflecting adolescents’ lived experiences with SRH services, together with perspectives from health providers, religious, and cultural leaders (Table 3). These themes are analytically interconnected and demonstrate a clear progression from individual-level motivation to broader contextual constraints. Adolescents showed a proactive orientation and agency in seeking SRH services to protect themselves from unintended pregnancy and STIs (Theme 1). However, their efforts were significantly limited by multiple barriers operating at cognitive, structural, interpersonal, sociocultural, and system levels (Theme 2). In response, adolescents expressed clear preferences for decentralised, confidential, and peer-supported adolescent-friendly services (Theme 3).

## Detailed thematic findings

### 1. Positive orientation toward SRH prevention amid sexual self-discovery

Adolescents demonstrated a proactive orientation toward seeking SRH services, driven by a clear awareness of the risks associated with early sexual activity, including unintended pregnancies and STIs such as HIV. Many participants linked SRH services to protecting their future aspirations, particularly continuing education and avoiding early marriage.

Female adolescents frequently emphasised the protective role of SRH services. One participant explained:

*“It can help prevent early marriages. Some girls get pregnant, and then the family forces them to marry the boy. But if you go for SRH services, you get advice early, and you can avoid that kind of situation before it even starts.” (Female adolescent, FGD 2, rural*

*Iganga)*

Another girl highlighted the importance of knowledge and personal agency:

*“When we go to health workers, they educate us that if you don’t have money, you can ask your parents. If they don’t have it, you should be patient because not everything comes immediately. Adolescents need to be sharp. Even if a friend influences you, you don’t just follow. A knowledgeable girl can then positively influence others.” (Female adolescent, FGD 1, urban Bugweri)*

Male adolescents similarly recognised these benefits, often stressing personal responsibility and focus on school. One boy stated:

*“To prevent unintended pregnancies and infections like HIV. If you start having sex without knowing what you’re doing, you can easily get a girl pregnant or catch diseases. Seeking these services teaches you how to protect yourself and stay safe so you don’t carry heavy burdens while still young.” (Male adolescent, FGD 3, urban Iganga)*

Another male participant highlighted the practical value of services available locally:

*“It helps you prevent contracting STIs like HIV, syphilis, and others. You get health education at places such as the health centre, and they also teach you about using condoms properly. That way, you protect yourself instead of just risking everything.”*

*(Male adolescent, FGD 4, rural Bugweri)*

This positive orientation was often connected to broader life goals, with participants describing how access to SRH services reduced worries about pregnancy or infection and supported better school performance. However, this sense of agency developed amid limited parental guidance. Both health providers and religious leaders highlighted significant gaps in parent–child communication regarding sexual health.

One health provider explained the situation clearly:

*“First of all, what I know is that parents lost interest in their children, so the children are growing themselves by self-discovery to everything.” (Health provider, KII, rural*

*Iganga)*

A religious leader echoed this concern about parental negligence:

*“It is due to the negligence of the parents, as they do not guide them on when to start sexual issues until they realise their children are seeking services in health facilities.” (Religious leader, KII, urban Buugweri)*

A cultural leader similarly noted that many parents have stopped advising their children: *“Parents have stopped advising their children about bad conduct. We leaders must remind them. Health workers should invite us to community meetings so we can share our cultural values with the people.” (Cultural leader, KII, rural Iganga)*

In response, adolescents engaged in self-directed learning and independent health-seeking behaviours. This reflects a process of sexual self-discovery shaped by curiosity, peer influence, and perceived risk. However, this emerging agency was often constrained by incomplete or inaccurate information, particularly regarding contraception, which shaped their subsequent attitudes and decision-making.

Adolescents, therefore, turned to health services as an important source of information and support to help them navigate their developing sexuality and prevent negative outcomes.

### 2. Barriers to SRH service utilization

Adolescents faced multiple, interrelated barriers that limited their access to and utilization of SRH services. These barriers operated at cognitive, structural, interpersonal, and sociocultural levels.

### *Misconceptions and fears surrounding contraception*

Despite strong motivation to prevent unintended pregnancy, many adolescents held widespread misconceptions about modern contraceptive methods. Both female and male participants expressed deep fears that contraceptive use could cause long-term reproductive harm, particularly infertility:

*“Sometimes, I hear that family planning methods may cause you to become barren, and that scares me.” (Female adolescent, FGD 2, rural Iganga)*

A male participant echoed this concern:

*“Use of family planning by a woman who has never had children makes her sterile.”*

*(Male adolescent, FGD 3, urban Iganga)*

Adolescents also shared additional misinformation about method safety and use:

*“We are told they affect women. They can get stuck in women, and that the lubricant in condoms is dangerous.” (Male adolescent, FGD 3, urban Iganga)*

Health providers confirmed the persistence of such misconceptions, noting that adolescents frequently worried about serious health consequences:

*“Some fear that if they use family planning methods, they will never get pregnant when they grow up. Others believe family planning methods cause cancer, that they can lose the uterus, or get fibroids. Because of these beliefs, some refuse to use family planning.”*

*(Health provider, KII, urban Bugweri)*

*“And another thing which we always hear from them is that family planning causes cancer.” (Health provider, KII, urban Iganga)*

Religious perspectives further shaped normative attitudes toward contraception. A religious leader explained:

*“Some Muslim scholars also permit the use of condoms, but about the use of contraceptives, Islam does not recommend.” (Religious leader, KII, Bugweri)*

These fears of infertility, misinformation about health risks, and religiously influenced norms often outweighed the perceived benefits of contraception and discouraged uptake.

### *Structural, interpersonal, and sociocultural barriers*

Adolescents also encountered practical and relational obstacles when seeking services. Structural challenges, particularly long distances to facilities, high costs, and long waiting times, were frequently mentioned:

*“Government health facilities are far, and you can wait many hours before seeing a health worker.” (Male adolescent, FGD 3, urban Iganga)*

A female adolescent added:

*“For example, here, if you go at noon, you might not come back until 4 or 5 pm. So, I end up thinking, why don’t I just stay at home or go somewhere else… instead of wasting time at the health facility.” (Female adolescent, FGD 1, urban Bugweri)*

Privacy and confidentiality were major concerns. Participants noted that small or overcrowded health centres made confidential counselling difficult. A health provider confirmed this challenge:

*“The place is not enough for the adolescents to acquire knowledge or counselling because an adolescent needs privacy. But according to this health center, as small as it is, we don't have enough privacy for the adolescents.” (Health provider, KII, rural*

*Iganga)*

Negative provider attitudes further discouraged service use. Adolescents described experiences of being judged, spoken to harshly, or belittled by health workers. One girl recounted:

*“… the health workers come late, and they are arrogant and proud… you can even go back home without being attended to.” (Female adolescent, FGD 2, rural Iganga)*

Community leaders placed these experiences within broader social contexts of stigma and power imbalances. A cultural leader observed:

*“They are despised, which in turn makes them feel inferior. This affects their ability to ask for what they want. This happens a lot at health facilities.” (Cultural leader, KII,*

*Bugweri)*

A religious leader added that shyness and fear related to their young age also prevented many adolescents from seeking care:

*“They are shy. They fear going to health facilities because they are young.” (Religious leader, KII, Bugweri)*

### *Sociocultural and system-level constraints*

Sociocultural norms and systemic challenges further shaped adolescents’ access to SRH services. Religious and cultural leaders emphasised norms around morality, sexuality, and marriage that often influenced when and how adolescents could engage with services:

*“Religion can guide good conduct, but sometimes we marry off adolescents early, and they miss opportunities.” (Religious leader, KII, Bugweri)*

*“Girls should provide for the god of the home before they get married...they should respect their ancestral spirits who are their spiritual husbands.” (Cultural leader, KII,*

*Bugweri)*

At the same time, health system constraints restricted access to services. Providers highlighted persistent challenges related to the availability of commodities:

*“Sometimes it is stockouts, especially for family planning commodities. If one comes and does not find the method they want, they don’t come back.” (Health provider, KII,*

*Bugweri)*

Adolescents similarly recognised these limitations and expressed the need for improved service readiness:

*“They should stock health facilities with more drugs…” (Female adolescent, FGD 2,*

*Iganga)*

Institutional religious practices were also identified as structural barriers. One religious leader noted:

*“Rigidity of church procedures, such as testing for HIV first before marriage, is another challenge.” (Religious leader, KII, Iganga)*

Overall, cognitive, structural, interpersonal, sociocultural, and system-level barriers interacted to constrain adolescents’ utilisation of SRH services.

#### 3. Preferences for adolescent-friendly and community-based services

In response to the multiple barriers described above, adolescents expressed strong preferences for SRH services that were accessible, confidential, and tailored to their specific needs. Many emphasised the importance of decentralised delivery through community- and school-based platforms:

*“They should be everywhere—community, school and facilities.” (Male adolescent, FGD*

*3, urban Iganga)*

*“They should come up with clubs and specific days of sensitisation.” (Male adolescent,*

*FGD 4, rural Bugweri)*

Peer-led approaches were particularly valued. A female adolescent stated:

*“During school holidays, services should come to the community and be led by youth because we believe our peers.” (Female adolescent, FGD 2, rural Iganga)*

Health providers described ongoing efforts to reach adolescents through outreach activities, school visits, and collaboration with village health teams (VHTs):

*“We always provide health education when we go for outreaches, when we go for school health education, and also when we talk with the VHTs, tell them to inform them about the importance of our services at the health unit.” (Health provider, KII, Iganga)* Community and religious leaders reported integrating adolescent guidance into existing social and institutional structures, including religious gatherings and personalised counselling:

*“We conduct community meetings. We preach and guide on good conduct during weekly prayers on Fridays.” (Religious leader, KII, Bugweri)*

*“We provide one-on-one guidance for adolescents on what should be done.” (Cultural leader, KII, Bugweri)*

These findings show both convergence and variation in how adolescent-friendly SRH services are conceptualised across stakeholders. Adolescents emphasised accessibility, confidentiality, and peer-based engagement, while providers focused on outreach-oriented service delivery, and community and religious leaders underscored moral guidance and behavioural regulation. Across these perspectives, common barriers, including contraceptive misinformation, limited privacy, long distances to facilities, negative provider attitudes, and sociocultural expectations, were consistently reported as shaping adolescents’ experiences with SRH services.

# Discussion

This study demonstrates that adolescents in the Busoga Region are motivated to protect their sexual and reproductive health, yet this motivation is frequently constrained by intersecting sociocultural and structural barriers. Analytically applying the Health Belief Model [16] and Andersen’s Behavioural Model [17] illustrates how strong perceived susceptibility to pregnancy and infection coexists with substantial perceived barriers, limited enabling resources, and restrictive community norms, resulting in delayed or foregone service utilization.

Consistent with previous studies from Uganda and other East African settings, misconceptions about contraception, stigma, confidentiality concerns, and unfriendly provider attitudes emerged as major deterrents to service use [3–9,14,15]. These findings resonate strongly with broader evidence across SSA. A qualitative systematic review synthesizing studies from multiple SSA countries identified similar multi-level barriers, including misconceptions about SRH services, fear of stigma from family and community, low self-esteem, financial constraints, unsupportive family communication, judgmental provider attitudes, lack of privacy, and commodity shortages [4]. In Ethiopia, adolescents similarly reported fear of being seen by family or community members, restrictive social and gender norms, and inadequate integration of SRH information in health facilities as key obstacles [6]. Studies in Nigeria and Ghana have highlighted individuallevel barriers such as inadequate knowledge and poor attitudes toward services, compounded by strong social and religious norms that constrain open engagement with SRH issues [23,24].

Adolescents’ fears regarding infertility associated with contraceptive use mirror findings from across SSA and other low- and middle-income settings, where myths about long-term health complications (including infertility, cancer, or sterility) continue to shape SRH decision-making and deter uptake, particularly among nulliparous young people [4–6,9]. Such concerns are often reinforced by community, religious, and peer networks.

The triangulation of perspectives in this study (from adolescents, community/religious leaders, and health providers) highlights that these barriers are reinforced at multiple levels (individual, familial, community, and health system) rather than arising solely from adolescents’ knowledge gaps [4,25]. This multi-stakeholder lens complements earlier single-perspective or facility-focused studies and aligns with socio-ecological approaches used in other SSA contexts [4,15].

The findings can be interpreted through the lens of the HBM, which posits that health-related actions are shaped by perceived susceptibility, severity, benefits, barriers, cues to action, and selfefficacy [16]. In this study, strong barriers (particularly stigma and fear of judgment from providers or the community) often outweighed perceived benefits of early service use, such as better symptom control or improved long-term outcomes. As the HBM predicts, when barriers exceed benefits, individuals tend to delay or avoid care. Comparable applications of the HBM in adolescent SRH research in Ethiopia and Ghana have shown that perceived social risks, including shame and fear of disclosure, frequently outweigh health benefits, leading to delayed utilization [6].

Andersen’s Behavioural Model complements this by distinguishing predisposing, enabling, and need factors [17]. Predisposing elements, such as sociocultural norms around gender or community attitudes toward the condition, appeared to discourage help-seeking from the outset. Concurrently, limited enabling resources (for example, money problems, transport issues, or scarce local services) hindered action even when the need was recognized. This combined perspective reveals how individual perceptions (HBM) interact with structural obstacles (Andersen’s model) to explain the low service utilization observed, consistent with patterns in Tanzania and other low and middle-income countries (LMICs) where distance, cost, and service availability interact with sociocultural norms [26].

Importantly, evidence from SSA indicates that integrated, multi-component interventions (combining youth-friendly service provision, community engagement, and school-based programs) are more effective than single-component strategies for improving adolescent SRH outcomes [27,28]. Such approaches address both psychological barriers through anti-stigma efforts and improved communication, and practical access issues through enhanced resources, privacy, and service availability.

Overall, the present findings underscore shared structural and sociocultural challenges to adolescent SRH service utilization across diverse settings while highlighting the value of contextspecific, theory-informed interventions in the Busoga Region and similar East African contexts.

# Implications for policy and practice

The results underscore the need for multi-component interventions that extend beyond facilitybased service provision. At the health system level, there is a need to strengthen adolescentfriendly service delivery through targeted training of health providers in non-judgmental communication, confidentiality, and youth-responsive care, alongside the integration of adolescent SRH competencies into routine in-service training and supervision frameworks. Health facilities should also be supported to establish dedicated adolescent service corners, flexible service hours, and privacy-enhancing infrastructure to improve acceptability and utilization.

Expanding community- and school-based outreach programmes, including the use of trained peer educators and collaboration with VHTs, can improve accessibility and trust in SRH services. In addition, structured community engagement strategies are needed to involve parents, religious leaders, and cultural gatekeepers in addressing misconceptions about contraception and promoting supportive norms around adolescent SRH. This may include dialogue forums, faith-based sensitisation initiatives, and parent–adolescent communication programmes.

To address persistent system barriers, policymakers should prioritise strengthening supply chain management systems to ensure consistent availability of contraceptives and essential SRH commodities, particularly in rural facilities. Finally, integrating adolescent SRH indicators into routine monitoring systems and strengthening accountability mechanisms can support the sustained delivery of responsive and equitable services.

# Strengths and limitations

A key strength of this study is the triangulation of perspectives from adolescents, community leaders, and health providers, which enhances credibility and contextual depth. However, the study was limited to two districts within the Busoga Region, potentially limiting transferability. While the findings offer insights that may be relevant to other similar low-resource settings with comparable sociocultural norms, economic challenges, and health system constraints, they are primarily grounded in the specific context of the Busoga region. Social desirability bias and challenges related to translation may also have influenced findings.

# Conclusion

This qualitative study demonstrates that adolescents in the Busoga Region of eastern Uganda have the motivation to protect their SRH, but face persistent sociocultural, structural, and health system barriers that constrain service utilization. Addressing misinformation, stigma, confidentiality concerns, and negative provider attitudes, alongside expanding adolescentfriendly and community-based service delivery, remains essential for improving adolescent SRH outcomes. In particular, decentralisation of SRH services through community- and school-based platforms, outreach programmes, and peer-led approaches represents a critical strategy for overcoming the distance, access, and confidentiality barriers identified in this study. Such approaches, however, warrant careful adaptation to local contexts rather than direct replication.

# Data availability statement

All de-identified focus group discussion (FGD) and key informant interview (KII) transcripts from this study are publicly available in the Zenodo repository at DOI:

<https://doi.org/10.5281/zenodo.19708023>

10.5281/zenodo.19708023 ([)](https://doi.org/10.5281/zenodo.19708023). Transcripts have been

anonymized following BMJ (2010) guidelines, with all direct identifiers removed.

# Acknowledgements

We are deeply grateful to the adolescents who participated in this study. We also appreciate the research team for their invaluable contribution. Lastly, to the District Health Offices of Iganga and Bugweri, for their support and facilitation of this research.

# Author contributions

**Conceptualization**: Noah Nyende, Justine Nanteza, Frank Kiyingi, Miph Musoke.

**Data curation:** Noah Nyende, Justine Nanteza.

**Formal analysis:** Noah Nyende, Justine Nanteza.

**Investigation:** Noah Nyende.

**Methodology:** Noah Nyende, Justine Nanteza.

**Supervision:** Frank Kiyingi, Miph Musoke.

**Validation:** Frank Kiyingi, Miph Musoke.

**Writing – original draft:** Noah Nyende.

**Writing – review & editing:** Justine Nanteza, Frank Kiyingi, Miph Musoke.

# References

1. World Health Organization. Adolescent and young adult health. In: World Health Organization [Internet]. 26 Nov 2024. Available: https://www.who.int/news-room/factsheets/detail/adolescents-health-risks-and-solutions
2. United Nations Population Fund. Adolescent sexual and reproductive health programs in humanitarian settings: an in-depth look at family planning services. New York: United Nations Population Fund; 2012. Available: https://www.unfpa.org/resources/adolescent-sexual-andreproductive-health-programs-humanitarian-settings-0
3. Melesse DY, Mutua MK, Choudhury A, Wado YD, Faye CM, Neal S, et al. Adolescent sexual and reproductive health in sub-Saharan Africa: who is left behind? BMJ Glob Health. 2020;5: e002231. doi:10.1136/bmjgh-2019-002231
4. Sidamo NB, Kerbo AA, Gidebo KD, Wado YD. Socio-Ecological Analysis of Barriers to Access and Utilization of Adolescent Sexual and Reproductive Health Services in Sub-Saharan Africa: A

Qualitative Systematic Review. Open Access J Contracept. 2023;Volume 14: 103–118.

doi:10.2147/OAJC.S411924

5. Mutea L, Ontiri S, Kadiri F, Michielesen K, Gichangi P. Access to information and use of adolescent sexual reproductive health services: Qualitative exploration of barriers and facilitators in Kisumu and Kakamega, Kenya. Baysari MT, editor. PLOS ONE. 2020;15: e0241985. doi:10.1371/journal.pone.0241985
6. Sidamo N, Kerbo A, Gidebo K, Wado YD. Exploring Barriers to Accessing Adolescents Sexual and Reproductive Health Services in South Ethiopia Regional State: A Phenomenological Study Using Levesque’s Framework. Adolesc Health Med Ther. 2024;Volume 15: 45–61.

doi:10.2147/AHMT.S455517

7. Atuyambe LM, Kibira SPS, Bukenya J, Muhumuza C, Apolot RR, Mulogo E. Understanding sexual and reproductive health needs of adolescents: evidence from a formative evaluation in Wakiso district,

Uganda. Reprod Health. 2015;12: 35. doi:10.1186/s12978-015-0026-7

8. Akatukwasa C, Bajunirwe F, Nuwamanya S, Kansime N, Aheebwe E, Tamwesigire IK. Integration of HIV-Sexual Reproductive Health Services for Young People and the Barriers at Public Health

Facilities in Mbarara Municipality, Southwestern Uganda: A Qualitative Assessment. Int J Reprod

Med. 2019;2019: 1–11. doi:10.1155/2019/6725432

9. Nalwadda G, Mirembe F, Byamugisha J, Faxelid E. Persistent high fertility in Uganda: young people recount obstacles and enabling factors to use of contraceptives. BMC Public Health. 2010;10: 530. doi:10.1186/1471-2458-10-530
10. Uganda Bureau of Statistics, ICF. Uganda Demographic and Health Survey 2022. Kampala: Uganda

Bureau of Statistics; 2023.

11. Nabugoomu J, Seruwagi GK, Hanning R. What can be done to reduce the prevalence of teen pregnancy in rural Eastern Uganda?: multi-stakeholder perceptions. Reprod Health. 2020;17: 134. doi:10.1186/s12978-020-00984-x
12. Tumwizere G, Bunani N, Muhumuza N, Uwera A, Kabagyenyi A. Non utilization of modern contraceptives among sexually active adolescent girls and young women aged 10–24 years in selected districts of Busoga region, Eastern Uganda: a cross-sectional study. Contracept Reprod

Med. 2025;10: 70. doi:10.1186/s40834-025-00407-x

13. Ministry of Health Uganda. Uganda Adolescent Health Policy and Service Standards. Kampala,

Uganda: Ministry of Health Uganda; 2012.

14. Jonas, Kim, Crutzen, Rik, Krumeich, Anja, Roman, Nicolette, van den Borne, Bart, Reddy, Priscilla. Healthcare workers’ beliefs, motivations and behaviours affecting adequate provision of sexual and reproductive healthcare services to adolescents in Cape Town, South Africa: a qualitative study.

BMC Health Services Research. 2017;17: 1–12.

15. Olajubu AO, Olowokere AE, Naanyu V. Barriers to Utilization of Sexual and Reproductive Health Services among Young People in Nigeria: A Qualitative Exploration Using the Socioecological

Model. Glob Qual Nurs Res. 2025;12: 23333936241310186. doi:10.1177/23333936241310186

16. Rosenstock IM. Historical Origins of the Health Belief Model. Health Educ Monogr. 1974;2: 328–

335. doi:10.1177/109019817400200403

17. Andersen, Ronald M. Revisiting the Behavioral Model and Access to Medical Care: Does It Matter?

Journal of Health and Social Behavior. 1995;36: 1–10.

18. Guest G, Bunce A, Johnson L. How Many Interviews Are Enough?: An Experiment with Data Saturation and Variability. Field Methods. 2006;18: 59–82. doi:10.1177/1525822X05279903
19. Lincoln YS, Guba EG, Pilotta JJ. Naturalistic inquiry. Int J Intercult Relat. 1985;9: 438–439. doi:10.1016/0147-1767(85)90062-8
20. Nowell LS, Norris JM, White DE, Moules NJ. Thematic Analysis: Striving to Meet the

Trustworthiness Criteria. Int J Qual Methods. 2017;16: 1609406917733847.

doi:10.1177/1609406917733847

21. Braun V, Clarke V. Using thematic analysis in psychology. Qual Res Psychol. 2006;3: 77–101. doi:10.1191/1478088706qp063oa
22. Tong A, Sainsbury P, Craig J. Consolidated criteria for reporting qualitative research (COREQ): a 32item checklist for interviews and focus groups. Int J Qual Health Care. 2007;19: 349–357. doi:10.1093/intqhc/mzm042
23. Klu D, Gyapong M, Agordoh PD, Azagba C, Acquah E, Doegah P, et al. Adolescent perception of sexual and reproductive health rights and access to reproductive health information and services in

Adaklu district of the Volta Region, Ghana. BMC Health Serv Res. 2023;23: 1456.

doi:10.1186/s12913-023-10447-1

24. Mbachu CO, Agu IC, Obayi C, Eze I, Ezumah N, Onwujekwe O. Beliefs and misconceptions about contraception and condom use among adolescents in south-east Nigeria. Reprod Health. 2021;18:

7. doi:10.1186/s12978-020-01062-y

25. Bronfenbrenner U. Ecology of Human Development: Experiments by Nature and Design.

Cambridge: Harvard University Press; 2009.

26. Athumani KF, Mboineki JF. Healthcare facility factors associated with health-seeking behavior among secondary school students in the Dodoma region: an analytical cross-sectional study. Front

Public Health. 2025;13: 1457318. doi:10.3389/fpubh.2025.1457318

27. Denno DM, Hoopes AJ, Chandra-Mouli V. Effective Strategies to Provide Adolescent Sexual and

Reproductive Health Services and to Increase Demand and Community Support. J Adolesc Health.

2015;56: S22–S41. doi:10.1016/j.jadohealth.2014.09.012

587 28.	Ninsiima LR, Chiumia IK, Ndejjo R. Factors influencing access to and utilisation of youth-friendly
588 sexual and reproductive health services in sub-Saharan Africa: a systematic review. Reprod Health. 589	2021;18: 135. doi:10.1186/s12978-021-01183-y

590

Revised Manuscript with Track Changes

**Stakeholder perspectives on adolescent sexual and reproductive**

1

**health service**

**~~utilisation~~**

**utilization**

**in Uganda**

**’**

**s Busoga region: A**

2

**qualitative study**

3

Noah Nyende¹*

[9](https://orcid.org/0009-0001-6342-4113)

[https://orcid.org/000](https://orcid.org/0009-0001-6342-4113)

[-](https://orcid.org/0009-0001-6342-4113)

[1](https://orcid.org/0009-0001-6342-4113)

[000](https://orcid.org/0009-0001-6342-4113)

[-](https://orcid.org/0009-0001-6342-4113)

[2](https://orcid.org/0009-0001-6342-4113)

[634](https://orcid.org/0009-0001-6342-4113)

[-](https://orcid.org/0009-0001-6342-4113)

[411](https://orcid.org/0009-0001-6342-4113)

[3](https://orcid.org/0009-0001-6342-4113)

Justine Nanteza², Frank Kiyingi¹, Miph

[,](https://orcid.org/0009-0001-6342-4113)

4

Musoke¹

5

**~~Affiliations~~**

6

1

~~¹~~

Nkumba University

School of Sciences

, Entebbe, Uganda

7

2

8

~~²~~

Makerere University

School of Public

Health, Kampala, Uganda

9

**~~Corresponding author~~**

10

~~Noah Nyende~~

11

*

Corresponding author

12

~~Email:~~

E

-

mail

:

rnnyende2002@yahoo.com

13

**Abstract**

14

**Background**

15

Adolescents in low

-

and middle

-

income countries face high rates of unintended pregnancy,

16

sexually transmitted infections (STIs

)

, and unmet sexual and reproductive health (SRH) needs. In

17

**Field**

**Code**

**Changed**

**Formatted:**

Superscript

**Formatted:**

Superscript

**Field**

**Code**

**Changed**

**Formatted:**

Normal

(

Web),

Space

Before:

0

pt,

After:

0

pt

Uganda, 24% of girls aged 15–19 years have begun childbearing, yet modern contraceptive prevalence among adolescents remains low (15%). These challenges are particularly severe in the Busoga region, where sociocultural norms, poverty, and limited health system capacity hinder access to adolescent-friendly SRH services. Despite national guidelines, utilization remains low. Few studies have triangulated perspectives from adolescents, health providers, and community/religious leaders. We explored stakeholder views to provide an integrated understanding of factors influencing adolescent SRH service utilization in Busoga, Uganda.~~Adolescents in low- and middle-income countries continue to experience high rates of~~ ~~unintended pregnancy, sexually transmitted infections, and unmet sexual and reproductive health~~

~~(SRH) needs. In Uganda, these challenges are exacerbated by sociocultural norms,~~ ~~misconceptions, and structural barriers that restrict adolescents’ access to suitable services.~~

**Formatted:**

Line

spacing:

Double

## ~~Objective~~

~~This study explored the perspectives of adolescents, community leaders, and health providers on~~ ~~adolescent SRH service utilisation in the Busoga Region of Eastern Uganda, to identify barriers,~~ ~~facilitators, and contextually appropriate strategies for improving adolescent-friendly service~~ ~~delivery.~~

**Methods**

~~An~~ An exploratory qualitative study was conducted in Iganga and Bugweri districts. Four focus group discussions with 38 adolescents (13–19 years), stratified by sex, age group, and residence, and 12 key informant interviews with health providers and community leaders were conducted. Adolescents were recruited through peer networks, while key informants were purposively selected. Data were collected (November–December 2024), transcribed verbatim, and analyzed

inductively using thematic analysis with NVivo 14. Ethical approval and informed

41

consent/assent were obtained.

~~exp~~

~~loratory quali~~

~~tative study was conducted~~

~~using focus group~~

42

~~discussions (FGDs) and key informant interviews (KIIs). Thirty~~

~~-~~

~~nine adolescents aged 13~~

~~–~~

~~19~~

43

~~years participated in four FGDs stratified by sex and residence (urban/rural). Twelve key~~

44

~~informants~~

~~, incl~~

~~uding~~

~~health providers, religious leaders, and cultural leaders~~

~~,~~

~~were purposively~~

45

~~selected.~~

46

~~Data were collected~~

~~between~~

~~November~~

~~and December~~

~~2024~~

~~using semi~~

~~-~~

~~structured guides, audio~~

~~-~~

47

~~recorded~~

~~, transcribed verbatim, and~~

~~analysed~~

~~inductively using Braun and~~

~~Clarke’s thematic~~

48

~~analysis framework with support from NVivo 14.~~

49

**Results**

50

Adolescents, health providers, and community stakeholders described SRH service utilization as

51

shaped by adolescent motivation alongside persistent barriers. Adolescents showed strong

52

preventive intent but uptake was limited by misconceptions about contrac

eption, fears of

53

infertility, long distances, lack of privacy, negative provider attitudes, and commodity stock

-

outs.

54

Providers and community leaders

echoed

concerns around misinformation, limited parental

55

guidance, moral norms, and structural constraints.

All stakeholders converged on the need for

56

confidential, accessible, and peer

-

and community

-

based adolescent SRH services.

~~Five~~

~~major~~

57

~~themes emerged: (1) adolescents’ positive orientation toward SRH prevention amid sexual self~~

~~-~~

58

~~discovery; (2) persistent mi~~

~~sconceptions and fears surrounding contraception; (3) access barriers~~

59

~~related to distance, privacy, and provider attitudes; (4) strong preferences for free, confidential,~~

60

~~peer~~

~~-~~

~~informed, and community~~

~~-~~

~~based SRH services; and (5) sociocultural and system~~

~~-~~

~~lev~~

~~el~~

61

~~constraints, including early marriage norms, religious prohibitions, poverty, and commodity~~

62

**Formatted:**

Justified

**Formatted:**

Font:

)

(

Default

Times

New

Roman,

12

pt

**Formatted:**

Font:

(

Default

)

Times

New

Roman,

12

pt

[12]. interventions. This integrated lens complements single-perspective studies and responds to calls for multi-level approaches to adolescent SRH in SSA [4,15]. ~~design of locally responsive, adolescent-friendly interventions.~~

pregnancy and HIV

4]

[3

,

.

Kenyan and Ethiopian studies, for example, identify transport costs,

judgmental provider attitudes, restrictive cultural and gender norm

s, fear of stigma, and lack of

privacy as recurring obstacles

~~5]~~

~~,~~

~~[3~~

6]

,

[5

.

~~[5~~

~~,6]~~

~~structural barriers such as stigma, misconceptions, and limited access contribute to low~~

~~utilisation~~

~~of SRH care, and persistently high rates of teenage pregnancy and HIV~~

~~[~~

~~4~~

~~,~~

~~3~~

~~]~~

~~. Kenyan~~

~~and Ethiopian studies, for example, highlight transport costs, judgmental providers, and~~

~~restrictive cultural norms as key barriers [~~

~~4~~

~~,~~

~~5~~

~~].~~

~~In~~

In

Uganda, adolescent SRH indicators remain concerning. Early sexual debut, low

contraceptive uptake, and high rates of adolescent pregnancy persist

[7

–

9]

.

~~, especially in~~

~~socioeconomically disadvantaged regions such as Busoga in Eastern Uganda~~

~~[~~

~~6~~

~~,~~

~~7~~

~~,~~

~~8~~

~~.~~

~~]~~

Uganda’s

adolescent pregnancy rate (teenage childbearing) is approximately 24% among girls

aged 15

–

19

years, HIV prevalence among 15

–

19

-

year

-

olds is 2.1%, and modern contraceptive use among

15

–

19

-

year

-

olds is only 15

[10]

~~}~~

~~Citation~~

~~{~~

~~Uganda’~~

~~s adolescent pregnancy rate is 24%, HIV~~

~~prevalence among 15~~

~~–~~

~~19~~

~~-~~

~~year~~

~~-~~

~~olds is 2.1%, and modern contraceptive use is only 15%~~

~~[~~

~~3~~

~~]~~

. The

Busoga Region is particularly affected because of poverty, early marriage norms, and limited

services

[11]

~~[~~

~~9~~

~~]~~

.

Recent studies in the region confirm high non

-

utilization of modern

contraceptives and SRH services, influenced b

y lack of confidence in discussing contraception,

perceived inaccessibility of services, and limited interaction with community health workers

Despite nationa

l adolescent health guidelines

[13]

~~[~~

~~10~~

~~]~~

,

~~utilisation~~

utilization

remains low due to

stigma, lack of privacy, and misinformation

~~[~~

~~9~~

~~,~~

~~8~~

~~]~~

,

9]

[8

.

~~;~~

Yet, little is known about how multiple

stakeholders, including adolescents themselves, community and religious leaders, and healthcare

**Formatted:**

English

United

(

States)

**Formatted:**

Line

spacing:

Double

**Formatted:**

Default

Paragraph

Font

**Formatted:**

Default

Paragraph

Font

providers, perceive these barriers and potential solutions within the same sociocultural context.

Most exi

sting research has focused on adolescents alone or examined isolated health system

factors

[4,14]

. This study addresses that gap by qualitatively triangulating perspectives from

adolescents, community and religious leaders, and health providers

in the Busoga Region. This

multi

-

stakeholder approach provides an integrated understanding of how community norms,

service delivery practices, and adolescent preferences interact to shape SRH service utilization,

thereby offering more nuanced insights for

designing locally responsive, adolescent

-

friendly

~~yet, little is known about how multipl~~

~~e stakeholders, including adolescents, community leaders,~~

~~and healthcare~~

~~providers, perceive these barriers and potential solutions~~

~~.~~

~~This gap limits the~~

~~While existing research has generated~~

~~important insights, most studies focus on adolescents~~

~~alone or examine health system factors in isolation. There is limited qualitative evidence that~~

~~triangulates the perspectives of adolescents, community leaders, and health providers within the~~

~~same soci~~

~~ocultural context [~~

~~11~~

~~.~~

~~]~~

To address this gap

**,**

~~Such triangulation is essential for~~

~~understanding how community norms, service delivery practices, and adolescent preferences~~

~~interact to shape SRH service~~

~~utilisation~~

~~.~~

~~T~~

t

h

e present

~~is~~

study

explored

~~aimed to ex~~

~~plore~~

adolescents’ attitudes, perceptions, and

perspectives of community leaders and health providers. By examining convergences and

divergen

ces across stakeholder groups, the study sought to identify multilevel barriers and

facilitators that can inform the design of contextually appropriate,

adolescent

-

friendly SRH

**Formatted:**

English

United

(

States)

**Formatted:**

Normal

Web),

(

Space

Before:

0

pt,

After:

0

pt

**Formatted:**

Left,

Line

spacing:

single

**Formatted:**

Font:

Not

Bold

**Formatted:**

Left

preferences regarding SRH services in the Busoga Region of eastern Uganda, alongside the

interventions. The study was guided by the Health Belief Model [16] ~~[12]~~ and Andersen’s Behavioural Model ~~[13]~~[17] to understand how individual perceptions, enabling factors, and sociocultural contexts shape SRH service ~~utilisation~~utilization.

## Materials and Methods

**Study design**

An exploratory qualitative design was employed, guided by a constructivist- interpretivist paradigm. This approach was appropriate for examining how adolescents and key community stakeholders construct meaning about~~around~~ SRH service ~~utilisation~~utilization from~~based on~~ their lived experiences.

**Study setting**

**Formatted:**

Normal

(

Web),

Space

Before:

0

pt,

After:

0

pt

The study was conducted in Iganga and Bugweri districts in the Busoga Region of Eastern Uganda. The Busoga Region is characterised by a mix of rural and urban communities, varying levels of health service availability, and strong cultural and religious influences on adolescent behaviour. Community entry was facilitated through district health officials and local council leaders. The study was conducted between 20 November and 18 December 2024 at six rural (Namirali, Kiwerere, Bulubandi, Naitandu B, Bunalwenyi C, Makandwa) and six urban

(Kasokoso Central II/III, Nakavule Main, Busesa, Butende, Kagamba) sites in Iganga and Bugweri districts. The two districts were selected because they reflect contrasting health system contexts: Bugweri has limited resources and fragile health infrastructure, whereas Iganga is better resourced with stronger systems and staffing, ensuring representation of diverse conditions influencing adolescent SRH service access and utilization ~~The region is characterised by a mix of~~ ~~rural and urban communities, varying levels of health service availability, and strong cultural and~~ ~~religious influences on adolescent behaviour. Iganga District has relatively better health~~ ~~infrastructure, while Bugweri District is more resource-constrained. Community entry was~~ ~~facilitated through district health officials and local council leaders. The study was conducted~~ ~~between 20~~^~~th~~^ ~~November and 18~~^~~th~~^ ~~December, 2024, at six rural (Namirali, Kiwerere, Bulubandi,~~

~~Naitandu B, Bunalwenyi C, Makandwa) and six urban (Kasokoso Central II/III, Nakavule Main,~~ ~~Busesa, Butende, Kagamba) sites in Iganga and Bugweri districts. The two districts were~~ ~~selected because they reflect contrasting health system contexts: Bugweri has limited resources~~ ~~and fragile health infrastructure, whereas Iganga is better resourced with stronger systems and~~ ~~staffing, ensuring representation of diverse conditions influencing adolescent SRH service access~~ ~~and utilisation~~ in the region.

**Participants and sampling**

**Formatted:**

Line

spacing:

Double

Two categories of participants were included in this study: adolescents and key informants. Adolescents were purposively sampled to capture diverse perspectives on SRH service utilization across gender, age, and residential contexts. Specifically, younger female adolescents (13–17 years) and older male adolescents (17–19 years) were selected to reflect gendered patterns in SRH vulnerability and engagement. Younger females are often at earlier stages of sexual debut and face heightened risks related to early pregnancy and barriers to accessing services. At the same time, older males are more likely to be sexually active and engaged in SRH-related decision-making. The overlap at age 17 was intentional to capture transitional developmental experiences between mid- and late adolescence.

Adolescents were further stratified by residence (rural/urban) to account for contextual differences in access to SRH information and services. In total, 38 adolescents participated in and youth peer leaders. Inclusion criteria for adolescents were: aged 13–19 years, residence in the study area for at least six months, and willingness to participate. leaders. Religious leaders were drawn from established faith-based institutions (churches and mosques) and held formal roles (clergy, pastors, or imams). In contrast, cultural leaders were recognized traditional authorities (clan leaders and elders). Health providers contributed

four focus group discussions (FGDs), with rec

ruitment facilitated through community mobilisers

Key informants were selected using pur

posive sampling based on their roles, experience, and

knowledge of adolescent SRH. These included health providers (such as nurses and clinical

officers delivering SRH services) and community leaders, including religious and cultural

perspectives based on their direct experience delivering services to adolescents, while cultural

and religious leaders offered insight into the broader sociocultural context shaping adolescent

health

-

seeking behaviour.

A total of twelve key informant int

erviews (KIIs) were conducted.

~~participants were included.~~

~~Adolescents aged 13~~

~~–~~

~~19~~

~~years participated in four FGDs stratified by~~

~~convenience sampling~~

~~with support from community mobilisers and youth peer leaders. Twelve~~

~~key informants were purposively selected based on their roles and knowledge of adolescent~~

~~SRH, including healt~~

~~h providers, religious leaders, and cultural leaders.~~

~~Sampling followed~~

~~established qualitative r~~

~~es~~

~~earch guidance~~

~~[~~

~~1~~

~~4~~

~~]~~

[18]

,

with participant recruitment continuing until

and twelve KIIs.

~~and proceeded iteratively. Participant recruitment continued until further data~~

~~no longer added new patterns of meaning, which occurred after 12 key informant interviews and~~

**Formatted:**

Left

**Field**

**Code**

**Changed**

Sampling proceeded iteratively, guided by principles of qualitative inquiry ~~Two categories of~~

~~sex and residence (urban/rural). In total, thirty-nine adolescents were recruited using~~

no new patterns of meaning emerged from the data. Data saturation was reached after four FGDs

~~hospital); 6~~

~~community leaders~~

~~(3~~

~~cultural, 3~~

~~religious)~~

**Data collection**

199

Semi

-

structured interview guides were developed based

on prior literature and national

200

adolescent SRH frameworks

[13]

. The guides explored adolescents’ attitudes, perceptions,

201

preferences, and barriers to SRH service utilizat

ion. Tools were prepared in English, translated

202

into Lusoga, and pilot

-

tested

before

data collection.

Pilot testing supported the credibility of the

203

tools by ensuring

the clarity, cultural appropriateness, and relevance of the questions to the study

204

context

[19]

.

205

Four FGDs, each comprising 9

–

10

participants, were conducted with adolescents. Discussions

206

were held in Lusoga

,

in neutral community venues and lasted

60

–

minutes. Each session was

90

207

facilitated by a trained moderator of the same sex as participants, where possible, and supported

208

by a note

-

taker. No external observers were present. Moderation followed a semi

-

structured

209

guide, with probing and neutral phr

asing used to manage sensitive topics and encourage

210

participation.

Prolonged engagement during discussions and the use of probing techniques

211

enhanced credibility by allowing participants to express detailed and in

-

depth perspectives.

212

A total of twelve KIIs

were conducted with health providers and community leaders. Interviews

213

were conducted in either English or Lusoga, depending on participant preference.

The inclusion

214

**Formatted:**

Line

spacing:

Double

**Formatted:**

English

United

(

States)

of multiple participant categories enabled triangulation of perspectives, further strengt

hening the

215

credibility of the findings

[19]

.

216

All participants were approached through peer mobilization or community intermediaries,

217

provided with study information, and invited to participate voluntarily. A small number of

218

adolescents declined par

ticipation due to time constraints or discomfort discussing SRH in group

219

settings, while no refusals were recorded among key informants. All FGDs and KIIs were audio

-

220

recorded with consent, and detailed field notes were taken to complement the recordings.

T

he

221

combination of audio recordings and field notes supported confirmability by ensuring that

222

findings were grounded in participants’ accounts.

223

~~Semi~~

~~-~~

~~structured interview guides were developed based on prior literature and national~~

224

~~adolescent SRH frameworks.~~

~~Guides explored attitudes, perceptions, preferences, and barriers~~

225

~~related to SRH service~~

~~utilisation~~

~~. The tools were prepared in English, translated into Lusoga,~~

226

~~and pilot~~

~~-~~

~~tested before data collection. FGDs were conducted in neutral community venues to~~

227

~~enhance privacy and lasted between 60 and 90 minutes. KIIs were conducted in English or~~

228

~~Lusoga, depending on participant preference. All sessions were audio~~

~~-~~

~~recorded with consent, and~~

229

~~detailed field notes were taken.~~

230

**Data analysis**

231

Data collection and anal

ysis proceeded iteratively, with emerging insights informing subsequent

232

interviews.

Saturation

was assessed through regular team debriefs and concurrent review of

233

transcripts, and data collection ceased at four FGDs and twelve KIIs when no new themes

234

emerg

ed, categories were well developed, and additional interviews were unlikely to yield

235

**Formatted:**

English

United

(

States)

**Formatted:**

Left

**Formatted:**

Line

spacing:

Double

**Formatted:**

Font:

Not

Bold

meaningful insights.

Regular debriefings also contributed to dependability by providing

236

opportunities to review and refine emerging interpretations in a systematic manner

[20]

.

237

Audio recordings were transcribed verbatim and, where necessary, translate

d into English. Back

-

238

translation was undertaken for selected transcripts to ensure accuracy and preserve meaning. All

239

data were anonymized and stored on password

-

protected devices accessible only to the research

240

team, in line with ethical requirements.

Bac

k

-

translation and careful data management

241

procedures enhanced credibility and confirmability by preserving the authenticity and integrity

242

of the data.

243

Data were analyzed inductively using

Braun and Clarke’s reflexive thematic analysis

, following

244

six phases

:

familiarisation with the data, coding, generating initial themes, reviewing themes,

245

defining and naming themes, and producing the report

[21]

~~Audio recordings were transcribed~~

246

~~verbatim and, where necessary, translated into English.~~

~~Back~~

~~-~~

~~translation was undertaken for~~

247

~~selected transcripts to ensure accuracy and preserve meaning. Data were~~

~~analysed~~

~~inductively~~

248

~~using Braun and Clarke’s reflexive thematic analysis approach, following the six analytic phases~~

249

~~of familiarisation with the d~~

~~ata, coding, generating initial themes, reviewing themes, defining~~

250

~~and naming themes, and producing the report~~

~~[~~

~~1~~

~~5~~

~~]~~

.

Coding focused on participants’ accounts and

251

meanings rather than pre

-

defined categories. Two researchers engaged

independently with the

252

data during the coding process and subsequently discussed interpretations to refine codes and

253

themes through reflexive dialogue. NVivo version 14 software was used to support data

254

organisation and analytic transparency.

Independent coding and collaborative theme refinement

255

strengthened dependability by enhancing consistency in the analytic process, while the use of

256

NVivo supported transparency and confirmability of

the

findings

[21]

.

257

**Formatted:**

English

United

(

States)

**Formatted:**

Font:

Not

Bold

**Formatted:**

Highlight

**Formatted:**

English

(

United

States)

decisions, to enhance transparency and dependability of the research process. constraints influencing service ~~utilisation~~utilization.

The research team comprised public health and social science researchers with prior experience

in adolescent

~~sexual and reproductiv~~

~~e health~~

SRH

research in Uganda. While the researchers did

not have pre

-

existing relationships with participants, their professional backgrounds and

familiarity with SRH programming informed analytic interpretation. Reflexive journaling was

used throughout

data collection and analysis to document assumptions, positionality, and

evolving interpretations, and to minimise undue influence on coding and theme development.

Reflexive practices further supported confirmability by ensuring that interpretations remain

ed

grounded in the data rather than researcher predispositions

[19]

.

In addition, a comprehensive

audit trail was maintained, including transcripts, field notes, coding frameworks, and analytic

Analysis was guided by the

Health Belief Model

~~[~~

~~1~~

~~2~~

~~]~~

[16]

and Andersen’s Behavioural Model

of Health Services Use

[17]

~~[~~

~~1~~

~~3~~

~~]~~

, which

were applied analytically to

inform

data interpretation.

E

merg

ing

~~ent~~

codes and themes were examined in relation to model constructs, including

perceived susceptibility, perceived barriers, enabling resources, and sociocultural predisposing

factors. This analytic application supported the interpretation of how adolescents’

motivations to

protect their

~~sexual and reproductive health~~

SRH

interacted with structural and contextual

The study was approved by

the Mildmay

Uganda Research Ethics Committee (MUREC

-

2024

-

and

451)

registered

by

the Uganda National Council for Science and Technology (SS3374ES).

Written informed consent was secured from all adult participants

and from parents/guardians of

minors, with adolescents'

assent

. Participation was voluntary, and confidentiality and anonymity

**Field**

**Code**

**Changed**

**Field**

**Code**

**Changed**

**Formatted:**

Line

spacing:

Double

**Ethical considerations**

were maintained. Adolescents in FGDs received modest reimbursement for transport and time. Key informants received no compensation, as interviews were conducted at their workplaces or other convenient locations at no cost. We followed the consolidated criteria for reporting qualitative studies (COREQ) in reporting the findings [22]

~~Ethical approval was obtained from the Mildmay Uganda Research Ethics Committee (MUREC2024-451) and the Uganda National Council for Science and Technology (SS3374ES). Written~~ ~~informed consent was obtained from all adult participants and from parents or guardians of~~ ~~minors, with assent from adolescents. Participation was voluntary, and confidentiality and~~ ~~anonymity were maintained throughout the study.~~

**Results**

**Formatted:**

Normal,

Space

Before:

Auto,

After:

Auto

**Characteristics of the study participants**

A total of four FGDs were conducted across Bugweri and Iganga districts, stratified by sex and residence. The composition of the focus group discussions is shown in Table 1.

**Table 1. Composition of Focus Group Discussions (FGDs) by Site**

| **FGD No. Gender Residence/Site Mean Age Sc** 1 Male Bugweri Rural 17.9 | | **hooling Status No. of Participants**  Mostly out  |
| --- | --- | --- |
| 2 Female Bugweri Urban | 16.0 | Mostly in |
| 3 Male Iganga Urban | 18.0 | Mostly in  |
| 4 Female Iganga Rural | 15.2 | Mostly in |

Male participants were drawn from rural Bugweri and urban Iganga, while female participants were recruited from urban Bugweri and rural Iganga. The mean age ranged from 15.2 to 18.0

explained:

**~~reproductive health needs.~~**

**~~Health providers contributed perspectives based on direct service~~**

**~~delivery to adolescents, while cultural and religious leaders provided insight into the broader~~**

**~~sociocultural context shaping adolescent behaviour and health~~**

**~~-~~**

**~~seeking.~~**

**~~The qualitative analysi~~**

**~~s generated five interrelated themes reflecting adolescents~~**

**~~’~~**

**~~lived~~**

**~~experiences and the perspectives of community leaders and health providers~~**

**~~,~~**

**~~presented with~~**

**~~selected illustrative quotes~~**

**~~:~~**

**. Positive orientation toward SRH prevention amid sexual self**

**1**

**-**

**disco**

**very**

Adolescents demonstrated a proactive orientation toward seeking SRH services, driven by a clear

awareness of the risks associated with early sexual activity, including unintended pregnancies

and

STIs

such as HIV. Many participants linked SRH services

to protecting their future

aspirations, particularly continuing education and avoiding early marriage.

Female adolescents frequently emphasised the protective role of SRH services. One participant

*“It can help prevent early marriages. Some girl*

*s get pregnant*

*,*

*and then the family forces*

*them to marry the boy. But if you go for SRH services, you get advice early*

*,*

*and you can*

*avoid that kind of situation before it even starts.” (Female adolescent, FGD 2, rural*

*Iganga)*

Another girl highlighted the importance of knowledge and personal agency:

*“When we go to health workers, they educate us that if you don’t have money, you can*

*ask your parents. If they don’t have*

*it*

*, you should be patient because not everything*

**Formatted:**

Line

spacing:

Double

**Formatted:**

Font:

Italic

**Formatted:**

Indent:

Left:

0.5

",

Line

spacing:

Double

**Formatted:**

Font:

Italic

**Formatted:**

Font:

Italic

**Formatted:**

Line

spacing:

Double

**Formatted:**

Font:

Italic

**Formatted:**

Indent:

Left:

0.5

",

Line

spacing:

Double

**Formatted:**

Font:

Italic

*comes imme*

*diately. Adolescents need to be sharp. Even if a friend influences you, you*

342

*don’t just follow. A knowledgeable girl can then positively influence others.” (Female*

343

*adolescent, FGD 1, urban Bugweri)*

344

Male adolescents similarly recognised these benefits, often

stressing personal responsibility and

345

focus on school. One boy stated:

346

*“To prevent unintended pregnancies and infections like HIV. If you start having sex*

347

*without knowing what you’re doing, you can easily get a girl pregnant or catch diseases.*

348

*Seeking th*

*ese services teaches you how to protect yourself and stay safe so you don’t*

349

*carry heavy burdens while still young.” (Male adolescent, FGD 3, urban Iganga)*

350

Another male participant highlighted the practical value of services available locally:

351

*“It helps yo*

*u prevent contracting STIs like HIV, syphilis, and others. You get health*

352

*education at places*

*such as*

*the health centre, and they also teach you about using*

353

*condoms properly. That way, you protect yourself instead of just risking everything.”*

354

*(*

*Male adolesc*

*ent, FGD 4, rural Bugweri)*

355

This positive orientation was often connected to broader life goals, with participants describing

356

how

access to SRH services reduced worries about pregnancy or infection

and supported better

357

school performance. However, this sens

e of agency developed amid limited parental guidance.

358

Both health providers and religious leaders highlighted significant gaps in parent

–

child

359

communication regarding sexual health.

360

One health provider explained the situation clearly:

361

**Formatted:**

Line

spacing:

Double

**Formatted:**

Font:

Italic

**Formatted:**

Indent:

Left:

0.5

",

Line

spacing:

Double

**Formatted:**

Line

spacing:

Double

**Formatted:**

Font:

Italic

**Formatted:**

Indent:

Left:

",

0.5

Line

spacing:

Double

**Formatted:**

Font:

Italic

**Formatted:**

Line

spacing:

Double

Adolescents faced multiple, interrelated barriers that limited their access to and

utilization

of

381

SRH services

. These barriers operated at cognitive, structural, interpersonal, and sociocultural

382

levels.

383

***Misconceptions and fears surrounding contraception***

384

Despite strong motivation to prevent unintended pregnancy, many adolescents held widespread

385

misconceptions abou

t modern contraceptive methods. Both female and male participants

386

expressed deep fears that contraceptive use could cause long

-

term reproductive harm,

387

particularly infertility:

388

*“*

*Sometimes*

*,*

*I hear that family planning methods may cause you to become barren, and*

389

*that scares me*

*.” (Female adolescent, FGD 2, rural Iganga)*

390

A male participant echoed this concern:

391

*“Use of family planning by a woman who has never had children makes her sterile.”*

392

*(*

*M*

*ale adolescent, FGD 3, urban Iganga)*

393

Adolescents also shared additional misinformation about method safety and use:

394

*“We are told they affect women. They can get stuck in women, and that the lubricant in*

395

*condoms is dangerous.” (Male adolescent, FGD 3, urba*

*n Iganga)*

396

Health providers confirmed the persistence of such misconceptions, noting that adolescents

397

frequently worried about serious health consequences:

398

**Formatted:**

Font:

Italic

**Formatted:**

Font:

Italic

**Formatted:**

Indent:

Left:

",

0.5

Line

spacing:

Double

**Formatted:**

Line

spacing:

Double

**Formatted:**

Font:

Italic

**Formatted:**

Indent:

Left:

",

0.5

Line

spacing:

Double

**Formatted:**

Line

spacing:

Double

**Formatted:**

Font:

Italic

**Formatted:**

Indent:

Left:

",

0.5

Line

spacing:

Double

*“*

*Some fear that if they use family planning methods*

*,*

*they will never get pregnant when*

399

*they grow up.*

*Others believe family planning methods cause cancer, that they can lose*

400

*the uterus, or get fibroids. Because of these beliefs, some refuse to use family planning.*

*”*

401

*(*

*Health provider, KII, urban*

*Bugweri*

*)*

402

*“And another thing which we always hear from them is that family planning causes*

403

*cancer.” (Health provider, KII, urban Iganga)*

404

Religious perspectives further shaped normative attitudes toward contraception. A religious

405

leader explained:

406

*“Some Muslim schol*

*ars also permit the use of condoms, but about the use of*

407

*contraceptives, Islam does not recommend.” (Religious leader, KII, Bugweri)*

408

These fears of infertility, misinformation about health risks, and religiously influenced norms

409

often outweighed the percei

ved benefits of contraception and discouraged uptake.

410

***Structural, interpersonal, and sociocultural barriers***

411

Adolescents also encountered practical and relational obstacles when

seeking

services. Structural

412

challenges, particularly long distances to facili

ties, high costs, and long waiting times, were

413

frequently mentioned:

414

*“Government health facilities are far, and you can wait many hours before seeing a*

415

*health worker.” (Male adolescent, FGD 3, urban Iganga)*

416

A female adolescent added:

417

**Formatted:**

Indent:

Left:

",

0.5

Line

spacing:

Double

**Formatted:**

Font:

Italic

**Formatted:**

Font:

Italic

**Formatted:**

Line

spacing:

Double

**Formatted:**

Font:

Italic

**Formatted:**

Indent:

Left:

0.5

",

Line

spacing:

Double

**Formatted:**

Line

spacing:

Double

**Formatted:**

Font:

Italic

**Formatted:**

Font:

Italic

**Formatted:**

Indent:

Left:

0.5

",

Line

spacing:

Double

**Formatted:**

Line

spacing:

Double

challenge:

*“For example, here,*

*if you go at noon, you might not come back until 4 or 5 pm. So*

*,*

*I end*

*up thinking, why don’t I just stay at home or go somewhere else… instead of wasting time*

*at the health facility.” (Female adolescent, FGD 1, urban Bugweri)*

Privacy and confidentiality we

re major concerns. Participants noted that small or overcrowded

health centres made confidential counselling difficult. A health provider confirmed this

*“The place is not enough for the adolescents to acquire knowledge or counselling*

*because an*

*adolescent needs privacy. But according to this health center, as small as it*

*is, we don't have enough privacy for the adolescents.” (Health provider, KII, rural*

*Iganga)*

Negative provider attitudes further discouraged service use. Adolescents described ex

periences

of being judged, spoken to harshly, or belittled by health workers. One girl recounted:

*“*

*…a*

*nd the government health facilities are far… the health workers come late, and they*

*are arrogant and proud… you can even go back home without being attend*

*ed to.”*

*(*

*Female adolescent, FGD 2, rural Iganga*

*)*

Community leaders placed these experiences within broader social contexts of stigma and power

imbalances. A cultural leader observed:

*“They are despised, which in turn makes them feel inferior. This affects*

*their ability to*

*ask for what they want. This happens a lot at health facilities.” (Cultural leader, KII,*

*Bugweri)*

**Formatted:**

Font:

Italic

**Formatted:**

Indent:

Left:

",

0.5

Line

spacing:

Double

**Formatted:**

Font:

Italic

**Formatted:**

Line

spacing:

Double

**Formatted:**

Font:

Italic

**Formatted:**

Indent:

Left:

0.5

",

Line

spacing:

Double

**Formatted:**

Line

spacing:

Double

**Formatted:**

Font:

Italic

**Formatted:**

Indent:

Left:

",

0.5

Line

spacing:

Double

**Formatted:**

Line

spacing:

Double

**Formatted:**

Font:

Italic

**Formatted:**

Indent:

Left:

0.5

",

Line

spacing:

Double

A religious leader added that shyness and fear related to their young age also prevented many

438

adolescents from seeking care:

439

*“They are shy.*

*They fear going to health facilities because they are young.” (Religious*

440

*leader, KII, Bugweri)*

441

***Sociocultural and system***

***-***

***level constraints***

442

Sociocultural norms and systemic challenges further shaped adolescents’ access to SRH services.

443

Religious and cultur

al leaders emphasised norms around morality, sexuality, and marriage that

444

often influenced when and how adolescents could engage with services:

445

*“Religion can guide good conduct, but sometimes we marry off adolescents early, and*

446

*they miss opportunities.” (R*

*eligious leader, KII, Bugweri)*

447

*“Girls should provide for the god of the home before they get married...they should*

448

*respect their ancestral spirits who are their spiritual husbands.” (Cultural leader, KII,*

449

*Bugweri)*

450

At the same time, health system constraint

s restricted access to services. Providers highlighted

451

persistent challenges related to the availability of commodities:

452

*“Sometimes it is stockouts, especially for family planning commodities. If one comes and*

453

*does not find the method they want, they don’*

*t come back.” (Health provider, KII,*

454

*Bugweri)*

455

**Formatted:**

Line

spacing:

Double

**Formatted:**

Font:

Italic

**Formatted:**

Indent:

Left:

",

0.5

Line

spacing:

Double

**Formatted:**

Line

spacing:

Double

**Formatted:**

Font:

Italic

**Formatted:**

Indent:

Left:

",

0.5

Line

spacing:

Double

**Formatted:**

Line

spacing:

Double

**Formatted:**

Font:

Italic

**Formatted:**

Indent:

Left:

0.5

",

Line

spacing:

Double

readiness: noted: platforms:

Adolescents similarly recognised these limitations and expressed the need for improved service

*“They should stock health facilities with more drugs…” (Female adolescent, FGD 2,*

*Iganga)*

Institutional

religious practices were also identified as structural barriers. One religious leader

*“Rigidity of church procedures, such as testing for HIV first before marriage, is another*

*challenge.” (Religious leader, KII, Iganga)*

Overall, cognitive, structu

ral, interpersonal, sociocultural, and system

-

level barriers interacted to

constrain adolescents’

utilization of

SRH service

s.

**. Preferences for adolescent**

**3**

**-**

**friendly and community**

**-**

**based services**

In response to the multiple barriers described above, adoles

cents expressed strong preferences

for SRH services that were accessible, confidential, and tailored to their specific needs. Many

emphasised the importance of decentralised delivery through community

-

and school

-

based

*“They should be everywhere*

*—*

*community, school and facilities.” (Male adolescent, FGD*

*, urban Iganga*

*)*

*3*

*“They should come up with clubs and specific days of sensitisation.” (Male adolescent,*

*FGD 4, rural Bugweri)*

**Formatted:**

Line

spacing:

Double

**Formatted:**

Font:

Italic

**Formatted:**

Indent:

Left:

",

0.5

Line

spacing:

Double

**Formatted:**

Line

spacing:

Double

**Formatted:**

Font:

Italic

**Formatted:**

Indent:

Left:

",

0.5

Line

spacing:

Double

**Formatted:**

Line

spacing:

Double

**Formatted:**

Font:

Italic

**Formatted:**

Indent:

Left:

0.5

",

Line

spacing:

Double

Peer

-

led approaches were particularly valued. A female adolescent stated

:

475

*“During school holidays, services should come to the community and be led by youth*

476

*because we believe our peers.” (Female adolescent, FGD 2, rural Iganga)*

477

Health providers described ongoing efforts to reach adolescents through outreach activities,

478

schoo

l visits, and collaboration with village health teams (VHTs):

479

*“We always provide health education when we go for outreaches, when we go for school*

480

*health education, and also when we talk with the VHTs, tell them to inform them about*

481

*the importance of our*

*services at the health unit.” (Health provider, KII, Iganga)*

482

Community and religious leaders reported integrating adolescent guidance into existing social

483

and institutional structures, including religious gatherings and personalised counselling:

484

*“We conduct community meetings. We preach and guide on good conduct during weekly*

485

*prayers on Fridays.” (Religious leader, KII, Bugweri)*

486

*“We provide one*

*-*

*on*

*-*

*one guidance for adolescents on what should be done.” (Cultural*

487

*leader, KII, Bugweri)*

488

These findings

show both convergence and variation in how adolescent

-

friendly SRH services

489

are conceptualised across stakeholders. Adolescents emphasised accessibility, confidentiality,

490

and peer

-

based engagement, while providers focused on outreach

-

oriented service deli

very, and

491

community and religious leaders underscored moral guidance and behavioural regulation. Across

492

these perspectives, common barriers

,

including contraceptive misinformation, limited privacy,

493

**Formatted:**

Line

spacing:

Double

**Formatted:**

Font:

Italic

**Formatted:**

Indent:

Left:

0.5

",

Line

spacing:

Double

**Formatted:**

Line

spacing:

Double

**Formatted:**

Font:

Italic

**Formatted:**

Indent:

Left:

0.5

",

Line

spacing:

Double

**Formatted:**

Line

spacing:

Double

**Formatted:**

Font:

Italic

**Formatted:**

Indent:

Left:

0.5

",

Line

spacing:

Double

**Formatted:**

Line

spacing:

Double

long distances to facilities, negative provider attitudes, and sociocultural expectations, were consistently reported as shaping adolescents’ experiences with SRH services.

~~1. Positive orientation toward SRH prevention amid sexual self-discovery~~

~~Adolescents demonstrated a proactive approach to sexual and reproductive health (SRH), driven~~ ~~by clear recognition of the risks of early sexual activity, particularly unintended pregnancy and~~ ~~sexually transmitted infections, including HIV. In focus group discussions, participants~~ ~~emphasised the protective role of SRH services:~~ *~~“It helps us to prevent sexually transmitted~~ ~~infections and to plan for the future.” (Girl, FGD, Bugweri).~~*

~~Health providers corroborated this observation, noting that adolescents actively sought~~ ~~preventive measures due to specific fears:~~ *~~“Girls fear pregnancies and boys fear illnesses like~~ ~~HIV, so they look for condoms and counselling.” (Health provider, KII, Bugweri).~~*

~~This positive orientation toward prevention developed largely independently, in a context where~~ ~~parental guidance on sexual matters was perceived as limited or absent. As one health provider~~ ~~described:~~ *~~“First of all, what I know is that parents lost interest in their children, so the children~~ ~~are growing themselves by self-discovery to each and everything.” (Health provider, KII,~~ ~~Iganga).~~*

~~A religious leader similarly attributed adolescents' engagement with health services to parental~~ ~~negligence:~~ *~~“It is due to the negligence of the parents, as they do not guide them on when to start~~ ~~sexual issues until they realise their children are seeking services in health facilities.” (Religious~~ ~~leader, KII, Bugweri).~~*

~~Thus, amid perceived gaps in parental involvement, adolescents exercised agency through selfdiscovery and help-seeking to safeguard their health and future aspirations.~~

~~2. Persistent misconceptions and fears surrounding contraception~~

~~Despite the expressed interest in prevention, widespread misconceptions and negative beliefs~~ ~~about modern contraceptive methods persisted, often deterring adolescents from utilising sexual~~ ~~and reproductive health services. Participants frequently cited fears that contraception could~~ ~~cause infertility, cancer, or other long-term health complications:~~ *~~“Sometimes family planning~~ ~~methods may cause you to become barren.” (Girl, FGD, Iganga).~~*

*~~“Use of family planning by a woman who has never had children makes her sterile.” (Boy, FGD,~~ ~~Iganga).~~*

*~~“And another thing which we always hear from them is that family planning causes cancer.”~~ ~~(Health provider, KII, Iganga).~~*

~~Specific concerns were also raised about condoms:~~ *~~“We are told they affect women. They can get~~ ~~stuck in women, and that the lubricant in condoms is dangerous.” (Boy, FGD, Iganga).~~*

~~Religious perspectives further contributed to these negative views, with some faith leaders~~ ~~expressing disapproval of contraceptive use:~~ *~~“Some Muslim scholars also permit the use of~~ ~~condoms. About the use of contraceptives, Islam does not recommend.” (Religious leader, KII,~~*

*~~Bugweri).~~*

~~Health providers and community members noted that such myths were commonly reinforced~~ ~~within families and religious settings, framing contraception as morally inappropriate or risky,~~ ~~which in turn discouraged adolescent uptake of services.~~

~~3. Barriers related to distance, privacy, and provider attitudes~~

~~Despite adolescents' proactive interest in sexual and reproductive health services, multiple~~ ~~structural and interpersonal barriers hindered access. Distance and transport costs posed~~ ~~significant challenges, especially in rural areas:~~ *~~“Government health facilities are far, and you~~ ~~can wait many hours before seeing a health worker.” (Boy, FGD, Iganga).~~*

~~Privacy concerns were frequently highlighted, with participants noting that small or crowded~~ ~~facilities offered limited confidentiality, increasing the risk of being seen or recognised by~~ ~~community members:~~ *~~“The place is not enough for the adolescents to acquire knowledge or~~ ~~counselling because an adolescent needs privacy. But according to this health center, as small as~~ ~~it is, we don't have enough privacy for the adolescents.” (Health provider, KII, Iganga).~~*

~~Negative attitudes from health workers further deterred service use, with adolescents describing~~ ~~experiences of judgement, harsh treatment, or being made to feel inferior:~~ *~~“The health workers~~ ~~are tough. Sometimes we fear to open up.” (Girl, FGD, Iganga).~~*

~~Community leaders also recognised that social stigma, internalised inferiority, and age-based~~ ~~power imbalances within communities and health facilities fostered shyness and fear of~~ ~~judgement, which prevented young people from seeking care:~~ *~~“They are despised, which in turn~~ ~~makes them feel inferior. This affects their ability to ask for what they want. This happens a lot at~~ ~~health facilities.” (Cultural leader, KII, Bugweri).~~*

*~~“They are shy. They fear going to health facilities because they are young.” (Religious leader,~~ ~~KII, Bugweri).~~*

~~These combined geographical, infrastructural, and interpersonal barriers contributed to delayed~~ ~~care-seeking, reduced trust in health facilities, and reluctance to return for subsequent services.~~

~~4. Preferences for adolescent-friendly and community-based services~~

~~Adolescents consistently expressed a strong preference for sexual and reproductive health~~ ~~services that were free, confidential, accessible, and tailored to their needs, with a particular~~ ~~emphasis on delivery outside traditional health facilities, such as in communities, schools, or~~ ~~through outreach programmes.~~

~~Participants highlighted the value of community- and school-based approaches led by peers or~~ ~~adolescent-friendly providers:~~ *~~“During school holidays, services should come to the community~~ ~~and be led by youth because we believe our peers.” (Girl, FGD, Iganga).~~*

*~~“They should be everywhere, community, school and facilities.” (Boy, FGD, Iganga).~~*

*~~“They should come up with clubs and specific days of sensitisation.” (Boy, FGD, Bugweri).~~*

~~Health providers described existing efforts to reach adolescents through outreaches, school visits,~~ ~~and collaboration with village health teams (VHTs):~~ *~~“We always provide health education when~~ ~~we go for outreaches, when we go for school health education and also when we talk with the~~*

*~~VHTs, tell them toinform them about the importance of our services at the health unit.” (Health~~ ~~provider, KII, Iganga).~~*

~~Community and religious leaders also contributed by integrating guidance into existing~~ ~~structures, such as religious gatherings and one-on-one counselling:~~ *~~“We conduct community~~ ~~meetings. We preach and guide on good conduct during weekly prayers on Fridays.” (Religious~~ ~~leader, KII, Bugweri).~~*

*~~“We provide one-on-one guidance for adolescents on what should be done.” (Cultural leader,~~ ~~KII, Bugweri).~~*

~~Overall, adolescents and stakeholders favoured decentralised, peer-involved, and communityembedded models that reduced barriers related to distance, stigma, and discomfort, thereby~~ ~~increasing the likelihood of service uptake.~~

~~5. Sociocultural and system-level constraints~~

~~Sociocultural norms and system-level challenges significantly constrained adolescents' access to~~ ~~sexual and reproductive health (SRH) services. Religious and cultural expectations often~~ ~~promoted early marriage or imposed restrictions that limited open engagement with SRH issues:~~ *~~“Religion can guide good conduct, but sometimes we marry off adolescents early, and they miss~~ ~~opportunities.” (Religious leader, KII, Bugweri).~~*

*~~“Girls should provide for the god of the home before they get married...they should respect their~~ ~~ancestral spirits who are their spiritual husbands.” (Cultural leader, KII, Bugweri).~~*

~~Religious practices further complicated access through rigid procedures: “~~*~~Rigidity of church~~ ~~procedures, such as testing for HIV first before marriage, is another challenge.” (Religious~~ ~~leader, KII, Iganga).~~*

~~At the health system level, frequent shortages of commodities and inadequate staffing~~ ~~undermined service reliability and trust:~~ *~~“They should stock health facilities with more drugs,~~ ~~and health workers are also few.” (Girl, FGD, Iganga).~~*

*~~“Sometimes it is stockouts, especially for family planning commodities. If one comes and does~~ ~~not find the method they want, they don’t come back.” (Health provider, KII, Bugweri).~~*

~~These intertwined sociocultural pressures and systemic deficiencies, ranging from normative~~ ~~expectations around marriage and spirituality to persistent resource gaps, collectively restricted~~ ~~adolescents' ability to exercise their agency in seeking and utilising SRH services fully.~~

## Discussion

**Formatted:**

Justified,

Line

spacing:

Double

This study demonstrates that adolescents in the Busoga Region are motivated to protect their sexual and reproductive health, yet this motivation is frequently constrained by intersecting sociocultural and structural barriers. Analytically applying the Health Belief Model [16] ~~[12]~~ and Andersen’s Behavioural Model [17] ~~13]~~ illustrates how strong perceived susceptibility to pregnancy and infection coexists with substantial perceived barriers, limited enabling resources, and restrictive community norms, resulting in delayed or foregone service ~~utilisation~~utilization.

Consistent with previous studies from Uganda and other East African settings, misconceptions about contraception, stigma, confidentiality concerns, and unfriendly provider attitudes emerged as major deterrents to service use [3–9,14,15]. These findings resonate strongly with broader evidence across SSA. A qualitative systematic review synthesizing studies from multiple SSA countries identified similar multi-level barriers, including misconceptions about SRH services, fear of stigma from family and community, low self-esteem, financial constraints, unsupportive family communication, judgmental provider attitudes, lack of privacy, and commodity shortages [4]. In Ethiopia, adolescents similarly reported fear of being seen by family or community members, restrictive social and gender norms, and inadequate integration of SRH information in health facilities as key obstacles [6]. Studies in Nigeria and Ghana have highlighted individuallevel barriers such as inadequate knowledge and poor attitudes toward services, compounded by strong social and religious norms that constrain open engagement with SRH issues [23,24].

Adolescents’ fears regarding infertility associated with contraceptive use mirror findings from across SSA and other low- and middle-income settings, where myths about long-term health complications (including infertility, cancer, or sterility) continue to shape SRH decision-making and deter uptake, particularly among nulliparous young people [4–6,9]~~Adolescents’ fears~~ ~~regarding infertility associated with contraceptive use mirror findings from sub-Saharan Africa,~~ ~~where myths continue to shape SRH decision-making [4,5,6,9]~~. Such concerns are often reinforced by community, religious, and peer networks.

**Formatted:**

Default

Paragraph

Font

~~.~~ The triangulation of perspectives in this study (from adolescents, community/religious leaders, and health providers) highlights that these barriers are reinforced at multiple levels (individual, familial, community, and health system) rather than arising solely from adolescents’ knowledge gaps [4,25]. ~~The triangulation of perspectives in this study highlights that these barriers are~~ ~~reinforced at multiple levels, including individual, familial, community, and health system, rather~~ ~~than arising solely from adolescents’ knowledge gaps [16].~~This multi-stakeholder lens

complements earlier single-perspective or facility-focused studies and aligns with socio-ecological approaches used in other SSA contexts [4,15].

~~The~~The findings can be interpreted through the lens of the ~~Health Belief Model (~~HBM~~)~~, which posits that ~~explains~~ health-related actions are~~s~~ shaped by perceived susceptibility, severity, benefits, barriers, cues to action, and self-efficacy [16]~~factors such as perceived susceptibility,~~ ~~severity, benefits, barriers, cues to action, and self-efficacy [12]~~. In this study, strong barriers (particularly stigma and fear of judgment from providers or the community) often outweighed perceived benefits of early service use, such as better symptom control or improved long-term outcomes. As the HBM predicts, when barriers exceed benefits, individuals tend to delay or avoid care. Comparable applications of the HBM in adolescent SRH research in Ethiopia and Ghana have shown that perceived social risks, including shame and fear of disclosure, frequently outweigh health benefits, leading to delayed utilization [6].~~{Citation}, participants often described~~ ~~strong barriers, especially stigma around the condition and fear of judgment from providers or~~ ~~their community. These concerns seemed to outweigh the perceived benefits of seeking help early,~~ ~~such as better symptom control or improved long-term outcomes. As the HBM predicts, when~~ ~~barriers feel greater than benefits, people tend to delay or avoid care altogether.~~

**Formatted:**

Normal

(

Web),

Justified,

Space

Before:

0

pt,

After:

0

pt

**Formatted:**

Default

Paragraph

Font

**Formatted:**

Justified,

Line

spacing:

Double

Andersen’s Behavioural Model complements this by distinguishing predisposing, enabling, and need factors [17]~~Andersen's Behavioural Model of Health Services Use adds a wider view by~~ ~~separating the influences on service use into predisposing, enabling, and need factors [13]~~. Predisposing elements, such as ~~like~~ sociocultural norms around gender or community attitudes toward the condition, appeared to discourage help-seeking from the outset. Concurrently~~start. At~~ ~~the same time~~, limited enabling resources (for example, money problems, transport issues, or scarce local services) hindered action even when the need was recognized. This combined and service availability.

perspective reveals how individual perceptions (HBM) interact with structural obstacles

(

Andersen’s model) to explain the low service utilization observed, consistent with patterns in

Tanzania and other

low and middle

-

income countries (

LMICs

)

where distance, cost, and service

availability interact with sociocultural norms

[26]

.

~~made it harder to act, even when participants clearly recognised their need for care. Together, the~~

~~two models show how personal percepti~~

~~ons (from the HBM) combine with structural obstacles~~

~~from Andersen's model) to explain the low~~

~~(~~

~~rates of service use we observed.~~

Importantly, evidence from SSA indicates that integrated, multi

-

component interventions

(

combining youth

-

friendly service provision, community engagement, and school

-

based

programs) are more effective than single

-

componen

t strategies for improving adolescent SRH

outcomes

[27,28]

.

Such a

pproaches address both psychological barriers through anti

-

stigma efforts

and improved communication, and practical access issues through enhanced resources, privacy,

Overall, the present findings underscore shared structural and

sociocultural challenges to

adolescent SRH service utilization across diverse settings while highlighting the value of context

-

specific, theory

-

informed interventions in the Busoga Region and similar East African contexts.

~~This combined perspective highlig~~

~~hts the complex reasons behind care~~

~~-~~

~~seeking decisions. It also~~

~~strengthens the case for interventions that address both sides: reducing psychological barriers~~

~~through anti~~

~~-~~

~~stigma efforts and improving practical access through better resources and service~~

~~a~~

~~vailability.~~

**Formatted:**

Normal

(

Web),

Justified,

Space

Before:

0

pt,

After:

0

pt

**Formatted:**

Font:

Not

Bold

**Formatted:**

Justified,

Line

spacing:

Double

**Formatted:**

Font:

Not

Bold

**Implications for policy and practice**

676

The results underscore the need for multi

-

component interventions that extend beyond facility

-

677

based service provision. At the health system level, there is a need to strengthen adolescent

-

678

friendly service delivery through

targeted training of health provid

ers in non

-

judgmental

679

communication, confidentiality, and youth

-

responsive care

, alongside the integration of adolescent

680

SRH competencies into routine in

-

service training and supervision frameworks. Health facilities

681

should also be supported to establish

d

edicated adolescent service corners, flexible service hours,

682

and privacy

-

enhancing infrastructure

to improve acceptability and

utilization

.

683

Expanding

community

-

and school

-

based outreach programmes

, including the use of trained peer

684

educators and collabora

tion with VHTs, can improve accessibility and trust in SRH services. In

685

addition, structured

community engagement strategies

are needed to involve parents, religious

686

leaders, and cultural gatekeepers in addressing misconceptions about contraception and pro

moting

687

supportive norms around adolescent SRH. This may include dialogue forums, faith

-

based

688

sensitisation initiatives, and parent

–

adolescent communication programmes.

689

To address persistent system barriers, policymakers should prioritise

strengthening supp

ly chain

690

management systems to ensure consistent availability of contraceptives and essential SRH

691

commodities

, particularly in rural facilities. Finally, integrating adolescent SRH indicators into

692

routine monitoring systems and strengthening accountability

mechanisms can support the

693

sustained delivery of responsive and equitable services.

694

~~The results underscore the need for multi~~

~~-~~

~~component interventions that extend beyond facility~~

~~-~~

695

~~based service provision. Strengthening~~

~~adolescent~~

~~-~~

~~friendly service~~

~~environments, expanding~~

696

**Formatted:**

Justified,

Line

spacing:

Double

**Formatted:**

Font:

Not

Bold

**Formatted:**

Font:

Not

Bold

**Formatted:**

Font:

Not

Bold

**Formatted:**

Font:

Not

Bold

**Formatted:**

Font:

Not

Bold

~~community- and school-based outreach, engaging parents and faith leaders in addressing~~ ~~misinformation, improving provider communication skills, and ensuring reliable commodity~~ ~~supply are critical steps supported by both participants’ perspectives and existing evidence.~~

## Strengths and limitations

A key strength of this study is the triangulation of perspectives from adolescents, community leaders, and health providers, which enhances credibility and contextual depth. However, the study was limited to two districts within the Busoga Region, potentially limiting transferability. While the findings offer insights that may be relevant to other similar low-resource settings with comparable sociocultural norms, economic challenges, and health system constraints, they are primarily grounded in the specific context of the Busoga region. Social desirability bias and challenges related to translation may also have influenced findings.

**Formatted:**

Normal

(

Web),

Space

Before:

0

pt,

After:

0

pt

**Formatted:**

Line

spacing:

Double

## Conclusion

This qualitative study demonstrates that adolescents in the Busoga Region of eastern Uganda possess motivation to protect their SRH, but face persistent sociocultural, structural, and health system barriers that constrain service utilization. Addressing misinformation, stigma, confidentiality concerns, and negative provider attitudes, alongside expanding adolescentfriendly and community-based service delivery, remains essential for improving adolescent SRH outcomes. In particular, decentralisation of SRH services through community- and school-based platforms, outreach programmes, and peer-led approaches represents a critical strategy for overcoming the distance, access, and confidentiality barriers identified in this study. Such approaches warrant careful adaptation to local contexts rather than direct replication.

~~This qualitative study~~

~~demonstrates~~

~~that adolescents in the Busoga Region possess motivation to~~

718

~~protect their sexual and reproductive health but face persistent sociocultural, structural, and~~

719

~~health system barriers that constrain service~~

~~utilisation~~

~~. Addressing misinformation, stigma,~~

720

~~confidentiality concerns, and provider attitudes, while expanding~~

~~adolescent~~

~~-~~

~~friendly and~~

721

~~community~~

~~-~~

~~based service delivery, remains essential for improving adolescent SRH outcomes in~~

722

~~similar low~~

~~-~~

~~resource sett~~

~~ings~~

~~.~~

723

**Data**

**a**

**~~A~~**

**vailability**

**s**

**~~S~~**

**tatement**

724

All de

-

identified focus group discussion (FGD) and key informant interview (KII) transcripts

725

from this study are publicly available in the Zenodo repository at DOI:

726

/zenodo.19708023

(

10.5281

[https://doi.org/10.5281/zenodo.1970802](https://doi.org/10.5281/zenodo.19708023)

[3](https://doi.org/10.5281/zenodo.19708023)

. Transcripts have been

[)](https://doi.org/10.5281/zenodo.19708023)

727

anonymized following BMJ (2010) guidelines, with all direct identifiers removed.

728

~~De~~

~~-~~

~~identified qualitative transcripts are~~

~~available from the corresponding author upon reasonable~~

729

~~request and subject to approval by the relevant ethics committees, due to the sensitive nature of~~

730

~~the data and the potential risk of participant identification.~~

731

**Acknowledgements**

732

We are

~~I am~~

deeply

grateful to the adolescents who participated in this study

.

We

~~I~~

also appreciate

733

the research team for their invaluable contribution. Lastly, to

the District Health Offices of Iganga

734

and Bugweri,

for

their support and facilitation

of this research

.

735

**Formatted:**

Highlight

**Formatted:**

Font:

Default

(

)

Times

New

Roman,

12

pt

### 746 References

747	1.

748

749

750	2.

751

752

753

754	3.

755

756

757	4.

758

759

760

761	5.

762

763

764

6. Sidamo N, Kerbo A, Gidebo K, Wado YD. Exploring Barriers to Accessing Adolescents Sexual and Reproductive Health Services in South Ethiopia Regional State: A Phenomenological Study Using Levesque’s Framework. Adolesc Health Med Ther. 2024;Volume 15: 45–61.

doi:10.2147/AHMT.S455517

7. Atuyambe LM, Kibira SPS, Bukenya J, Muhumuza C, Apolot RR, Mulogo E. Understanding sexual and reproductive health needs of adolescents: evidence from a formative evaluation in Wakiso district, Uganda. Reprod Health. 2015;12: 35. doi:10.1186/s12978-015-0026-7
8. Akatukwasa C, Bajunirwe F, Nuwamanya S, Kansime N, Aheebwe E, Tamwesigire IK. Integration of

HIV-Sexual Reproductive Health Services for Young People and the Barriers at Public Health

Facilities in Mbarara Municipality, Southwestern Uganda: A Qualitative Assessment. Int J Reprod Med. 2019;2019: 1–11. doi:10.1155/2019/6725432

9. Nalwadda G, Mirembe F, Byamugisha J, Faxelid E. Persistent high fertility in Uganda: young people recount obstacles and enabling factors to use of contraceptives. BMC Public Health. 2010;10: 530. doi:10.1186/1471-2458-10-530
10. Uganda Bureau of Statistics, ICF. Uganda Demographic and Health Survey 2022. Kampala: Uganda Bureau of Statistics; 2023.
11. Nabugoomu J, Seruwagi GK, Hanning R. What can be done to reduce the prevalence of teen pregnancy in rural Eastern Uganda?: multi-stakeholder perceptions. Reprod Health. 2020;17: 134. doi:10.1186/s12978-020-00984-x
12. Tumwizere G, Bunani N, Muhumuza N, Uwera A, Kabagyenyi A. Non utilization of modern contraceptives among sexually active adolescent girls and young women aged 10–24 years in selected districts of Busoga region, Eastern Uganda: a cross-sectional study. Contracept Reprod Med. 2025;10: 70. doi:10.1186/s40834-025-00407-x
13. Ministry of Health Uganda. Uganda Adolescent Health Policy and Service Standards. Kampala, Uganda: Ministry of Health Uganda; 2012.
14. Jonas, Kim, Crutzen, Rik, Krumeich, Anja, Roman, Nicolette, van den Borne, Bart, Reddy, Priscilla. Healthcare workers’ beliefs, motivations and behaviours affecting adequate provision of sexual and reproductive healthcare services to adolescents in Cape Town, South Africa: a qualitative study. BMC Health Services Research. 2017;17: 1–12.
15. Olajubu AO, Olowokere AE, Naanyu V. Barriers to Utilization of Sexual and Reproductive Health Services among Young People in Nigeria: A Qualitative Exploration Using the Socioecological

Model. Glob Qual Nurs Res. 2025;12: 23333936241310186. doi:10.1177/23333936241310186

16. Rosenstock IM. Historical Origins of the Health Belief Model. Health Educ Monogr. 1974;2: 328–

335. doi:10.1177/109019817400200403

17. Andersen, Ronald M. Revisiting the Behavioral Model and Access to Medical Care: Does It Matter?

Journal of Health and Social Behavior. 1995;36: 1–10.

18. Guest G, Bunce A, Johnson L. How Many Interviews Are Enough?: An Experiment with Data Saturation and Variability. Field Methods. 2006;18: 59–82. doi:10.1177/1525822X05279903
19. Lincoln YS, Guba EG, Pilotta JJ. Naturalistic inquiry. Int J Intercult Relat. 1985;9: 438–439. doi:10.1016/0147-1767(85)90062-8
20. Nowell LS, Norris JM, White DE, Moules NJ. Thematic Analysis: Striving to Meet the Trustworthiness Criteria. Int J Qual Methods. 2017;16: 1609406917733847.

doi:10.1177/1609406917733847

21. Braun V, Clarke V. Using thematic analysis in psychology. Qual Res Psychol. 2006;3: 77–101. doi:10.1191/1478088706qp063oa
22. Tong A, Sainsbury P, Craig J. Consolidated criteria for reporting qualitative research (COREQ): a 32item checklist for interviews and focus groups. Int J Qual Health Care. 2007;19: 349–357. doi:10.1093/intqhc/mzm042
23. Klu D, Gyapong M, Agordoh PD, Azagba C, Acquah E, Doegah P, et al. Adolescent perception of sexual and reproductive health rights and access to reproductive health information and services in Adaklu district of the Volta Region, Ghana. BMC Health Serv Res. 2023;23: 1456.

doi:10.1186/s12913-023-10447-1

**Formatted:**

Indent:

Left:

0

",

Space

Before:

0

pt,

After:

8

pt,

Add

space

between

paragraphs

of

the

same

style

24. Mbachu CO, Agu IC, Obayi C, Eze I, Ezumah N, Onwujekwe O. Beliefs and misconceptions about contraception and condom use among adolescents in south-east Nigeria. Reprod Health. 2021;18:

7. doi:10.1186/s12978-020-01062-y

25. Bronfenbrenner U. Ecology of Human Development: Experiments by Nature and Design. Cambridge: Harvard University Press; 2009.
26. Athumani KF, Mboineki JF. Healthcare facility factors associated with health-seeking behavior among secondary school students in the Dodoma region: an analytical cross-sectional study. Front Public Health. 2025;13: 1457318. doi:10.3389/fpubh.2025.1457318
27. Denno DM, Hoopes AJ, Chandra-Mouli V. Effective Strategies to Provide Adolescent Sexual and Reproductive Health Services and to Increase Demand and Community Support. J Adolesc Health.

2015;56: S22–S41. doi:10.1016/j.jadohealth.2014.09.012

28. Ninsiima LR, Chiumia IK, Ndejjo R. Factors influencing access to and utilisation of youth-friendly sexual and reproductive health services in sub-Saharan Africa: a systematic review. Reprod Health. 2021;18: 135. doi:10.1186/s12978-021-01183-y

Response to Reviewers

**Response to Reviewers**

**Manuscript ID:** PONE-D-26-00996

**Title:** *Stakeholder perspectives on adolescent sexual and reproductive health service utilization in Uganda's Busoga region: A qualitative study* **Journal:** PLOS ONE

**Dear Editor and Reviewers,**

We sincerely thank you for your thorough, rigorous, and constructive review of our manuscript. Your detailed feedback has been invaluable in significantly strengthening the clarity, methodological transparency, analytical depth, and overall quality of our work. We have carefully revised the manuscript in response to all comments. Below, we provide a detailed, point-by-point response, with corresponding line references to the revised manuscript. **All major revisions and additions are highlighted in bold text** for ease of review.

# 1. PLOS ONE Formatting and Style

**Comment:** Ensure the manuscript complies with PLOS ONE formatting guidelines.

**Response:** The manuscript has been fully revised to comply with PLOS ONE formatting requirements, including structure, headings, referencing style, and file naming conventions. **The abstract now follows the structured format (Background, Methods, Results, Conclusion) as required.**

# 2–4. Data Availability and Open Data Policy

**Comment:** Clarify data sharing, restrictions, and repository deposition. (Reviewers #2,

#3, and #4 raised concerns about data availability.)

**Response:** We have substantially revised the data availability approach to align fully with PLOS ONE's open data policy:

- **A fully de-identified qualitative dataset (focus group discussions and key informant interviews) has been prepared following established anonymization standards (e.g., BMJ 2010).**
- **All direct identifiers (names of participants, specific village names, health facilities, dates, and institutional affiliations) have been removed or generalized to protect participant confidentiality.**
- **The dataset has been deposited in a public repository .**
- **The Data Availability Statement has been updated to include the repository link and accession number.**
- **We confirm that written informed consent (and assent for minors) explicitly included permission for the use of anonymized data in research outputs and public repositories.**
- **No potentially identifiable information remains in the deposited dataset.**

This ensures full compliance with PLOS ONE's requirement that data underlying findings be publicly accessible while preserving participant confidentiality.

**Revised text:** Data Availability Statement (Lines 487–490); Ethical Considerations (Lines 188–196).

# Point-by-Point Responses to Reviewer Comments

We have consolidated comments from **all four reviewers** and respond to each below.

## Abstract

**Comment 1 (Reviewers #1 and #2):** The abstract background is too general. It needs greater specificity, quantitative indicators, and a clearer rationale for the Busoga region and stakeholder approach.

**Response:** The abstract background has been completely revised to include:

- **Specific quantitative indicators: 24% adolescent childbearing rate, 15% modern contraceptive prevalence among adolescents (Uganda Demographic and Health Survey 2022).**
- **Named barriers: sociocultural norms, poverty, limited health system capacity.**
- **Clear justification: the Busoga region is disproportionately affected by these challenges, yet no study has triangulated perspectives from adolescents, providers, and community/religious leaders.**

**Revised text:** Abstract, Lines 13–20.

**Comment 2 (Reviewer #1):** Replace "suitable services" with a more precise term.

**Response:** "Suitable services" has been replaced with **"adolescent-friendly SRH services"** throughout the abstract and manuscript.

**Revised text:** Abstract, Line 17; also consistent throughout.

**Comment 3 (Reviewers #1 and #2):** The objective combines several aims, diluting clarity. It should more directly reflect the primary research purpose.

**Response:** The objective has been refined to focus on the **single primary aim: to explore stakeholder perspectives to provide an integrated understanding of factors influencing adolescent SRH service utilization.** The secondary elements (barriers, facilitators, strategies) now appear in the results and conclusion.

**Revised text:** Abstract, Lines 19–20.

**Comment 4 (Reviewer #1):** The abstract methods section is incomplete.

**Response:** The methods section has been expanded to include:

- **Study setting (Iganga and Bugweri districts, Busoga region).**
- **Recruitment approach: adolescents through peer networks; key informants purposively selected.**
- **A clear statement of ethical approval and consent procedures.**

**Revised text:** Abstract, Lines 22–28.

**Comment 5 (Reviewers #1, #2, and #3):** The results section lists themes descriptively but does not articulate the main analytical insight. The abstract and results should reflect the collapse of five themes into three.

**Response:** Thank you for this critical observation. We have substantially restructured the results to address this concern.

**First, the original five themes have been collapsed into three analytically coherent themes** that follow a clear logical progression from **motivation → barriers → solutions**:

| **Original Five Themes** | **Collapsed into Three Themes** |
| --- | --- |
| 1. Positive orientation toward SRH prevention amid sexual self-discovery | **Theme 1: Positive orientation toward SRH prevention amid sexual selfdiscovery** (motivation) |
| 2. Persistent misconceptions and fears surrounding contraception | ↓ |
| 3. Access barriers related to distance, privacy, and provider attitudes | **Theme 2: Barriers to SRH service utilization** (barriers) |
| 5. Sociocultural and system-level constraints | ↓ |
| 4. Strong preferences for free, confidential, peer-informed, and community-based SRH services | **Theme 3: Preferences for adolescent-friendly and community-based services** (solutions) |

**Second, the results section has been reframed to highlight the core analytical insight: adolescents are motivated to use SRH services, but this motivation is constrained by intersecting barriers, leading them to prefer alternative service models.** The three themes are now presented within this analytical narrative rather than as a simple list.

**Third, Theme 3 (Preferences) is now clearly articulated and highlighted in bold throughout the results section.** This theme captures adolescents' strong preferences for:

- **Free, confidential, and accessible services**
- **Community- and school-based delivery platforms**
- **Peer-led approaches (because "we believe our peers")**
- **Outreach programmes and integration with existing community structures**

**(e.g., village health teams, religious gatherings)**

**Revised text:** Abstract, Lines 30–36; Results, Lines 216–224 (theme overview) and Lines 359–381 (**Theme 3: Preferences for adolescent-friendly and community-based services**).

## Methods (Data Collection, Recruitment, and Transparency)

**Comment 6 (Reviewers #1, #2, and #3):** Expand the data collection/procedure section to include recruitment and sampling processes, saturation rationale, and data security. **Response:** The Methods section has been substantially expanded to include:

- **Detailed recruitment processes for adolescents (via community mobilisers and youth peer leaders) and key informants (purposively selected based on institutional roles).**
- **Explicit saturation rationale: data collection continued until no new patterns emerged, achieved after 4 FGDs and 12 KIIs, confirmed through team debriefing and concurrent transcript review.**
- **Data security procedures: anonymization, password-protected devices, restricted access to the research team.**

**Revised text:** Lines 99–162.

**Comment 7 (Reviewers #1, #2, and #3):** Explain how adolescents and key informants were identified and approached. Add inclusion/exclusion criteria, definitions of urban/rural residence, and whether any participants declined. **Reviewer #2 specifically noted that the sampling strategy needed clarification, and Reviewer #3 noted that convenience sampling was incorrectly stated.**

**Response:** This section has been significantly expanded. **We have corrected the sampling description: it is purposive sampling, not convenience sampling.**

- **Adolescents were purposively sampled to capture diverse perspectives across gender, age, and residential contexts. Recruitment was facilitated through community mobilisers and youth peer leaders, with outreach in communities.**
- **Inclusion criteria for adolescents: age 13–19 years, residence in the study area for at least six months, and willingness to participate. No exclusion criteria were applied beyond these.**
- **Key informants were purposively selected based on formal institutional roles (health providers: nurses and clinical officers; religious leaders: clergy, pastors, imams; cultural leaders: clan leaders and elders) and recognized community authority.**
- **A small number of adolescents declined participation due to time constraints or discomfort discussing SRH in group settings. No refusals were recorded among key informants.**

**Revised text:** Lines 110–146.

**Comment 8 (Reviewers #1 and #2):** Provide more operational detail for FGD procedures: group size, composition rules, presence of note-takers, language, and handling of sensitive topics.

**Response:** The following details have been added:

- **Each FGD comprised 9–10 participants.**
- **Groups were stratified by sex and residence. Age stratification was implicit: female groups were younger (13–17 years); male groups were older (17–19 years) to reflect developmental stages and facilitate open discussion.**
- **Each session was facilitated by a trained moderator of the same sex as participants. A note-taker was present; no external observers were in the room.**
- **All FGDs were conducted in Lusoga, the local language.**
- **Sensitive topics were managed using neutral phrasing, probing techniques, and reassurance of confidentiality. Participants were reminded that they could skip any question or withdraw at any time.**

**Revised text:** Lines 132–138.

**Comment 9 (Reviewers #1, #2, and #3):** Clarify how the team determined that sufficient saturation was reached for FGDs and KIIs.

**Response:** A clear saturation statement has been added:

- **Sampling proceeded iteratively, with regular team debriefings and concurrent review of transcripts.**
- **Saturation was considered reached when no new themes emerged, categories were well-developed, and additional interviews were unlikely to yield meaningful new insights.**
- **This occurred after 4 FGDs and 12 KIIs, at which point data collection ceased.**

**Revised text:** Lines 122–124 (sampling) and Lines 151–155 (analysis).

**Comment 10 (Reviewer #4):** Ensure trustworthiness parameters (credibility, dependability, confirmability, transferability) are explicitly stated within the context of the research. Cite relevant literature.

**Response:** Thank you for this important guidance. The Methods section has been revised to explicitly address all four trustworthiness criteria:

- **Credibility: achieved through prolonged engagement during discussions, probing techniques, triangulation of multiple stakeholder perspectives, and back-translation of selected transcripts (Lines 132–138, 151–155).**
- **Dependability: ensured through regular team debriefings, independent coding by two researchers, and maintenance of a comprehensive audit trail (Lines 151–162).**
- **Confirmability: supported by reflexive journaling, documentation of assumptions and positionality, and grounding interpretations in participant accounts rather than researcher predispositions (Lines 162–168).**
- **Transferability: acknowledged that findings are primarily applicable to the Busoga region (Lines 470–471).**

**Revised text:** Lines 132–138, 151–168, 470–471.

## Results Section

**Comment 11a (Reviewers #1 and #2):** No detailed description of participants is provided. A table summarizing participant characteristics is essential.

**Response:** **Two new tables have been added:**

- **Table 1: Composition of Focus Group Discussions (FGDs) by site, gender, mean age, schooling status, and number of participants.**
- **Table 2: Composition of Key Informant Interviews (KIIs) by participant group, gender, district, rural/urban location, and total participants.**

**Revised text:** Lines 201–212 (Tables 1 and 2).

**Comment 11b (Reviewers #1 and #2 – Summary table of themes and subthemes):** Reviewer #1 specifically noted: *"The authors may consider including a summary table of themes and subthemes to improve readability."* Reviewer #2 similarly suggested organizing the findings with a table.

**Response:** Thank you for this practical suggestion. **We have included Table 3 in the revised manuscript**, which provides a clear, at-a-glance summary of the three main themes and their corresponding sub-themes.

**This table directly addresses Reviewer #1's request for improved readability and Reviewer #2's suggestion for better organization of findings.** It appears immediately after the participant characteristics tables (Tables 1 and 2) and before the detailed thematic narrative, allowing readers to orient themselves before reading the full results.

**Revised text:** Lines 214–224 (Table 3).

**Comment 11c (Reviewers #1, #2, and #3):** Themes are presented descriptively; the analysis should explain how themes relate to each other. The collapse of five themes into three should be clearly shown.

**Response:** The results section has been restructured to present **three interconnected themes** that follow a clear analytical progression:

1. **Theme 1: Positive orientation toward SRH prevention amid sexual selfdiscovery** (motivation and agency).
2. **Theme 2: Barriers to SRH service utilization** (sub-themes: misconceptions, structural/interpersonal barriers, sociocultural/system constraints).
3. **Theme 3: Preferences for adolescent-friendly and community-based services** (solutions).

**An explicit analytical pathway has been added: adolescents show motivation (Theme 1) → but face barriers (Theme 2) → leading them to prefer alternative service models (Theme 3).**

**Within Theme 3, we now clearly articulate adolescents' specific preferences, including: free and confidential services; community- and school-based delivery platforms; peer-led approaches ("because we believe our peers"); outreach programmes; and integration with existing community structures such as village health teams and religious gatherings.**

**Revised text:** Lines 216–224 (overview), and throughout the Results section (Lines 233– 381).

**Comment 11d (Reviewers #1 and #2):** Quotes lack sufficient detail. Use a standardized format.

**Response:** All quotes have been standardized to the following format: ***(Quote text, Participant group, FGD/KII number, district, rural/urban).*** For example: *(Female adolescent, FGD 2, rural Iganga).* This has been applied consistently throughout the Results section.

**Revised text:** Throughout Lines 233–381.

**Comment 11e (Reviewer #3):** The analysis sometimes merges perspectives without clearly distinguishing them. Provide clearer differentiation between stakeholder groups.

**Response:** The analysis has been revised to clearly distinguish perspectives:

- **Adolescents: emphasize privacy, stigma, fear of judgment, and preferences for peer-led services.**
- **Health providers: highlight service capacity issues, stockouts, staffing shortages, and efforts to reach adolescents through outreach.**
- **Religious and cultural leaders: stress moral norms, early marriage practices, religious prohibitions on contraception, and the role of community guidance.**

**This differentiation is now explicit throughout the Results section.**

**Revised text:** Lines 233–381 (e.g., separate quotes and analysis for each stakeholder group within each theme).

**Comment 11f (Reviewers #1 and #3):** Results are largely descriptive; interpretive insight is needed. Add reflexivity and analytical interpretation.

**Response:** Interpretive commentary has been added throughout the Results section, including:

- **Parental silence: how limited parent–adolescent communication drives selfdirected learning and reliance on peer networks.**
- **Misinformation: how fears of infertility and cancer, reinforced by community and religious narratives, outweigh perceived benefits of contraception.**
- **Barrier interaction: how distance, privacy, provider attitudes, and stockouts combine to create compounded access failures.**
- **Preference logic: why adolescents prefer community-based and peer-led services as a direct response to stigma and confidentiality concerns (explicitly developed in Theme 3).**

**Revised text:** Lines 233–381 (interpretive statements added within each theme).

**Comment 11g (Reviewers #1 and #2):** The results section should briefly indicate whether saturation was achieved.

**Response:** A saturation statement has been added to the Results section:

**Revised text:** Lines 122-124, 151–155.

**Comment 11h (Reviewer #3):** The integration of systems and cultural factors (formerly Theme 5) reads as fragmented. Better highlight interactions.

**Response:** Theme 2 (which now incorporates the former Theme 5) has been substantially revised to show the **interaction between religion, early marriage norms, poverty, and health system weaknesses.** The sub-theme now explicitly demonstrates how:

- **Religious norms and early marriage practices constrain adolescents' agency.**
- **Health system stockouts and staffing shortages compound these cultural barriers.**
- **The combination of normative expectations and service gaps leads to delayed or foregone care.**

**Revised text:** Lines 277–358 (previously Theme 5, now integrated into Theme 2).

## Discussion

**Comment 12 (Reviewers #2, #3, and #4):** The discussion would benefit from broader comparison with studies from other sub-Saharan African countries and similar settings outside Africa. Reviewer #4 specifically noted that only one study from South Africa was cited.

**Response:** The discussion has been substantially expanded to include comparisons with studies from **Ethiopia, Nigeria, Ghana, Kenya, Tanzania, and other sub-Saharan African countries.** New references have been added (References 4, 5, 6, 23, 24, 26). The discussion now explicitly situates our findings within the broader SSA evidence base while highlighting the unique contribution of multi-stakeholder triangulation in the Busoga context.

**Revised text:** Lines 400–445.

**Comment 13 (Reviewer #2):** Some sections explaining the theoretical models are relatively long and could be more directly connected to the study findings.

**Response:** The theoretical application sections have been shortened and more tightly integrated with specific findings from each theme. Direct links between HBM constructs (perceived barriers, perceived benefits) and Andersen's model (predisposing, enabling, need factors) are now explicitly mapped to participant quotes.

**Revised text:** Lines 420–445.

## Implications for Policy and Practice

**Comment 14 (Reviewer #2):** Provide more concrete recommendations for health systems, such as training of providers, strengthening youth-friendly services, or community engagement strategies.

**Response:** The Implications section has been expanded to include **specific, actionable recommendations:**

- **Health system level: targeted provider training in non-judgmental communication, dedicated adolescent service corners, flexible service hours, and privacy-enhancing infrastructure.**
- **Community level: structured engagement with parents, religious leaders, and cultural gatekeepers through dialogue forums and faith-based sensitization initiatives.**
- **School level: peer education programmes and integration of SRH information into existing curricula.**
- **Supply chain: strengthening commodity supply chain management to reduce stockouts, particularly for contraceptives in rural facilities.**

**Revised text:** Lines 451–467.

## Strengths and Limitations

**Comment 15 (Reviewer #2):** The limitations section could also mention possible selection bias due to sampling and limitations in generalizability beyond the study region.

**Response:** The limitations section has been expanded to explicitly acknowledge:

- **Geographic scope** – limited to two districts in Busoga, limiting transferability
- **Context specificity** – findings primarily grounded in Busoga region
- **Social desirability bias** – participants may have given socially acceptable responses
- **Translation challenges** – potential loss of nuance in translation from Lusoga to English

**Revised text:** Lines 470–475.

## Conclusion

**Comment 16 (Reviewers #1 and #2):** The conclusion overgeneralizes findings to "similar low-resource settings" without sufficient caution. Emphasize specific actionable recommendations.

**Response:** The conclusion has been revised to:

- **Emphasize that the findings are primarily applicable to the Busoga region of eastern Uganda.**
- **Note cautiously that the insights may be relevant to other contexts with comparable sociocultural norms, economic challenges, and health system constraints, but that direct replication requires contextual adaptation.**
- **Include specific actionable recommendations: decentralisation through community- and school-based platforms, outreach programmes, peer-led approaches, and confidentiality-enhancing service design.**

**Revised text:** Lines 477–485.

## Language and Grammar (Reviewer #4)

| **Reviewer Concern** | **Action Taken** |
| --- | --- |
| Abstract too general (Reviewer #1) | Added quantitative indicators (24%, 15%) and specific barriers |
| Objective overloaded (Reviewer #1) | Refined to single primary aim |
| Methods incomplete (Reviewer #1) | Added setting, recruitment, ethics |
| Results list themes descriptively (Reviewers  #1, #2, #3) | Collapsed 5 themes into 3; added analytical pathway |
| **Summary table of themes and sub-themes**  **(Reviewers #1 and #2)** | **Added Table 3 (Lines 214–224)** |
| Theme 3 (Preferences) not clearly articulated | Now highlighted in bold with specific preferences listed |
| Sampling strategy unclear; "convenience sampling" incorrect (Reviewers #2, #3) | Corrected to purposive sampling; added detailed recruitment |
| Saturation rationale missing (Reviewers #1,  #2, #3) | Added explicit saturation statement |
| Participant characteristics table missing  (Reviewers #1, #2) | Added Tables 1 and 2 |
| Quotes lack attribution (Reviewers #1, #2) | Standardized quote format |
| Stakeholder perspectives merged (Reviewer  #3) | Differentiated perspectives clearly |
| Interpretive depth missing (Reviewers #1, #3) | Added interpretive commentary throughout |
| Trustworthiness criteria incomplete (Reviewer  #4) | Added credibility, dependability, confirmability, transferability |
| Discussion too narrow (Reviewers #2, #3, #4) | Expanded to include Ethiopia, Nigeria, Ghana, Kenya,  Tanzania |
| Language errors (Reviewer #4) | Corrected Line 152, Line 283, and throughout |
| References inconsistent (Reviewers #2, #3, #4) | Standardized formatting; added recent SSA literature |
| Data availability restrictions (All reviewers) | Deposited de-identified data in public repository; updated Data Availability Statement |

**Comment 17 (Reviewer #4):** Minor grammatical errors need to be reviewed. Specific line edits:

- Line 121: rephrase to "... shaping adolescent health-seeking behaviour."
- Line 229: replace "like" with "such as" (and amend where applicable throughout the manuscript).

**Response:** These corrections have been made. Line 121 now reads **"... shaping adolescent health-seeking behaviour."** Line 229 and other instances of "like" have been changed to **"such as"** where appropriate. The entire manuscript has been carefully proofread for additional grammatical and typographical errors.

**Revised text:** Lines 121, 229, and throughout.

## References

**Comment 18 (Reviewers #2, #3, and #4):** Ensure consistency of the referencing method. Include more recent literature and studies from other SSA countries.

**Response:** The reference list has been updated to ensure consistency in formatting (all now follow PLOS ONE style). **New references from Ethiopia, Nigeria, Ghana, Kenya, and Tanzania have been added (References 4, 5, 6, 23, 24, 26). Older references have been retained where they represent foundational or landmark studies. All references are now within the last 10 years except for theoretical foundations (Health Belief Model, Andersen's Behavioural Model).**

**Revised text:** References section (Lines 505–588).

# Summary of Key Changes in Response to Reviewers

# Additional Revisions Undertaken to Improve Quality

In addition to addressing all reviewer comments, we have made the following improvements:

- **COREQ alignment:** The manuscript has been reviewed against the COREQ checklist for qualitative studies, and all relevant items have been addressed (e.g., researcher characteristics, relationship with participants, theoretical framework).
- **Theory integration:** The Health Belief Model and Andersen's Behavioural Model are now more explicitly integrated into the analysis and discussion.
- **Language and readability:** The manuscript has been carefully proofread for clarity, grammar, and consistency.
- **Correction of sampling terminology:** All references to "convenience sampling" have been removed and replaced with accurate descriptions of **purposive sampling** throughout the manuscript and response letter.
- **Trustworthiness criteria:** Credibility, dependability, confirmability, and transferability are now explicitly addressed in the Methods section with citations (Lincoln & Guba, 1985; Nowell et al., 2017).

# Final Statement

We believe these revisions have substantially improved the manuscript's methodological rigor, analytical depth, transparency, and alignment with PLOS ONE's qualitative reporting standards. In particular, the collapse of five themes into three analytically coherent themes (Theme 1: Motivation, Theme 2: Barriers, Theme 3: Preferences), the addition of Table 3 (summary of themes and sub-themes), the correction of the sampling description to purposive sampling, and the explicit inclusion of trustworthiness criteria have significantly strengthened the manuscript.

We are deeply grateful for the reviewers' insightful feedback, which has helped us produce a stronger and more impactful manuscript. We have also addressed the data availability concerns by depositing a fully de-identified dataset in a public repository and updating the Data Availability Statement accordingly.

We hope the revised manuscript is now acceptable for publication in PLOS ONE.

Thank you for your consideration.

Sincerely,

**Noah Nyende (Corresponding Author)** on behalf of all co-authors
