# Supplementary material for: Stakeholder perspectives on adolescent sexual and reproductive health service utilization in Uganda’s Busoga region: A qualitative study
Source: PLoS One. 2026 Oct 1;21(10):e0358996. doi: 10.1371/journal.pone.0358996 (PMC13630231; doi:10.1371/journal.pone.0358996)
Supplement: Supplementary file 5 [file pone.0358996.s006.docx]

**Response to Reviewers**

**Manuscript ID:** PONE-D-26-00996
**Title:** *Stakeholder perspectives on adolescent sexual and reproductive health service utilization in Uganda's Busoga region: A qualitative study*
**Journal:** PLOS ONE

**Dear Editor and Reviewers,**

We sincerely thank you for your thorough, rigorous, and constructive review of our manuscript. Your detailed feedback has been invaluable in significantly strengthening the clarity, methodological transparency, analytical depth, and overall quality of our work. We have carefully revised the manuscript in response to all comments. Below, we provide a detailed, point-by-point response, with corresponding line references to the revised manuscript. **All major revisions and additions are highlighted in bold text** for ease of review.

**1. PLOS ONE Formatting and Style**

**Comment:** Ensure the manuscript complies with PLOS ONE formatting guidelines.

**Response:** The manuscript has been fully revised to comply with PLOS ONE formatting requirements, including structure, headings, referencing style, and file naming conventions. **The abstract now follows the structured format (Background, Methods, Results, Conclusion) as required.**

**2–4. Data Availability and Open Data Policy**

**Comment:** Clarify data sharing, restrictions, and repository deposition. (Reviewers #2, #3, and #4 raised concerns about data availability.)

**Response:** We have substantially revised the data availability approach to align fully with PLOS ONE's open data policy:

- **A fully de-identified qualitative dataset (focus group discussions and key informant interviews) has been prepared following established anonymization standards (e.g., BMJ 2010).**
- **All direct identifiers (names of participants, specific village names, health facilities, dates, and institutional affiliations) have been removed or generalized to protect participant confidentiality.**
- **The dataset has been deposited in a public repository .**
- **The Data Availability Statement has been updated to include the repository link and accession number.**
- **We confirm that written informed consent (and assent for minors) explicitly included permission for the use of anonymized data in research outputs and public repositories.**
- **No potentially identifiable information remains in the deposited dataset.**

This ensures full compliance with PLOS ONE's requirement that data underlying findings be publicly accessible while preserving participant confidentiality.

**Revised text:** Data Availability Statement (Lines 487–490); Ethical Considerations (Lines 188–196).

**Point-by-Point Responses to Reviewer Comments**

We have consolidated comments from **all four reviewers** and respond to each below.

**Abstract**

**Comment 1 (Reviewers #1 and #2):** The abstract background is too general. It needs greater specificity, quantitative indicators, and a clearer rationale for the Busoga region and stakeholder approach.

**Response:** The abstract background has been completely revised to include:

- **Specific quantitative indicators: 24% adolescent childbearing rate, 15% modern contraceptive prevalence among adolescents (Uganda Demographic and Health Survey 2022).**
- **Named barriers: sociocultural norms, poverty, limited health system capacity.**
- **Clear justification: the Busoga region is disproportionately affected by these challenges, yet no study has triangulated perspectives from adolescents, providers, and community/religious leaders.**

**Revised text:** Abstract, Lines 13–20.

**Comment 2 (Reviewer #1):** Replace "suitable services" with a more precise term.

**Response:** "Suitable services" has been replaced with **"adolescent-friendly SRH services"** throughout the abstract and manuscript.

**Revised text:** Abstract, Line 17; also consistent throughout.

**Comment 3 (Reviewers #1 and #2):** The objective combines several aims, diluting clarity. It should more directly reflect the primary research purpose.

**Response:** The objective has been refined to focus on the **single primary aim: to explore stakeholder perspectives to provide an integrated understanding of factors influencing adolescent SRH service utilization.** The secondary elements (barriers, facilitators, strategies) now appear in the results and conclusion.

**Revised text:** Abstract, Lines 19–20.

**Comment 4 (Reviewer #1):** The abstract methods section is incomplete.

**Response:** The methods section has been expanded to include:

- **Study setting (Iganga and Bugweri districts, Busoga region).**
- **Recruitment approach: adolescents through peer networks; key informants purposively selected.**
- **A clear statement of ethical approval and consent procedures.**

**Revised text:** Abstract, Lines 22–28.

**Comment 5 (Reviewers #1, #2, and #3):** The results section lists themes descriptively but does not articulate the main analytical insight. The abstract and results should reflect the collapse of five themes into three.

**Response:** Thank you for this critical observation. We have substantially restructured the results to address this concern.

**First, the original five themes have been collapsed into three analytically coherent themes** that follow a clear logical progression from **motivation → barriers → solutions**:

| **Original Five Themes** | **Collapsed into Three Themes** |
| --- | --- |
| 1. Positive orientation toward SRH prevention amid sexual self-discovery | **Theme 1: Positive orientation toward SRH prevention amid sexual self-discovery** (motivation) |
| 2. Persistent misconceptions and fears surrounding contraception | ↓ |
| 3. Access barriers related to distance, privacy, and provider attitudes | **Theme 2: Barriers to SRH service utilization** (barriers) |
| 5. Sociocultural and system-level constraints | ↓ |
| 4. Strong preferences for free, confidential, peer-informed, and community-based SRH services | **Theme 3: Preferences for adolescent-friendly and community-based services** (solutions) |

**Second, the results section has been reframed to highlight the core analytical insight: adolescents are motivated to use SRH services, but this motivation is constrained by intersecting barriers, leading them to prefer alternative service models.** The three themes are now presented within this analytical narrative rather than as a simple list.

**Third, Theme 3 (Preferences) is now clearly articulated and highlighted in bold throughout the results section.** This theme captures adolescents' strong preferences for:

- **Free, confidential, and accessible services**
- **Community- and school-based delivery platforms**
- **Peer-led approaches (because "we believe our peers")**
- **Outreach programmes and integration with existing community structures (e.g., village health teams, religious gatherings)**

**Revised text:** Abstract, Lines 30–36; Results, Lines 216–224 (theme overview) and Lines 359–381 (**Theme 3: Preferences for adolescent-friendly and community-based services**).

**Methods (Data Collection, Recruitment, and Transparency)**

**Comment 6 (Reviewers #1, #2, and #3):** Expand the data collection/procedure section to include recruitment and sampling processes, saturation rationale, and data security.

**Response:** The Methods section has been substantially expanded to include:

- **Detailed recruitment processes for adolescents (via community mobilisers and youth peer leaders) and key informants (purposively selected based on institutional roles).**
- **Explicit saturation rationale: data collection continued until no new patterns emerged, achieved after 4 FGDs and 12 KIIs, confirmed through team debriefing and concurrent transcript review.**
- **Data security procedures: anonymization, password-protected devices, restricted access to the research team.**

**Revised text:** Lines 99–162.

**Comment 7 (Reviewers #1, #2, and #3):** Explain how adolescents and key informants were identified and approached. Add inclusion/exclusion criteria, definitions of urban/rural residence, and whether any participants declined. **Reviewer #2 specifically noted that the sampling strategy needed clarification, and Reviewer #3 noted that convenience sampling was incorrectly stated.**

**Response:** This section has been significantly expanded. **We have corrected the sampling description: it is purposive sampling, not convenience sampling.**

- **Adolescents were purposively sampled to capture diverse perspectives across gender, age, and residential contexts. Recruitment was facilitated through community mobilisers and youth peer leaders, with outreach in communities.**
- **Inclusion criteria for adolescents: age 13–19 years, residence in the study area for at least six months, and willingness to participate. No exclusion criteria were applied beyond these.**
- **Key informants were purposively selected based on formal institutional roles (health providers: nurses and clinical officers; religious leaders: clergy, pastors, imams; cultural leaders: clan leaders and elders) and recognized community authority.**
- **A small number of adolescents declined participation due to time constraints or discomfort discussing SRH in group settings. No refusals were recorded among key informants.**

**Revised text:** Lines 110–146.

**Comment 8 (Reviewers #1 and #2):** Provide more operational detail for FGD procedures: group size, composition rules, presence of note-takers, language, and handling of sensitive topics.

**Response:** The following details have been added:

- **Each FGD comprised 9–10 participants.**
- **Groups were stratified by sex and residence. Age stratification was implicit: female groups were younger (13–17 years); male groups were older (17–19 years) to reflect developmental stages and facilitate open discussion.**
- **Each session was facilitated by a trained moderator of the same sex as participants. A note-taker was present; no external observers were in the room.**
- **All FGDs were conducted in Lusoga, the local language.**
- **Sensitive topics were managed using neutral phrasing, probing techniques, and reassurance of confidentiality. Participants were reminded that they could skip any question or withdraw at any time.**

**Revised text:** Lines 132–138.

**Comment 9 (Reviewers #1, #2, and #3):** Clarify how the team determined that sufficient saturation was reached for FGDs and KIIs.

**Response:** A clear saturation statement has been added:

- **Sampling proceeded iteratively, with regular team debriefings and concurrent review of transcripts.**
- **Saturation was considered reached when no new themes emerged, categories were well-developed, and additional interviews were unlikely to yield meaningful new insights.**
- **This occurred after 4 FGDs and 12 KIIs, at which point data collection ceased.**

**Revised text:** Lines 122–124 (sampling) and Lines 151–155 (analysis).

**Comment 10 (Reviewer #4):** Ensure trustworthiness parameters (credibility, dependability, confirmability, transferability) are explicitly stated within the context of the research. Cite relevant literature.

**Response:** Thank you for this important guidance. The Methods section has been revised to explicitly address all four trustworthiness criteria:

- **Credibility: achieved through prolonged engagement during discussions, probing techniques, triangulation of multiple stakeholder perspectives, and back-translation of selected transcripts (Lines 132–138, 151–155).**
- **Dependability: ensured through regular team debriefings, independent coding by two researchers, and maintenance of a comprehensive audit trail (Lines 151–162).**
- **Confirmability: supported by reflexive journaling, documentation of assumptions and positionality, and grounding interpretations in participant accounts rather than researcher predispositions (Lines 162–168).**
- **Transferability: acknowledged that findings are primarily applicable to the Busoga region (Lines 470–471).**

**Revised text:** Lines 132–138, 151–168, 470–471.

**Results Section**

**Comment 11a (Reviewers #1 and #2):** No detailed description of participants is provided. A table summarizing participant characteristics is essential.

**Response:** **Two new tables have been added:**

- **Table 1: Composition of Focus Group Discussions (FGDs) by site, gender, mean age, schooling status, and number of participants.**
- **Table 2: Composition of Key Informant Interviews (KIIs) by participant group, gender, district, rural/urban location, and total participants.**

**Revised text:** Lines 201–212 (Tables 1 and 2).

**Comment 11b (Reviewers #1 and #2 – Summary table of themes and sub-themes):** Reviewer #1 specifically noted: *"The authors may consider including a summary table of themes and subthemes to improve readability."* Reviewer #2 similarly suggested organizing the findings with a table.

**Response:** Thank you for this practical suggestion. **We have included Table 3 in the revised manuscript**, which provides a clear, at-a-glance summary of the three main themes and their corresponding sub-themes.

**This table directly addresses Reviewer #1's request for improved readability and Reviewer #2's suggestion for better organization of findings.** It appears immediately after the participant characteristics tables (Tables 1 and 2) and before the detailed thematic narrative, allowing readers to orient themselves before reading the full results.

**Revised text:** Lines 214–224 (Table 3).

**Comment 11c (Reviewers #1, #2, and #3):** Themes are presented descriptively; the analysis should explain how themes relate to each other. The collapse of five themes into three should be clearly shown.

**Response:** The results section has been restructured to present **three interconnected themes** that follow a clear analytical progression:

1. **Theme 1: Positive orientation toward SRH prevention amid sexual self-discovery** (motivation and agency).
2. **Theme 2: Barriers to SRH service utilization** (sub-themes: misconceptions, structural/interpersonal barriers, sociocultural/system constraints).
3. **Theme 3: Preferences for adolescent-friendly and community-based services** (solutions).

**An explicit analytical pathway has been added: adolescents show motivation (Theme 1) → but face barriers (Theme 2) → leading them to prefer alternative service models (Theme 3).**

**Within Theme 3, we now clearly articulate adolescents' specific preferences, including: free and confidential services; community- and school-based delivery platforms; peer-led approaches ("because we believe our peers"); outreach programmes; and integration with existing community structures such as village health teams and religious gatherings.**

**Revised text:** Lines 216–224 (overview), and throughout the Results section (Lines 233–381).

**Comment 11d (Reviewers #1 and #2):** Quotes lack sufficient detail. Use a standardized format.

**Response:** All quotes have been standardized to the following format: ***(Quote text, Participant group, FGD/KII number, district, rural/urban).*** For example: *(Female adolescent, FGD 2, rural Iganga).* This has been applied consistently throughout the Results section.

**Revised text:** Throughout Lines 233–381.

**Comment 11e (Reviewer #3):** The analysis sometimes merges perspectives without clearly distinguishing them. Provide clearer differentiation between stakeholder groups.

**Response:** The analysis has been revised to clearly distinguish perspectives:

- **Adolescents: emphasize privacy, stigma, fear of judgment, and preferences for peer-led services.**
- **Health providers: highlight service capacity issues, stockouts, staffing shortages, and efforts to reach adolescents through outreach.**
- **Religious and cultural leaders: stress moral norms, early marriage practices, religious prohibitions on contraception, and the role of community guidance.**

**This differentiation is now explicit throughout the Results section.**

**Revised text:** Lines 233–381 (e.g., separate quotes and analysis for each stakeholder group within each theme).

**Comment 11f (Reviewers #1 and #3):** Results are largely descriptive; interpretive insight is needed. Add reflexivity and analytical interpretation.

**Response:** Interpretive commentary has been added throughout the Results section, including:

- **Parental silence: how limited parent–adolescent communication drives self-directed learning and reliance on peer networks.**
- **Misinformation: how fears of infertility and cancer, reinforced by community and religious narratives, outweigh perceived benefits of contraception.**
- **Barrier interaction: how distance, privacy, provider attitudes, and stockouts combine to create compounded access failures.**
- **Preference logic: why adolescents prefer community-based and peer-led services as a direct response to stigma and confidentiality concerns (explicitly developed in Theme 3).**

**Revised text:** Lines 233–381 (interpretive statements added within each theme).

**Comment 11g (Reviewers #1 and #2):** The results section should briefly indicate whether saturation was achieved.

**Response:** A saturation statement has been added to the Results section:

**Revised text:** Lines 122-124, 151–155.

**Comment 11h (Reviewer #3):** The integration of systems and cultural factors (formerly Theme 5) reads as fragmented. Better highlight interactions.

**Response:** Theme 2 (which now incorporates the former Theme 5) has been substantially revised to show the **interaction between religion, early marriage norms, poverty, and health system weaknesses.** The sub-theme now explicitly demonstrates how:

- **Religious norms and early marriage practices constrain adolescents' agency.**
- **Health system stockouts and staffing shortages compound these cultural barriers.**
- **The combination of normative expectations and service gaps leads to delayed or foregone care.**

**Revised text:** Lines 277–358 (previously Theme 5, now integrated into Theme 2).

**Discussion**

**Comment 12 (Reviewers #2, #3, and #4):** The discussion would benefit from broader comparison with studies from other sub-Saharan African countries and similar settings outside Africa. Reviewer #4 specifically noted that only one study from South Africa was cited.

**Response:** The discussion has been substantially expanded to include comparisons with studies from **Ethiopia, Nigeria, Ghana, Kenya, Tanzania, and other sub-Saharan African countries.** New references have been added (References 4, 5, 6, 23, 24, 26). The discussion now explicitly situates our findings within the broader SSA evidence base while highlighting the unique contribution of multi-stakeholder triangulation in the Busoga context.

**Revised text:** Lines 400–445.

**Comment 13 (Reviewer #2):** Some sections explaining the theoretical models are relatively long and could be more directly connected to the study findings.

**Response:** The theoretical application sections have been shortened and more tightly integrated with specific findings from each theme. Direct links between HBM constructs (perceived barriers, perceived benefits) and Andersen's model (predisposing, enabling, need factors) are now explicitly mapped to participant quotes.

**Revised text:** Lines 420–445.

**Implications for Policy and Practice**

**Comment 14 (Reviewer #2):** Provide more concrete recommendations for health systems, such as training of providers, strengthening youth-friendly services, or community engagement strategies.

**Response:** The Implications section has been expanded to include **specific, actionable recommendations:**

- **Health system level: targeted provider training in non-judgmental communication, dedicated adolescent service corners, flexible service hours, and privacy-enhancing infrastructure.**
- **Community level: structured engagement with parents, religious leaders, and cultural gatekeepers through dialogue forums and faith-based sensitization initiatives.**
- **School level: peer education programmes and integration of SRH information into existing curricula.**
- **Supply chain: strengthening commodity supply chain management to reduce stockouts, particularly for contraceptives in rural facilities.**

**Revised text:** Lines 451–467.

**Strengths and Limitations**

**Comment 15 (Reviewer #2):** The limitations section could also mention possible selection bias due to sampling and limitations in generalizability beyond the study region.

**Response:** The limitations section has been expanded to explicitly acknowledge:

- **Geographic scope** – limited to two districts in Busoga, limiting transferability
- **Context specificity** – findings primarily grounded in Busoga region
- **Social desirability bias** – participants may have given socially acceptable responses
- **Translation challenges** – potential loss of nuance in translation from Lusoga to English

**Revised text:** Lines 470–475.

**Conclusion**

**Comment 16 (Reviewers #1 and #2):** The conclusion overgeneralizes findings to "similar low-resource settings" without sufficient caution. Emphasize specific actionable recommendations.

**Response:** The conclusion has been revised to:

- **Emphasize that the findings are primarily applicable to the Busoga region of eastern Uganda.**
- **Note cautiously that the insights may be relevant to other contexts with comparable sociocultural norms, economic challenges, and health system constraints, but that direct replication requires contextual adaptation.**
- **Include specific actionable recommendations: decentralisation through community- and school-based platforms, outreach programmes, peer-led approaches, and confidentiality-enhancing service design.**

**Revised text:** Lines 477–485.

**Language and Grammar (Reviewer #4)**

| **Reviewer Concern** | **Action Taken** |
| --- | --- |
| Abstract too general (Reviewer #1) | Added quantitative indicators (24%, 15%) and specific barriers |
| Objective overloaded (Reviewer #1) | Refined to single primary aim |
| Methods incomplete (Reviewer #1) | Added setting, recruitment, ethics |
| Results list themes descriptively (Reviewers #1, #2, #3) | Collapsed 5 themes into 3; added analytical pathway |
| **Summary table of themes and sub-themes (Reviewers #1 and #2)** | **Added Table 3 (Lines 214–224)** |
| Theme 3 (Preferences) not clearly articulated | Now highlighted in bold with specific preferences listed |
| Sampling strategy unclear; "convenience sampling" incorrect (Reviewers #2, #3) | Corrected to purposive sampling; added detailed recruitment |
| Saturation rationale missing (Reviewers #1, #2, #3) | Added explicit saturation statement |
| Participant characteristics table missing (Reviewers #1, #2) | Added Tables 1 and 2 |
| Quotes lack attribution (Reviewers #1, #2) | Standardized quote format |
| Stakeholder perspectives merged (Reviewer #3) | Differentiated perspectives clearly |
| Interpretive depth missing (Reviewers #1, #3) | Added interpretive commentary throughout |
| Trustworthiness criteria incomplete (Reviewer #4) | Added credibility, dependability, confirmability, transferability |
| Discussion too narrow (Reviewers #2, #3, #4) | Expanded to include Ethiopia, Nigeria, Ghana, Kenya, Tanzania |
| Language errors (Reviewer #4) | Corrected Line 152, Line 283, and throughout |
| References inconsistent (Reviewers #2, #3, #4) | Standardized formatting; added recent SSA literature |
| Data availability restrictions (All reviewers) | Deposited de-identified data in public repository; updated Data Availability Statement |

**Comment 17 (Reviewer #4):** Minor grammatical errors need to be reviewed. Specific line edits:

- Line 121: rephrase to "... shaping adolescent health-seeking behaviour."
- Line 229: replace "like" with "such as" (and amend where applicable throughout the manuscript).

**Response:** These corrections have been made. Line 121 now reads **"... shaping adolescent health-seeking behaviour."** Line 229 and other instances of "like" have been changed to **"such as"** where appropriate. The entire manuscript has been carefully proofread for additional grammatical and typographical errors.

**Revised text:** Lines 121, 229, and throughout.

**References**

**Comment 18 (Reviewers #2, #3, and #4):** Ensure consistency of the referencing method. Include more recent literature and studies from other SSA countries.

**Response:** The reference list has been updated to ensure consistency in formatting (all now follow PLOS ONE style). **New references from Ethiopia, Nigeria, Ghana, Kenya, and Tanzania have been added (References 4, 5, 6, 23, 24, 26). Older references have been retained where they represent foundational or landmark studies. All references are now within the last 10 years except for theoretical foundations (Health Belief Model, Andersen's Behavioural Model).**

**Revised text:** References section (Lines 505–588).

**Summary of Key Changes in Response to Reviewers**

**Additional Revisions Undertaken to Improve Quality**

In addition to addressing all reviewer comments, we have made the following improvements:

- **COREQ alignment:** The manuscript has been reviewed against the COREQ checklist for qualitative studies, and all relevant items have been addressed (e.g., researcher characteristics, relationship with participants, theoretical framework).
- **Theory integration:** The Health Belief Model and Andersen's Behavioural Model are now more explicitly integrated into the analysis and discussion.
- **Language and readability:** The manuscript has been carefully proofread for clarity, grammar, and consistency.
- **Correction of sampling terminology:** All references to "convenience sampling" have been removed and replaced with accurate descriptions of **purposive sampling** throughout the manuscript and response letter.
- **Trustworthiness criteria:** Credibility, dependability, confirmability, and transferability are now explicitly addressed in the Methods section with citations (Lincoln & Guba, 1985; Nowell et al., 2017).

**Final Statement**

We believe these revisions have substantially improved the manuscript's methodological rigor, analytical depth, transparency, and alignment with PLOS ONE's qualitative reporting standards. In particular, the collapse of five themes into three analytically coherent themes (Theme 1: Motivation, Theme 2: Barriers, Theme 3: Preferences), the addition of Table 3 (summary of themes and sub-themes), the correction of the sampling description to purposive sampling, and the explicit inclusion of trustworthiness criteria have significantly strengthened the manuscript.

We are deeply grateful for the reviewers' insightful feedback, which has helped us produce a stronger and more impactful manuscript. We have also addressed the data availability concerns by depositing a fully de-identified dataset in a public repository and updating the Data Availability Statement accordingly.

We hope the revised manuscript is now acceptable for publication in PLOS ONE.

Thank you for your consideration.

Sincerely,
**Noah Nyende (Corresponding Author)**
on behalf of all co-authors
