# Supplementary material for: Stakeholder perspectives on adolescent sexual and reproductive health service utilization in Uganda’s Busoga region: A qualitative study
Source: PLoS One. 2026 Oct 1;21(10):e0358996. doi: 10.1371/journal.pone.0358996 (PMC13630231; doi:10.1371/journal.pone.0358996)
Supplement: Supplementary file 6 [file pone.0358996.s007.docx]

**Response to Reviewers**

Manuscript PONE-D-26-00996R1

*“Stakeholder perspectives on adolescent sexual and reproductive health service utilization in Uganda’s Busoga region: A qualitative study”*

Dear Dr. Matovu,

Thank you for the opportunity to revise our manuscript and for the constructive feedback from you and the four reviewers. We appreciate the reviewers’ positive assessment of the revised manuscript and have carefully addressed the remaining editorial points raised. Below we provide a point-by-point response to each comment. Reviewer and editor comments are shown in italics, and our responses follow.

**Academic Editor – Journal Requirements**

**Comment 1**

*If the reviewer comments include a recommendation to cite specific previously published works, please review and evaluate these publications to determine whether they are relevant and should be cited.*

**Response:** None of the reviewers recommended specific additional works to cite in this round of review. We have therefore not added any new citations in response to this requirement.

**Comment 2**

*Please review your reference list to ensure that it is complete and correct. If you have cited papers that have been retracted, please include the rationale for doing so in the manuscript text, or remove these references and replace them with relevant current references.*

**Response:** We have reviewed all 28 references in the manuscript and confirm that none has been retracted.

**Reviewer #1**

*In my opinion, the major concerns raised during the review process have been adequately addressed, and the manuscript now makes a meaningful contribution to the understanding of adolescent sexual and reproductive health service utilization in low-resource settings. I therefore recommend acceptance of the manuscript for publication in PLOS ONE.*

**Response:** We thank the reviewer for this positive assessment and for their time and constructive input across both rounds of review.

**Reviewer #2**

*I have only a few minor editorial observations. The authors should ensure consistency in reporting participant numbers, terminology (e.g., utilization/utilisation), and formatting throughout the manuscript and carefully proofread the final version to eliminate any remaining typographical or formatting inconsistencies.*

**Response:** We thank the reviewer for this careful reading and have addressed each of the three points raised:

**• Participant numbers:** The number of key informant interviews was previously written inconsistently as “twelve” in the Methods (Participants, Data collection, and Data analysis subsections) while appearing as the numeral “12” in the Results section and Table 2. We have standardized this to the numeral “12” throughout the manuscript, consistent with reporting numbers of 10 or greater as numerals.

**• Terminology:** The term “utilisation” (British spelling) appeared once in the Results section while “utilization” (American spelling) was used throughout the rest of the manuscript. We have corrected this instance so that “utilization” is used consistently throughout.

**• Formatting/proofreading:** We identified that British and American spelling conventions were mixed throughout the manuscript (e.g., “characterised/characterized,” “organisation/organization,” “recognised/recognized,” “minimise/minimize,” “familiarisation/familiarization,” “mobilisation/mobilization”). We have standardized spelling to American English throughout, consistent with the predominant convention used in the original submission. We also standardized abbreviation use in the Discussion (e.g., consistent use of “SRH” and “HBM” after first definition) and re-proofread the full manuscript for residual typographical inconsistencies.

**Reviewer #3**

*The manuscript would benefit from a professional editor/proof reading. There are minor issues that need attention.*

**Response:** We thank the reviewer for this feedback. We have carefully proofread the entire manuscript and corrected the spelling and formatting inconsistencies described above (standardizing British/American spelling conventions, correcting numeral usage for participant counts, and correcting reference formatting). We have also reviewed the manuscript line by line for residual typographical errors.

**Reviewer #4**

*Thank you and well done for sufficiently addressing the review comments. All the best!*

**Response:** We are grateful for the reviewer’s time and encouraging feedback throughout the review process.

**Summary of changes**

In summary, the following changes were made in this revision:

1. Standardized the reported number of key informant interviews to the numeral “12” throughout the Methods and Results (previously “twelve” in Methods).

2. Corrected a single instance of “utilisation” to “utilization” in the Results for consistency with the rest of the manuscript.

3. Standardized British/American spelling conventions to American English throughout the manuscript.

4. Standardized the reporting of residence duration in the inclusion criteria (“six months” to “6 months”) for consistency with numeral use for measurements.

5. Confirmed that no cited references have been retracted.

We thank the Academic Editor and all four reviewers again for their time and valuable feedback, which we believe has strengthened the manuscript.

Sincerely,

Dr. Nyende and co-authors
