# Supplementary material for: Stakeholder perspectives on adolescent sexual and reproductive health service utilization in Uganda’s Busoga region: A qualitative study
Source: PLoS One. 2026 Oct 1;21(10):e0358996. doi: 10.1371/journal.pone.0358996 (PMC13630231; doi:10.1371/journal.pone.0358996)
Supplement: Peer review history

# Peer review: Decision Letter 0

**DOI:** 10.1371/journal.pone.0358996.r001

**Authors:** Joseph Matovu

26 Mar 2026

Dear Dr. Nyende,

Thank you for submitting your manuscript to PLOS ONE. After careful consideration, we feel that it has merit but does not fully meet PLOS ONE’s publication criteria as it currently stands. Therefore, we invite you to submit a revised version of the manuscript that addresses the points raised during the review process.

Please submit your revised manuscript by May 10 2026 11:59PM. If you will need more time than this to complete your revisions, please reply to this message or contact the journal office at plosone@plos.org. When you're ready to submit your revision, log on to https://www.editorialmanager.com/pone/ and select the 'Submissions Needing Revision' folder to locate your manuscript file.

- A letter that responds to each point raised by the academic editor and reviewer(s). You should upload this letter as a separate file labeled 'Response to Reviewers'.
- A marked-up copy of your manuscript that highlights changes made to the original version. You should upload this as a separate file labeled 'Revised Manuscript with Track Changes'.
- An unmarked version of your revised paper without tracked changes. You should upload this as a separate file labeled 'Manuscript'.

If you would like to make changes to your financial disclosure, please include your updated statement in your cover letter. Guidelines for resubmitting your figure files are available below the reviewer comments at the end of this letter.

If applicable, we recommend that you deposit your laboratory protocols in protocols.io to enhance the reproducibility of your results. Protocols.io assigns your protocol its own identifier (DOI) so that it can be cited independently in the future. For instructions see: https://journals.plos.org/plosone/s/submission-guidelines#loc-laboratory-protocols. Additionally, PLOS ONE offers an option for publishing peer-reviewed Lab Protocol articles, which describe protocols hosted on protocols.io. Read more information on sharing protocols at https://plos.org/protocols?utm_medium=editorial-email&utm_source=authorletters&utm_campaign=protocols.

As the corresponding author, your ORCID iD is verified in the submission system and will appear in the published article. PLOS supports the use of ORCID, and we encourage all coauthors to register for an ORCID iD and use it as well. Please encourage your coauthors to verify their ORCID iD within the submission system before final acceptance, as unverified ORCID iDs will not appear in the published article. *Only*  the individual author can complete the verification step; PLOS staff *cannot*  verify ORCID iDs on behalf of authors.

We look forward to receiving your revised manuscript.

Kind regards,

Joseph KB Matovu, Ph.D.

Academic Editor

PLOS One

Journal Requirements:

When submitting your revision, we need you to address these additional requirements.

1. Please ensure that your manuscript meets PLOS ONE's style requirements, including those for file naming. The PLOS ONE style templates can be found at

https://journals.plos.org/plosone/s/file?id=wjVg/PLOSOne_formatting_sample_main_body.pdf and https://journals.plos.org/plosone/s/file?id=ba62/PLOSOne_formatting_sample_title_authors_affiliations.pdf

2. We note that you have indicated that there are restrictions to data sharing for this study. For studies involving human research participant data or other sensitive data, we encourage authors to share de-identified or anonymized data. However, when data cannot be publicly shared for ethical reasons, we allow authors to make their data sets available upon request. For information on unacceptable data access restrictions, please see http://journals.plos.org/plosone/s/data-availability#loc-unacceptable-data-access-restrictions.

Before we proceed with your manuscript, please address the following prompts:

a) If there are ethical or legal restrictions on sharing a de-identified data set, please explain them in detail (e.g., data contain potentially identifying or sensitive patient information, data are owned by a third-party organization, etc.) and who has imposed them (e.g., a Research Ethics Committee or Institutional Review Board, etc.). Please also provide contact information for a data access committee, ethics committee, or other institutional body to which data requests may be sent.

b) If there are no restrictions, please upload the minimal anonymized data set necessary to replicate your study findings to a stable, public repository and provide us with the relevant URLs, DOIs, or accession numbers. Please see http://www.bmj.com/content/340/bmj.c181.long for guidelines on how to de-identify and prepare clinical data for publication. For a list of recommended repositories, please see https://journals.plos.org/plosone/s/recommended-repositories. You also have the option of uploading the data as Supporting Information files, but we would recommend depositing data directly to a data repository if possible.

Please update your Data Availability statement in the submission form accordingly.

3. In the online submission form, you indicated that [De-identified qualitative transcripts are available from the corresponding author upon reasonable request and subject to approval by the relevant ethics committees.].

All PLOS journals now require all data underlying the findings described in their manuscript to be freely available to other researchers, either 1. In a public repository, 2. Within the manuscript itself, or 3. Uploaded as supplementary information.

This policy applies to all data except where public deposition would breach compliance with the protocol approved by your research ethics board. If your data cannot be made publicly available for ethical or legal reasons (e.g., public availability would compromise patient privacy), please explain your reasons on resubmission and your exemption request will be escalated for approval.

4. When completing the data availability statement of the submission form, you indicated that you will make your data available on acceptance. We strongly recommend all authors decide on a data sharing plan before acceptance, as the process can be lengthy and hold up publication timelines. Please note that, though access restrictions are acceptable now, your entire data will need to be made freely accessible if your manuscript is accepted for publication. This policy applies to all data except where public deposition would breach compliance with the protocol approved by your research ethics board. If you are unable to adhere to our open data policy, please kindly revise your statement to explain your reasoning and we will seek the editor's input on an exemption. Please be assured that, once you have provided your new statement, the assessment of your exemption will not hold up the peer review process.

5. We note that this manuscript consists of interview transcripts. Can you please confirm that all participants gave consent for interview transcript to be published?

If they DID provide consent for these transcripts to be published, please also confirm that the transcripts do not contain any potentially identifying information (or let us know if the participants consented to having their personal details published and made publicly available). We consider the following details to be identifying information:

- Names, nicknames, and initials

- Age more specific than round numbers

- GPS coordinates, physical addresses, IP addresses, email addresses

- Information in small sample sizes (e.g. 40 students from X class in X year at X university)

- Specific dates (e.g. visit dates, interview dates)

- ID numbers

Or, if the participants DID NOT provide consent for these transcripts to be published:

- Provide a de-identified version of the data or excerpts of interview responses

- Provide information regarding how these transcripts can be accessed by researchers who meet the criteria for access to confidential data, including:

a) the grounds for restriction

b) the name of the ethics committee, Institutional Review Board, or third-party organization that is imposing sharing restrictions on the data

c) a non-author, institutional point of contact that is able to field data access queries, in the interest of maintaining long-term data accessibility.

d) Any relevant data set names, URLs, DOIs, etc. that an independent researcher would need in order to request your minimal data set.

For further information on sharing data that contains sensitive participant information, please see: https://journals.plos.org/plosone/s/data-availability#loc-human-research-participant-data-and-other-sensitive-data

If there are ethical, legal, or third-party restrictions upon your dataset, you must provide all of the following details (https://journals.plos.org/plosone/s/data-availability#loc-acceptable-data-access-restrictions):

1. A complete description of the dataset

2. The nature of the restrictions upon the data (ethical, legal, or owned by a third party) and the reasoning behind them

3. The full name of the body imposing the restrictions upon your dataset (ethics committee, institution, data access committee, etc.)

4. If the data are owned by a third party, confirmation of whether the authors received any special privileges in accessing the data that other researchers would not have

5. Direct, non-author contact information (preferably email) for the body imposing the restrictions upon the data, to which data access requests can be sent

If the reviewer comments include a recommendation to cite specific previously published works, please review and evaluate these publications to determine whether they are relevant and should be cited. There is no requirement to cite these works unless the editor has indicated otherwise.

[Note: HTML markup is below. Please do not edit.]

Reviewers' comments:

Reviewer's Responses to Questions

**Comments to the Author**

1. Is the manuscript technically sound, and do the data support the conclusions?

Reviewer #1: Yes

Reviewer #2: Yes

Reviewer #3: Partly

Reviewer #4: Yes

**********

2. Has the statistical analysis been performed appropriately and rigorously?

Reviewer #1: Yes

Reviewer #2: N/A

Reviewer #3: N/A

Reviewer #4: Yes

**********

3. Have the authors made all data underlying the findings in their manuscript fully available?

The PLOS Data policy

Reviewer #1: Yes

Reviewer #2: No

Reviewer #3: Yes

Reviewer #4: No

**********

4. Is the manuscript presented in an intelligible fashion and written in standard English?

Reviewer #1: Yes

Reviewer #2: Yes

Reviewer #3: Yes

Reviewer #4: Yes

**********

Reviewer #1: This manuscript explores stakeholder perspectives on adolescent sexual and reproductive health (ASRH) service utilisation in the Busoga region of Uganda using qualitative research methods. The topic is highly relevant given the persistent barriers adolescents face in accessing sexual and reproductive health services in sub-Saharan Africa. The study addresses an important public health issue and contributes to the understanding of sociocultural, health system, and community-level factors influencing ASRH service uptake. The manuscript is generally well structured; however, several methodological clarifications and improvements in reporting are necessary to enhance transparency, rigor, and reproducibility.

Reviewer #2: Review of manuscript PONE-D-26-00996

Manuscript title: Stakeholders' perspectives on adolescent sexual and reproductive health service utilization in Uganda’s Busoga region: A qualitative study

Summary

This manuscript presents a qualitative study exploring the barriers and facilitators to the utilization of adolescent sexual and reproductive health (SRH) services in the Busoga region of Uganda. The research is timely and relevant, addressing a critical public health issue in low-resource settings. The strength of the study lies in its multi-stakeholder perspective, triangulating the views of adolescents, health providers, and community/religious leaders. The methodology is sound, the analysis is rigorous, and the findings, while confirming known barriers, provide rich, context-specific insights valuable for designing interventions.

Detail comments;

1. The study presents the results of original research

Assessment; yes

Comment: The triangulation of multiple stakeholders' perspectives is a notable strength and adds originality to the study

2. Results reported have not been published elsewhere

Assessment: There is no indication in the manuscript that the results have been previously published. The study appears to report new empirical findings

Comment: The authors should confirm that the findings have not been published elsewhere and that the manuscript is not under consideration by another journal

3. Experiments, statistics, and other analyses are performed to a high technical standard and are described in sufficient detail

Assessment: overall qualitative methodology is appropriate for the study objectives. The manuscript describes the use of FDG and KII, thematic analysis, and qualitative data management software.

Comment:

However, some methodological details could be strengthened, particularly

• Clarifying the rationale for the sampling strategy used for adolescent participants

• Providing more explanation of how data saturation was determined

• Further describing the strategies used ensures the rigor and trustworthiness of qualitative analysis (e.g., credibility, dependability, reflexibility)

4. Conclusions are presented appropriately and are supported by the data

Assessment;

Yes, the conclusions are generally consistent with the findings presented in the results section. The identified themes regarding misconceptions about contraception, sociocultural barriers, provider attitudes, and structural constraints are well supported by participant quotations.

Comment: The conclusions appropriately indicate the need for adolescent-friendly services and community-based approaches to improve SRH service utilization

5. The article is presented in an intelligible fashion and is written in standard English.

Assessment:

The manuscript is generally well written and clearly structured. The language is understandable, and the argument flows logically from the introduction to the conclusion

Comment: Minor grammatical and typographical errors are present throughout the manuscript (e.g., spacing and punctuation issues). A careful proofreading or language edit would improve readability.

6. The research meets all applicable standards for the ethics of experimentation and research integrity.

Assessment:

Yes, the manuscript reports ethical approval from the relevant ethics committees and describes informed consent procedures for participants, including parental consent and adolescent assent for minors.

Assessment:

Yes. The manuscript reports ethical approval from the relevant ethics committees and describes informed consent procedures for participants, including parental consent and adolescent assent for minors.

7. The article adheres to appropriate reporting guidelines and community standards for data availability.

Assessment: partially satisfied

The manuscript includes a data availability statement indicating that de-identified transcripts may be available upon reasonable request due to the sensitive nature of the data

Comment:

However, the journal’s policy typically encourages deposition of underlying data in a publicly accessible repository when possible. The authors should clarify whether anonymized transcripts or coded excerpts could be shared in a repository, or provide a stronger justification for the current restriction

Specific Comments (section by section) and Suggestions for Revision

1. Title

The title clearly reflects the focus of the study and identifies the population, topic, and geographic context. It appropriately indicates that the study uses stakeholder perspectives and qualitative methods.

Suggestion:

The title is appropriate, but the authors may consider specifying “qualitative study” earlier in the title to make the methodological approach immediately clear.

2. Abstract

The abstract clearly summarizes the purpose, study design, and major findings.

3. Introduction

The introduction provides relevant background on adolescent sexual and reproductive health (SRH) challenges globally and in sub-Saharan Africa. It appropriately indicates the importance of improving SRH service utilisation among adolescents.

Strengths:

The problem statement is clear.

Relevant statistics on adolescent pregnancy and contraceptive use in Uganda are presented. The research gap is identified, particularly the limited triangulation of perspectives from adolescents, community leaders, and health providers.

Suggestions for improvement:

Some references cited in the introduction are relatively old. Including more recent literature (last 5–7 years) on adolescent SRH services in sub-Saharan Africa would strengthen the context.

The authors may clarify more explicitly how their study adds to existing literature, especially regarding the multi-stakeholder perspective.

The transition from the literature review to the study objective could be slightly improved to enhance the flow of the narrative.

4. Materials and Methods

The methods section generally provides a clear description of the qualitative design and analytical approach.

Areas that need clarification:

Sampling Strategy

The authors report using convenience sampling for adolescents and purposive sampling for key informants. However, the rationale for this approach is not fully explained. Additional details about participant recruitment would improve transparency.

Data Saturation

The manuscript states that recruitment continued until no new patterns emerged. However, the process used to determine data saturation is not described in detail. The authors should clarify how saturation was assessed.

Qualitative Rigor

The manuscript mentions reflexive journaling and independent coding, but the authors could further elaborate on strategies used to ensure credibility, dependability, and confirmability of the findings.

Reporting Guidelines

The authors may consider indicating whether the study followed qualitative reporting standards such as COREQ (Consolidated Criteria for Reporting Qualitative Research).

5. Results

The results section is clearly organized.

Suggestions:

The authors may consider including a summary table of themes and subthemes to improve readability. Some quotations are relatively long and could be shortened while maintaining their meaning. The narrative description of the themes occasionally repeats similar ideas; some sections could be made more concise.

6. Discussion

The discussion appropriately interprets the findings in relation to existing literature and theoretical frameworks.

Suggestions:

Some sections explaining the theoretical models are relatively long and could be more directly connected to the study findings. The discussion could more clearly indicate the novel contribution of the study, particularly the triangulation of stakeholder perspectives.

Additional references from recent studies in sub-Saharan Africa could strengthen the discussion.

7. Implications for Policy and Practice

The manuscript appropriately indicates policy implications for improving adolescent SRH service utilisation.

Suggestion:

The authors may consider providing more concrete recommendations for health systems, such as training of providers, strengthening youth-friendly services, or community engagement strategies.

8. Strengths and Limitations

The authors acknowledge several limitations, including the geographic scope of the study and potential social desirability bias.

Suggestion: The limitations section could also mention:

Possible selection bias due to convenience sampling.

Limitations in generalizability beyond the study region.

9. Conclusion

The conclusion summarizes the findings appropriately and reflects the evidence presented in the results.

Suggestion:

The authors may slightly strengthen the conclusion by emphasizing specific actionable recommendations for improving adolescent-friendly SRH services.

10. Data Availability Statement

The manuscript states that data will be available upon request due to the sensitive nature of the information.

Comment:

Since PLOS ONE generally encourages open data sharing, the authors should clarify whether anonymized transcripts or coded datasets could be deposited in a repository, or provide a stronger justification for restricted access.

Overall Recommendation

Minor Revision

The manuscript addresses an important topic and provides valuable insights into adolescent SRH service utilisation in Uganda. However, clarifications regarding methodological rigor, sampling procedures, and data availability are needed before the manuscript can be considered for publication.

Reviewer #3: This manuscript addresses an important public health issue. The triangulation of perspectives from adolescents, community leaders, and health providers is a notable strength and contributes useful contextual insight. The manuscript is generally well structured, and the qualitative approach is appropriate for the study objectives. However, several areas require clarification and refinement to strengthen coherence, methodological transparency, and interpretation of findings.

Reviewer #4: The authors are commended for investigating this important public health challenge of Sexual and Reproductive Health (SRH) service utilisation among this critical population group - of which the clarity of the stakeholders' perspective is required to ensure tailored targeted interventions, with consideration of the Regional (and sometimes, sub-Regional/ District) unique characteristics.

The manuscript is scientifically sound and mostly adheres to qualitative research principles. The study's focal strength being the triangulation of the stakeholders' perspectives.

The discussion and conclusions drawn, however, could be further enhanced by expanding on the review of the relevant literature to include findings of studies conducted in other sub-Sahara African (SSA) countries as well as other similar settings beyond the continent. The broader comparison and contrast with other studies would complement the balance of the narrow focus on studies conducted in Uganda/ East Africa - the only other African country's contrasted data is that of a study from South Africa.

The authors' application and analytical data interpretation was strengthened through the use of the Health Belief Model and Andersen’s Behavioural Model of Health Services Use - which enabled further exploration of the understanding of individual perceptions, enabling factors, and sociocultural contexts that conform to SRH service utilisation.

Methods: Given that ensuring trustworthiness is paramount in establishing the reliability of qualitative research findings, the other parameters of credibility, dependability and confirmability, need to be explicitly stated within the context of the research/ manuscript. [Only reflexivity and transferability were mentioned]. Cite the relevant literature/ sources.

General: Overall, the language use is appropriate and clear. Although, there are minor grammatical errors that need to be reviewed.

Line 152: rephrase to "... shaping adolescent health-seeking behaviour."

Line 283: replace "like" with "such as" - also amend where applicable throughout the manuscript.

References: Ensure consistency of the referencing method. There is a plethora of relevant literature sources that the authors can further include - see comment above regarding the inclusion of and comparison with studies conducted in other SSA countries and other similar settings outside the African Region.

**********

what does this mean?). If published, this will include your full peer review and any attached files.

If you choose “no”, your identity will remain anonymous but your review may still be made public.

**Do you want your identity to be public for this peer review?**  For information about this choice, including consent withdrawal, please see our Privacy Policy

Reviewer #1: **Yes:**  Philippe Poli Amubuomombe Poli

Reviewer #2: No

Reviewer #3: **Yes:**  Pelisa Nongena

Reviewer #4: **Yes:**  Dr. NRT (Neo) Ledibane, School of Health Systems and Public Health, Faculty of Health Sciences, University of Pretoria, Pretoria, South Africa

**********

[NOTE: If reviewer comments were submitted as an attachment file, they will be attached to this email and accessible via the submission site. Please log into your account, locate the manuscript record, and check for the action link "View Attachments". If this link does not appear, there are no attachment files.]

To ensure your figures meet our technical requirements, please review our figure guidelines: https://journals.plos.org/plosone/s/figures

You may also use PLOS’s free figure tool, NAAS, to help you prepare publication quality figures: https://journals.plos.org/plosone/s/figures#loc-tools-for-figure-preparation.

NAAS will assess whether your figures meet our technical requirements by comparing each figure against our figure specifications.

Attachment Submitted filename: Manuscript ID 26 00996.docx

Attachment Submitted filename: PONE-D-26-00996_12mar2026.docx

# Author comment: Author response to Decision Letter 1

**DOI:** 10.1371/journal.pone.0358996.r002

24 Apr 2026

Dear Editor and Reviewers,

We sincerely thank you for your thorough, rigorous, and constructive review of our manuscript. Your detailed feedback has been invaluable in significantly strengthening the clarity, methodological transparency, analytical depth, and overall quality of our work. We have carefully revised the manuscript in response to all comments. Below, we provide a detailed, point-by-point response, with corresponding line references to the revised manuscript. All major revisions and additions are highlighted in bold text for ease of review.

________________________________________

1. PLOS ONE Formatting and Style

Comment: Ensure the manuscript complies with PLOS ONE formatting guidelines.

Response: The manuscript has been fully revised to comply with PLOS ONE formatting requirements, including structure, headings, referencing style, and file naming conventions. The abstract now follows the structured format (Background, Methods, Results, Conclusion) as required.

________________________________________

2–4. Data Availability and Open Data Policy

Comment: Clarify data sharing, restrictions, and repository deposition. (Reviewers #2, #3, and #4 raised concerns about data availability.)

Response: We have substantially revised the data availability approach to align fully with PLOS ONE's open data policy:

• A fully de-identified qualitative dataset (focus group discussions and key informant interviews) has been prepared following established anonymization standards (e.g., BMJ 2010).

• All direct identifiers (names of participants, specific village names, health facilities, dates, and institutional affiliations) have been removed or generalized to protect participant confidentiality.

• The dataset has been deposited in a public repository .

• The Data Availability Statement has been updated to include the repository link and accession number.

• We confirm that written informed consent (and assent for minors) explicitly included permission for the use of anonymized data in research outputs and public repositories.

• No potentially identifiable information remains in the deposited dataset.

This ensures full compliance with PLOS ONE's requirement that data underlying findings be publicly accessible while preserving participant confidentiality.

Revised text: Data Availability Statement (Lines 487–490); Ethical Considerations (Lines 188–196).

________________________________________

Point-by-Point Responses to Reviewer Comments

We have consolidated comments from all four reviewers and respond to each below.

________________________________________

Abstract

Comment 1 (Reviewers #1 and #2): The abstract background is too general. It needs greater specificity, quantitative indicators, and a clearer rationale for the Busoga region and stakeholder approach.

Response: The abstract background has been completely revised to include:

• Specific quantitative indicators: 24% adolescent childbearing rate, 15% modern contraceptive prevalence among adolescents (Uganda Demographic and Health Survey 2022).

• Named barriers: sociocultural norms, poverty, limited health system capacity.

• Clear justification: the Busoga region is disproportionately affected by these challenges, yet no study has triangulated perspectives from adolescents, providers, and community/religious leaders.

Revised text: Abstract, Lines 13–20.

Comment 2 (Reviewer #1): Replace "suitable services" with a more precise term.

Response: "Suitable services" has been replaced with "adolescent-friendly SRH services" throughout the abstract and manuscript.

Revised text: Abstract, Line 17; also consistent throughout.

Comment 3 (Reviewers #1 and #2): The objective combines several aims, diluting clarity. It should more directly reflect the primary research purpose.

Response: The objective has been refined to focus on the single primary aim: to explore stakeholder perspectives to provide an integrated understanding of factors influencing adolescent SRH service utilization. The secondary elements (barriers, facilitators, strategies) now appear in the results and conclusion.

Revised text: Abstract, Lines 19–20.

Comment 4 (Reviewer #1): The abstract methods section is incomplete.

Response: The methods section has been expanded to include:

• Study setting (Iganga and Bugweri districts, Busoga region).

• Recruitment approach: adolescents through peer networks; key informants purposively selected.

• A clear statement of ethical approval and consent procedures.

Revised text: Abstract, Lines 22–28.

Comment 5 (Reviewers #1, #2, and #3): The results section lists themes descriptively but does not articulate the main analytical insight. The abstract and results should reflect the collapse of five themes into three.

Response: Thank you for this critical observation. We have substantially restructured the results to address this concern.

First, the original five themes have been collapsed into three analytically coherent themes that follow a clear logical progression from motivation → barriers → solutions:

Original Five Themes Collapsed into Three Themes

1. Positive orientation toward SRH prevention amid sexual self-discovery Theme 1: Positive orientation toward SRH prevention amid sexual self-discovery (motivation)

2. Persistent misconceptions and fears surrounding contraception ↓

3. Access barriers related to distance, privacy, and provider attitudes Theme 2: Barriers to SRH service utilization (barriers)

5. Sociocultural and system-level constraints ↓

4. Strong preferences for free, confidential, peer-informed, and community-based SRH services Theme 3: Preferences for adolescent-friendly and community-based services (solutions)

Second, the results section has been reframed to highlight the core analytical insight: adolescents are motivated to use SRH services, but this motivation is constrained by intersecting barriers, leading them to prefer alternative service models. The three themes are now presented within this analytical narrative rather than as a simple list.

Third, Theme 3 (Preferences) is now clearly articulated and highlighted in bold throughout the results section. This theme captures adolescents' strong preferences for:

• Free, confidential, and accessible services

• Community- and school-based delivery platforms

• Peer-led approaches (because "we believe our peers")

• Outreach programmes and integration with existing community structures (e.g., village health teams, religious gatherings)

Revised text: Abstract, Lines 30–36; Results, Lines 216–224 (theme overview) and Lines 359–381 (Theme 3: Preferences for adolescent-friendly and community-based services).

________________________________________

Methods (Data Collection, Recruitment, and Transparency)

Comment 6 (Reviewers #1, #2, and #3): Expand the data collection/procedure section to include recruitment and sampling processes, saturation rationale, and data security.

Response: The Methods section has been substantially expanded to include:

• Detailed recruitment processes for adolescents (via community mobilisers and youth peer leaders) and key informants (purposively selected based on institutional roles).

• Explicit saturation rationale: data collection continued until no new patterns emerged, achieved after 4 FGDs and 12 KIIs, confirmed through team debriefing and concurrent transcript review.

• Data security procedures: anonymization, password-protected devices, restricted access to the research team.

Revised text: Lines 99–162.

Comment 7 (Reviewers #1, #2, and #3): Explain how adolescents and key informants were identified and approached. Add inclusion/exclusion criteria, definitions of urban/rural residence, and whether any participants declined. Reviewer #2 specifically noted that the sampling strategy needed clarification, and Reviewer #3 noted that convenience sampling was incorrectly stated.

Response: This section has been significantly expanded. We have corrected the sampling description: it is purposive sampling, not convenience sampling.

• Adolescents were purposively sampled to capture diverse perspectives across gender, age, and residential contexts. Recruitment was facilitated through community mobilisers and youth peer leaders, with outreach in communities.

• Inclusion criteria for adolescents: age 13–19 years, residence in the study area for at least six months, and willingness to participate. No exclusion criteria were applied beyond these.

• Key informants were purposively selected based on formal institutional roles (health providers: nurses and clinical officers; religious leaders: clergy, pastors, imams; cultural leaders: clan leaders and elders) and recognized community authority.

• A small number of adolescents declined participation due to time constraints or discomfort discussing SRH in group settings. No refusals were recorded among key informants.

Revised text: Lines 110–146.

Comment 8 (Reviewers #1 and #2): Provide more operational detail for FGD procedures: group size, composition rules, presence of note-takers, language, and handling of sensitive topics.

Response: The following details have been added:

• Each FGD comprised 9–10 participants.

• Groups were stratified by sex and residence. Age stratification was implicit: female groups were younger (13–17 years); male groups were older (17–19 years) to reflect developmental stages and facilitate open discussion.

• Each session was facilitated by a trained moderator of the same sex as participants. A note-taker was present; no external observers were in the room.

• All FGDs were conducted in Lusoga, the local language.

• Sensitive topics were managed using neutral phrasing, probing techniques, and reassurance of confidentiality. Participants were reminded that they could skip any question or withdraw at any time.

Revised text: Lines 132–138.

Comment 9 (Reviewers #1, #2, and #3): Clarify how the team determined that sufficient saturation was reached for FGDs and KIIs.

Response: A clear saturation statement has been added:

• Sampling proceeded iteratively, with regular team debriefings and concurrent review of transcripts.

• Saturation was considered reached when no new themes emerged, categories were well-developed, and additional interviews were unlikely to yield meaningful new insights.

• This occurred after 4 FGDs and 12 KIIs, at which point data collection ceased.

Revised text: Lines 122–124 (sampling) and Lines 151–155 (analysis).

Comment 10 (Reviewer #4): Ensure trustworthiness parameters (credibility, dependability, confirmability, transferability) are explicitly stated within the context of the research. Cite relevant literature.

Response: Thank you for this important guidance. The Methods section has been revised to explicitly address all four trustworthiness criteria:

• Credibility: achieved through prolonged engagement during discussions, probing techniques, triangulation of multiple stakeholder perspectives, and back-translation of selected transcripts (Lines 132–138, 151–155).

• Dependability: ensured through regular team debriefings, independent coding by two researchers, and maintenance of a comprehensive audit trail (Lines 151–162).

• Confirmability: supported by reflexive journaling, documentation of assumptions and positionality, and grounding interpretations in participant accounts rather than researcher predispositions (Lines 162–168).

• Transferability: acknowledged that findings are primarily applicable to the Busoga region (Lines 470–471).

Revised text: Lines 132–138, 151–168, 470–471.

________________________________________

Results Section

Comment 11a (Reviewers #1 and #2): No detailed description of participants is provided. A table summarizing participant characteristics is essential.

Response: Two new tables have been added:

• Table 1: Composition of Focus Group Discussions (FGDs) by site, gender, mean age, schooling status, and number of participants.

• Table 2: Composition of Key Informant Interviews (KIIs) by participant group, gender, district, rural/urban location, and total participants.

Revised text: Lines 201–212 (Tables 1 and 2).

Comment 11b (Reviewers #1 and #2 – Summary table of themes and sub-themes): Reviewer #1 specifically noted: "The authors may consider including a summary table of themes and subthemes to improve readability." Reviewer #2 similarly suggested organizing the findings with a table.

Response: Thank you for this practical suggestion. We have included Table 3 in the revised manuscript, which provides a clear, at-a-glance summary of the three main themes and their corresponding sub-themes.

This table directly addresses Reviewer #1's request for improved readability and Reviewer #2's suggestion for better organization of findings. It appears immediately after the participant characteristics tables (Tables 1 and 2) and before the detailed thematic narrative, allowing readers to orient themselves before reading the full results.

Revised text: Lines 214–224 (Table 3).

Comment 11c (Reviewers #1, #2, and #3): Themes are presented descriptively; the analysis should explain how themes relate to each other. The collapse of five themes into three should be clearly shown.

Response: The results section has been restructured to present three interconnected themes that follow a clear analytical progression:

1. Theme 1: Positive orientation toward SRH prevention amid sexual self-discovery (motivation and agency).

2. Theme 2: Barriers to SRH service utilization (sub-themes: misconceptions, structural/interpersonal barriers, sociocultural/system constraints).

3. Theme 3: Preferences for adolescent-friendly and community-based services (solutions).

An explicit analytical pathway has been added: adolescents show motivation (Theme 1) → but face barriers (Theme 2) → leading them to prefer alternative service models (Theme 3).

Within Theme 3, we now clearly articulate adolescents' specific preferences, including: free and confidential services; community- and school-based delivery platforms; peer-led approaches ("because we believe our peers"); outreach programmes; and integration with existing community structures such as village health teams and religious gatherings.

Revised text: Lines 216–224 (overview), and throughout the Results section (Lines 233–381).

Comment 11d (Reviewers #1 and #2): Quotes lack sufficient detail. Use a standardized format.

Response: All quotes have been standardized to the following format: (Quote text, Participant group, FGD/KII number, district, rural/urban). For example: (Female adolescent, FGD 2, rural Iganga). This has been applied consistently throughout the Results section.

Revised text: Throughout Lines 233–381.

Comment 11e (Reviewer #3): The analysis sometimes merges perspectives without clearly distinguishing them. Provide clearer differentiation between stakeholder groups.

Response: The analysis has been revised to clearly distinguish perspectives:

• Adolescents: emphasize privacy, stigma, fear of judgment, and preferences for peer-led services.

• Health providers: highlight service capacity issues, stockouts, staffing shortages, and efforts to reach adolescents through outreach.

• Religious and cultural leaders: stress moral norms, early marriage practices, religious prohibitions on contraception, and the role of community guidance.

This differentiation is now explicit throughout the Results section.

Revised text: Lines 233–381 (e.g., separate quotes and analysis for each stakeholder group within each theme).

Comment 11f (Reviewers #1 and #3): Results are largely descriptive; interpretive insight is needed. Add reflexivity and analytical interpretation.

Response: Interpretive commentary has been added throughout the Results section, including:

• Parental silence: how limited parent–adolescent communication drives self-directed learning and reliance on peer networks.

• Misinformation: how fears of infertility and cancer, reinforced by community and religious narratives, outweigh perceived benefits of contraception.

• Barrier interaction: how distance, privacy, provider attitudes, and stockouts combine to create compounded access failures.

• Preference logic: why adolescents prefer community-based and peer-led services as a direct response to stigma and confidentiality concerns (explicitly developed in Theme 3).

Revised text: Lines 233–381 (interpretive statements added within each theme).

Comment 11g (Reviewers #1 and #2): The results section should briefly indicate whether saturation was achieved.

Response: A saturation statement has been added to the Results section:

Revised text: Lines 122-124, 151–155.

Comment 11h (Reviewer #3): The integration of systems and cultural factors (formerly Theme 5) reads as fragmented. Better highlight interactions.

Response: Theme 2 (which now incorporates the former Theme 5) has been substantially revised to show the interaction between religion, early marriage norms, poverty, and health system weaknesses. The sub-theme now explicitly demonstrates how:

• Religious norms and early marriage practices constrain adolescents' agency.

• Health system stockouts and staffing shortages compound these cultural barriers.

• The combination of normative expectations and service gaps leads to delayed or foregone care.

Revised text: Lines 277–358 (previously Theme 5, now integrated into Theme 2).

________________________________________

Discussion

Comment 12 (Reviewers #2, #3, and #4): The discussion would benefit from broader comparison with studies from other sub-Saharan African countries and similar settings outside Africa. Reviewer #4 specifically noted that only one study from South Africa was cited.

Response: The discussion has been substantially expanded to include comparisons with studies from Ethiopia, Nigeria, Ghana, Kenya, Tanzania, and other sub-Saharan African countries. New references have been added (References 4, 5, 6, 23, 24, 26). The discussion now explicitly situates our findings within the broader SSA evidence base while highlighting the unique contribution of multi-stakeholder triangulation in the Busoga context.

Revised text: Lines 400–445.

Comment 13 (Reviewer #2): Some sections explaining the theoretical models are relatively long and could be more directly connected to the study findings.

Response: The theoretical application sections have been shortened and more tightly integrated with specific findings from each theme. Direct links between HBM constructs (perceived barriers, perceived benefits) and Andersen's model (predisposing, enabling, need factors) are now explicitly mapped to participant quotes.

Revised text: Lines 420–445.

________________________________________

Implications for Policy and Practice

Comment 14 (Reviewer #2): Provide more concrete recommendations for health systems, such as training of providers, strengthening youth-friendly services, or community engagement strategies.

Response: The Implications section has been expanded to include specific, actionable recommendations:

• Health system level: targeted provider training in non-judgmental communication, dedicated adolescent service corners, flexible service hours, and privacy-enhancing infrastructure.

• Community level: structured engagement with parents, religious leaders, and cultural gatekeepers through dialogue forums and faith-based sensitization initiatives.

• School level: peer education programmes and integration of SRH information into existing curricula.

• Supply chain: strengthening commodity supply chain management to reduce stockouts, particularly for contraceptives in rural facilities.

Revised text: Lines 451–467.

________________________________________

Strengths and Limitations

Comment 15 (Reviewer #2): The limitations section could also mention possible selection bias due to sampling and limitations in generalizability beyond the study region.

Response: The limitations section has been expanded to explicitly acknowledge:

• Geographic scope – limited to two districts in Busoga, limiting transferability

• Context specificity – findings primarily grounded in Busoga region

• Social desirability bias – participants may have given socially acceptable responses

• Translation challenges – potential loss of nuance in translation from Lusoga to English

Revised text: Lines 470–475.

________________________________________

Conclusion

Comment 16 (Reviewers #1 and #2): The conclusion overgeneralizes findings to "similar low-resource settings" without sufficient caution. Emphasize specific actionable recommendations.

Response: The conclusion has been revised to:

• Emphasize that the findings are primarily applicable to the Busoga region of eastern Uganda.

• Note cautiously that the insights may be relevant to other contexts with comparable sociocultural norms, economic challenges, and health system constraints, but that direct replication requires contextual adaptation.

• Include specific actionable recommendations: decentralisation through community- and school-based platforms, outreach programmes, peer-led approaches, and confidentiality-enhancing service design.

Revised text: Lines 477–485.

________________________________________

Language and Grammar (Reviewer #4)

Reviewer Concern Action Taken

Abstract too general (Reviewer #1) Added quantitative indicators (24%, 15%) and specific barriers

Objective overloaded (Reviewer #1) Refined to single primary aim

Methods incomplete (Reviewer #1) Added setting, recruitment, ethics

Results list themes descriptively (Reviewers #1, #2, #3) Collapsed 5 themes into 3; added analytical pathway

Summary table of themes and sub-themes (Reviewers #1 and #2) Added Table 3 (Lines 214–224)

Theme 3 (Preferences) not clearly articulated Now highlighted in bold with specific preferences listed

Sampling strategy unclear; "convenience sampling" incorrect (Reviewers #2, #3) Corrected to purposive sampling; added detailed recruitment

Saturation rationale missing (Reviewers #1, #2, #3) Added explicit saturation statement

Participant characteristics table missing (Reviewers #1, #2) Added Tables 1 and 2

Quotes lack attribution (Reviewers #1, #2) Standardized quote format

Stakeholder perspectives merged (Reviewer #3) Differentiated perspectives clearly

Interpretive depth missing (Reviewers #1, #3) Added interpretive commentary throughout

Trustworthiness criteria incomplete (Reviewer #4) Added credibility, dependability, confirmability, transferability

Discussion too narrow (Reviewers #2, #3, #4) Expanded to include Ethiopia, Nigeria, Ghana, Kenya, Tanzania

Language errors (Reviewer #4) Corrected Line 152, Line 283, and throughout

References inconsistent (Reviewers #2, #3, #4) Standardized formatting; added recent SSA literature

Data availability restrictions (All reviewers) Deposited de-identified data in public repository; updated Data Availability Statement

Comment 17 (Reviewer #4): Minor grammatical errors need to be reviewed. Specific line edits:

• Line 121: rephrase to "... shaping adolescent health-seeking behaviour."

• Line 229: replace "like" with "such as" (and amend where applicable throughout the manuscript).

Response: These corrections have been made. Line 121 now reads "... shaping adolescent health-seeking behaviour." Line 229 and other instances of "like" have been changed to "such as" where appropriate. The entire manuscript has been carefully proofread for additional grammatical and typographical errors.

Revised text: Lines 121, 229, and throughout.

________________________________________

References

Comment 18 (Reviewers #2, #3, and #4): Ensure consistency of the referencing method. Include more recent literature and studies from other SSA countries.

Response: The reference list has been updated to ensure consistency in formatting (all now follow PLOS ONE style). New references from Ethiopia, Nigeria, Ghana, Kenya, and Tanzania have been added (References 4, 5, 6, 23, 24, 26). Older references have been retained where they represent foundational or landmark studies. All references are now within the last 10 years except for theoretical foundations (Health Belief Model, Andersen's Behavioural Model).

Revised text: References section (Lines 505–588).

________________________________________

Summary of Key Changes in Response to Reviewers

________________________________________

Additional Revisions Undertaken to Improve Quality

In addition to addressing all reviewer comments, we have made the following improvements:

• COREQ alignment: The manuscript has been reviewed against the COREQ checklist for qualitative studies, and all relevant items have been addressed (e.g., researcher characteristics, relationship with participants, theoretical framework).

• Theory integration: The Health Belief Model and Andersen's Behavioural Model are now more explicitly integrated into the analysis and discussion.

• Language and readability: The manuscript has been carefully proofread for clarity, grammar, and consistency.

• Correction of sampling terminology: All references to "convenience sampling" have been removed and replaced with accurate descriptions of purposive sampling throughout the manuscript and response letter.

• Trustworthiness criteria: Credibility, dependability, confirmability, and transferability are now explicitly addressed in the Methods section with citations (Lincoln & Guba, 1985; Nowell et al., 2017).

________________________________________

Final Statement

We believe these revisions have substantially improved the manuscript's methodological rigor, analytical depth, transparency, and alignment with PLOS ONE's qualitative reporting standards. In particular, the collapse of five themes into three analytically coherent themes (Theme 1: Motivation, Theme 2: Barriers, Theme 3: Preferences), the addition of Table 3 (summary of themes and sub-themes), the correction of the sampling description to purposive sampling, and the explicit inclusion of trustworthiness criteria have significantly strengthened the manuscript.

We are deeply grateful for the reviewers' insightful feedback, which has helped us produce a stronger and more impactful manuscript. We have also addressed the data availability concerns by depositing a fully de-identified dataset in a public repository and updating the Data Availability Statement accordingly.

We hope the revised manuscript is now acceptable for publication in PLOS ONE.

Thank you for your consideration.

Sincerely,

Noah Nyende (Corresponding Author)

on behalf of all co-authors

Attachment Submitted filename: Response to Reviewers.docx

# Peer review: Decision Letter 1

**DOI:** 10.1371/journal.pone.0358996.r003

**Authors:** Joseph Matovu

1 Jul 2026

Dear Dr. Nyende,

Thank you for submitting your manuscript to PLOS ONE. After careful consideration, we feel that it has merit but does not fully meet PLOS ONE’s publication criteria as it currently stands. Therefore, we invite you to submit a revised version of the manuscript that addresses the points raised during the review process.

Please submit your revised manuscript by Aug 15 2026 11:59PM. If you will need more time than this to complete your revisions, please reply to this message or contact the journal office at plosone@plos.org. When you're ready to submit your revision, log on to https://www.editorialmanager.com/pone/ and select the 'Submissions Needing Revision' folder to locate your manuscript file.

- A letter that responds to each point raised by the academic editor and reviewer(s). You should upload this letter as a separate file labeled 'Response to Reviewers'.
- A marked-up copy of your manuscript that highlights changes made to the original version. You should upload this as a separate file labeled 'Revised Manuscript with Track Changes'.
- An unmarked version of your revised paper without tracked changes. You should upload this as a separate file labeled 'Manuscript'.

If you would like to make changes to your financial disclosure, please include your updated statement in your cover letter. Guidelines for resubmitting your figure files are available below the reviewer comments at the end of this letter.

If applicable, we recommend that you deposit your laboratory protocols in protocols.io to enhance the reproducibility of your results. Protocols.io assigns your protocol its own identifier (DOI) so that it can be cited independently in the future. For instructions see: https://journals.plos.org/plosone/s/submission-guidelines#loc-laboratory-protocols. Additionally, PLOS ONE offers an option for publishing peer-reviewed Lab Protocol articles, which describe protocols hosted on protocols.io. Read more information on sharing protocols at https://plos.org/protocols?utm_medium=editorial-email&utm_source=authorletters&utm_campaign=protocols.

As the corresponding author, your ORCID iD is verified in the submission system and will appear in the published article. PLOS supports the use of ORCID, and we encourage all coauthors to register for an ORCID iD and use it as well. Please encourage your coauthors to verify their ORCID iD within the submission system before final acceptance, as unverified ORCID iDs will not appear in the published article. *Only*  the individual author can complete the verification step; PLOS staff *cannot*  verify ORCID iDs on behalf of authors.

We look forward to receiving your revised manuscript.

Kind regards,

Joseph KB Matovu, Ph.D.

Academic Editor

PLOS One

Journal Requirements:

1. If the reviewer comments include a recommendation to cite specific previously published works, please review and evaluate these publications to determine whether they are relevant and should be cited. There is no requirement to cite these works unless the editor has indicated otherwise.

2. Please review your reference list to ensure that it is complete and correct. If you have cited papers that have been retracted, please include the rationale for doing so in the manuscript text, or remove these references and replace them with relevant current references. Any changes to the reference list should be mentioned in the rebuttal letter that accompanies your revised manuscript. If you need to cite a retracted article, indicate the article’s retracted status in the References list and also include a citation and full reference for the retraction notice.

[Note: HTML markup is below. Please do not edit.]

Reviewer's Responses to Questions

**Comments to the Author**

Reviewer #1: All comments have been addressed

Reviewer #2: All comments have been addressed

Reviewer #3: (No Response)

Reviewer #4: All comments have been addressed

**********

2. Is the manuscript technically sound, and do the data support the conclusions?

Reviewer #1: Yes

Reviewer #2: Yes

Reviewer #3: Yes

Reviewer #4: Yes

**********

3. Has the statistical analysis been performed appropriately and rigorously?

Reviewer #1: Yes

Reviewer #2: Yes

Reviewer #3: N/A

Reviewer #4: Yes

**********

4. Have the authors made all data underlying the findings in their manuscript fully available?

The PLOS Data policy

Reviewer #1: Yes

Reviewer #2: Yes

Reviewer #3: No

Reviewer #4: Yes

**********

5. Is the manuscript presented in an intelligible fashion and written in standard English?

Reviewer #1: Yes

Reviewer #2: Yes

Reviewer #3: Yes

Reviewer #4: Yes

**********

Reviewer #1: In my opinion, the major concerns raised during the review process have been adequately addressed, and the manuscript now makes a meaningful contribution to the understanding of adolescent sexual and reproductive health service utilization in low-resource settings.

I therefore recommend acceptance of the manuscript for publication in PLOS ONE.

Sincerely,

Reviewer

Reviewer #2: I have carefully reviewed the revised manuscript and the authors’ responses to the previous review comments. The authors have made substantial improvements to the manuscript by strengthening the background, clarifying the study rationale, improving the methodological description, and enhancing the presentation and interpretation of the qualitative findings. The multi-stakeholder perspective adopted in this study provides valuable insights into adolescent sexual and reproductive health service utilization in the Busoga region of Uganda and has important implications for policy and practice in similar low-resource settings.

The qualitative design is appropriate for the research objectives, the analytical approach is adequately described, and the conclusions are supported by the data presented. The manuscript is well organized and written in a clear and accessible manner.

I have only a few minor editorial observations. The authors should ensure consistency in reporting participant numbers, terminology (e.g., utilization/utilisation), and formatting throughout the manuscript and carefully proofread the final version to eliminate any remaining typographical or formatting inconsistencies. These issues do not affect the scientific validity or interpretation of the findings.

I have no concerns regarding research ethics or publication ethics based on the information provided. Overall, I believe the manuscript makes a meaningful contribution to the literature on adolescent sexual and reproductive health services and is suitable for publication in PLOS ONE.

Recommendation: Accept.

Reviewer #3: The manuscript would benefit from a professional editor/ proof reading. There are minor issues that need attention.

Reviewer #4: To the Authors,

Thank you and well done for sufficiently addressing the review comments.

All the best!

**********

what does this mean?). If published, this will include your full peer review and any attached files.

If you choose “no”, your identity will remain anonymous but your review may still be made public.

**Do you want your identity to be public for this peer review?**  For information about this choice, including consent withdrawal, please see our Privacy Policy

Reviewer #1: No

Reviewer #2: No

Reviewer #3: No

Reviewer #4: No

**********

[NOTE: If reviewer comments were submitted as an attachment file, they will be attached to this email and accessible via the submission site. Please log into your account, locate the manuscript record, and check for the action link "View Attachments". If this link does not appear, there are no attachment files.]

To ensure your figures meet our technical requirements, please review our figure guidelines: https://journals.plos.org/plosone/s/figures

You may also use PLOS’s free figure tool, NAAS, to help you prepare publication quality figures: https://journals.plos.org/plosone/s/figures#loc-tools-for-figure-preparation.

NAAS will assess whether your figures meet our technical requirements by comparing each figure against our figure specifications.

Attachment Submitted filename: ACCEPTANCE OF THE MANUSCRIPT.docx

Attachment Submitted filename: PONE-D-26-00996_R1.pdf.docx

# Author comment: Author response to Decision Letter 2

**DOI:** 10.1371/journal.pone.0358996.r004

6 Jul 2026

Response to Reviewers

Manuscript PONE-D-26-00996R1

“Stakeholder perspectives on adolescent sexual and reproductive health service utilization in Uganda’s Busoga region: A qualitative study”

Dear Dr. Matovu,

Thank you for the opportunity to revise our manuscript and for the constructive feedback from you and the four reviewers. We appreciate the reviewers’ positive assessment of the revised manuscript and have carefully addressed the remaining editorial points raised. Below we provide a point-by-point response to each comment. Reviewer and editor comments are shown in italics, and our responses follow.

Academic Editor – Journal Requirements

Comment 1

If the reviewer comments include a recommendation to cite specific previously published works, please review and evaluate these publications to determine whether they are relevant and should be cited.

Response: None of the reviewers recommended specific additional works to cite in this round of review. We have therefore not added any new citations in response to this requirement.

Comment 2

Please review your reference list to ensure that it is complete and correct. If you have cited papers that have been retracted, please include the rationale for doing so in the manuscript text, or remove these references and replace them with relevant current references.

Response: We have reviewed all 28 references in the manuscript and confirm that none has been retracted.

Reviewer #1

In my opinion, the major concerns raised during the review process have been adequately addressed, and the manuscript now makes a meaningful contribution to the understanding of adolescent sexual and reproductive health service utilization in low-resource settings. I therefore recommend acceptance of the manuscript for publication in PLOS ONE.

Response: We thank the reviewer for this positive assessment and for their time and constructive input across both rounds of review.

Reviewer #2

I have only a few minor editorial observations. The authors should ensure consistency in reporting participant numbers, terminology (e.g., utilization/utilisation), and formatting throughout the manuscript and carefully proofread the final version to eliminate any remaining typographical or formatting inconsistencies.

Response: We thank the reviewer for this careful reading and have addressed each of the three points raised:

• Participant numbers: The number of key informant interviews was previously written inconsistently as “twelve” in the Methods (Participants, Data collection, and Data analysis subsections) while appearing as the numeral “12” in the Results section and Table 2. We have standardized this to the numeral “12” throughout the manuscript, consistent with reporting numbers of 10 or greater as numerals.

• Terminology: The term “utilisation” (British spelling) appeared once in the Results section while “utilization” (American spelling) was used throughout the rest of the manuscript. We have corrected this instance so that “utilization” is used consistently throughout.

• Formatting/proofreading: We identified that British and American spelling conventions were mixed throughout the manuscript (e.g., “characterised/characterized,” “organisation/organization,” “recognised/recognized,” “minimise/minimize,” “familiarisation/familiarization,” “mobilisation/mobilization”). We have standardized spelling to American English throughout, consistent with the predominant convention used in the original submission. We also standardized abbreviation use in the Discussion (e.g., consistent use of “SRH” and “HBM” after first definition) and re-proofread the full manuscript for residual typographical inconsistencies.

Reviewer #3

The manuscript would benefit from a professional editor/proof reading. There are minor issues that need attention.

Response: We thank the reviewer for this feedback. We have carefully proofread the entire manuscript and corrected the spelling and formatting inconsistencies described above (standardizing British/American spelling conventions, correcting numeral usage for participant counts, and correcting reference formatting). We have also reviewed the manuscript line by line for residual typographical errors.

Reviewer #4

Thank you and well done for sufficiently addressing the review comments. All the best!

Response: We are grateful for the reviewer’s time and encouraging feedback throughout the review process.

Summary of changes

In summary, the following changes were made in this revision:

1. Standardized the reported number of key informant interviews to the numeral “12” throughout the Methods and Results (previously “twelve” in Methods).

2. Corrected a single instance of “utilisation” to “utilization” in the Results for consistency with the rest of the manuscript.

3. Standardized British/American spelling conventions to American English throughout the manuscript.

4. Standardized the reporting of residence duration in the inclusion criteria (“six months” to “6 months”) for consistency with numeral use for measurements.

5. Confirmed that no cited references have been retracted.

We thank the Academic Editor and all four reviewers again for their time and valuable feedback, which we believe has strengthened the manuscript.

Sincerely,

Dr. Nyende and co-authors

Attachment Submitted filename: Response_to_Reviewers (1).docx

# Peer review: Decision Letter 2

**DOI:** 10.1371/journal.pone.0358996.r005

**Authors:** Devon Hensel

8 Sep 2026

Stakeholder perspectives on adolescent sexual and reproductive health service utilization in Uganda’s Busoga region: A qualitative study

PONE-D-26-00996R2

Dear Dr. Nyende,

We’re pleased to inform you that your manuscript has been judged scientifically suitable for publication and will be formally accepted for publication once it meets all outstanding technical requirements.

Within one week, you’ll receive an e-mail detailing the required amendments. When these have been addressed, you’ll receive a formal acceptance letter and your manuscript will be scheduled for publication.

An invoice will be generated when your article is formally accepted. Please note, if your institution has a publishing partnership with PLOS and your article meets the relevant criteria, all or part of your publication costs will be covered. Please make sure your user information is up-to-date by logging into Editorial Manager at Editorial Manager® and clicking the ‘Update My Information' link at the top of the page. For questions related to billing, please contact billing support.

If your institution or institutions have a press office, please notify them about your upcoming paper to help maximize its impact. If they’ll be preparing press materials, please inform our press team as soon as possible -- no later than 48 hours after receiving the formal acceptance. Your manuscript will remain under strict press embargo until 2 pm Eastern Time on the date of publication. For more information, please contact onepress@plos.org.

Kind regards,

Devon J. Hensel, M.S., Ph.D.

Academic Editor

PLOS One

Additional Editor Comments (optional):

Reviewers' comments:

Reviewer's Responses to Questions

**Comments to the Author**

Reviewer #1: All comments have been addressed

Reviewer #2: All comments have been addressed

**********

2. Is the manuscript technically sound, and do the data support the conclusions?

Reviewer #1: Yes

Reviewer #2: Yes

**********

3. Has the statistical analysis been performed appropriately and rigorously?

Reviewer #1: Yes

Reviewer #2: Yes

**********

4. Have the authors made all data underlying the findings in their manuscript fully available?

The PLOS Data policy

Reviewer #1: Yes

Reviewer #2: Yes

**********

5. Is the manuscript presented in an intelligible fashion and written in standard English?

Reviewer #1: Yes

Reviewer #2: Yes

**********

Reviewer #1: The revised version demonstrates improved coherence, stronger analytical presentation, and better alignment with the reporting standards expected for qualitative research in PLOS ONE. The content of the manuscript sounds scientifically acceptable.

Reviewer #2: Thank you for the opportunity to review the revised manuscript. The authors have adequately addressed the major substantive comments raised in the previous round, and the manuscript has been substantially improved. However, a few minor editorial and consistency issues remain and should be addressed before publication. In particular, the reporting of participant numbers is still inconsistent in several places (e.g., “twelve 12” KIIs), and some typographical and spelling inconsistencies remain despite the authors’ response indicating that these issues were corrected throughout the manuscript. Please conduct a final comprehensive proofreading to ensure consistency in participant numbers, terminology, American/British English spelling, and formatting throughout the manuscript. These remaining issues are minor and do not raise further substantive concerns regarding the study. I therefore recommend minor revision to address these editorial issues before the manuscript is considered for publication.

**********

what does this mean?). If published, this will include your full peer review and any attached files.

If you choose “no”, your identity will remain anonymous but your review may still be made public.

**Do you want your identity to be public for this peer review?**  For information about this choice, including consent withdrawal, please see our Privacy Policy

Reviewer #1: No

Reviewer #2: No

**********

# Editor report: Acceptance letter

**DOI:** 10.1371/journal.pone.0358996.r006

**Authors:** Devon Hensel

PONE-D-26-00996R2

PLOS One

Dear Dr. Nyende,

I'm pleased to inform you that your manuscript has been deemed suitable for publication in PLOS One. Congratulations! Your manuscript is now being handed over to our production team.

At this stage, our production department will prepare your paper for publication. This includes ensuring the following:

* All references, tables, and figures are properly cited

* All relevant supporting information is included in the manuscript submission,

* There are no issues that prevent the paper from being properly typeset

You will receive further instructions from the production team, including instructions on how to review your proof when it is ready. Please keep in mind that we are working through a large volume of accepted articles, so please give us a few days to review your paper and let you know the next and final steps.

Lastly, if your institution or institutions have a press office, please let them know about your upcoming paper now to help maximize its impact. If they'll be preparing press materials, please inform our press team within the next 48 hours. Your manuscript will remain under strict press embargo until 2 pm Eastern Time on the date of publication. For more information, please contact onepress@plos.org.

You will receive an invoice from PLOS for your publication fee after your manuscript has reached the completed accept phase. If you receive an email requesting payment before acceptance or for any other service, this may be a phishing scheme. Learn how to identify phishing emails and protect your accounts at https://explore.plos.org/phishing.

If we can help with anything else, please email us at customercare@plos.org.

Thank you for submitting your work to PLOS One and supporting open access.

Kind regards,

PLOS One Editorial Office Staff

on behalf of

Dr. Devon J. Hensel

Academic Editor

PLOS One
